# Developing Cyclic Peptomers as Broad-Spectrum Type III Secretion System Inhibitors in Gram-Negative Bacteria

**DOI:** 10.1128/AAC.01690-20

**Published:** 2021-06-17

**Authors:** Hanh N. Lam, Tannia Lau, Adam Lentz, Jessica Sherry, Alejandro Cabrera-Cortez, Karen Hug, Annalyse Lalljie, Joanne Engel, R. Scott Lokey, Victoria Auerbuch

**Affiliations:** a Department of Microbiology and Environmental Toxicology, University of California Santa Cruz, Santa Cruz, California, USA; b Department of Chemistry and Biochemistry, University of California Santa Cruz, Santa Cruz, California, USA; c Department of Medicine, University of California San Francisco, San Francisco, California, USA

**Keywords:** *Chlamydia*, *Pseudomonas aeruginosa*, *Salmonella*, *Yersinia*, antibiotic resistance, cyclic peptide, inhibitor, type III secretion system

## Abstract

Antibiotic-resistant bacteria are an emerging global health threat. New antimicrobials are urgently needed. The injectisome type III secretion system (T3SS), required by dozens of Gram-negative bacteria for virulence but largely absent from nonpathogenic bacteria, is an attractive antimicrobial target. We previously identified synthetic cyclic peptomers, inspired by the natural product phepropeptin D, that inhibit protein secretion through the *Yersinia* Ysc and Pseudomonas aeruginosa Psc T3SSs but do not inhibit bacterial growth. Here, we describe the identification of an isomer, 4EpDN, that is 2-fold more potent (50% inhibitory concentration [IC_50_] of 4 μM) than its parental compound. Furthermore, 4EpDN inhibited the *Yersinia* Ysa and the Salmonella SPI-1 T3SSs, suggesting that this cyclic peptomer has broad efficacy against evolutionarily distant injectisome T3SSs. Indeed, 4EpDN strongly inhibited intracellular growth of Chlamydia trachomatis in HeLa cells, which requires the T3SS. 4EpDN did not inhibit the unrelated twin arginine translocation (Tat) system, nor did it impact T3SS gene transcription. Moreover, although the injectisome and flagellar T3SSs are evolutionarily and structurally related, the 4EpDN cyclic peptomer did not inhibit secretion of substrates through the Salmonella flagellar T3SS, indicating that cyclic peptomers broadly but specifically target the injectisome T3SS. 4EpDN reduced the number of T3SS needles detected on the surface of Yersinia pseudotuberculosis as detected by microscopy. Collectively, these data suggest that cyclic peptomers specifically inhibit the injectisome T3SS from a variety of Gram-negative bacteria, possibly by preventing complete T3SS assembly.

## INTRODUCTION

Antibiotic resistance is of great concern to global public health. Bacterial pathogens have evolved numerous mechanisms to survive treatment with clinically available antibiotics (1). Alternative therapies against multidrug-resistant strains of so-called ESKAPE pathogens (Enterococcus faecium, Staphylococcus aureus, Klebsiella pneumoniae, Acinetobacter baumannii, Pseudomonas aeruginosa, and Enterobacter species) are urgently needed. Various strategies have been explored to avoid the so-called antimicrobial apocalypse (2). One promising approach is to inhibit bacterial virulence mechanisms to disarm pathogens without affecting nonpathogenic members of the microbiota or environmental bacteria (3, 4). This approach has the potential to not only control infection but to do so in a way that preserves the integrity of the microbiome, which is beneficial for human health and is often the source of antibiotic resistance genes (5, 6).

The type III secretion system (T3SS), a needle-like injectisome apparatus, is required for virulence in many Gram-negative pathogens, including Salmonella, *Yersinia*, Chlamydia, and the ESKAPE pathogen P. aeruginosa. The T3SS is largely absent from commensal bacteria, making it a good target for virulence blocker antimicrobials. Based on phylogenetic analysis of core T3SS proteins, T3SSs were classified into seven families (7, 8). However, T3SSs have many highly conserved structural components (9). T3SS genes are typically encoded on virulence plasmids or pathogenicity islands, indicative of horizontal gene transfer (10); therefore, phylogeny of T3SSs does not follow organismal phylogeny. Phylogenetic analysis suggests that the injectisome T3SS evolved from the flagellar system (7, 8). Indeed, the flagellar basal body is a secretion system, referred to as the flagellar T3SS, that secretes flagellin and other cargo into the extracellular space in order to build the flagellar filament to power motility. The flagellar and injectisome T3SSs share several conserved basal body and export apparatus components (9). However, the injectisome T3SS does not mediate motility, but instead delivers effector proteins into target host cells.

The T3SS is one of the most complex protein assemblies in prokaryotes involving multiple proteins assembled in an ordered manner. All T3SSs are composed of an external, hollow needle attached to a basal body made up of the outer membrane secretin SctC (11) and the inner membrane component SctD (YscD in *Yersinia*) (12), as well as the export apparatus SctRSTUVJ (13). Following the formation of the two SctC and SctD membrane rings, the cytosolic complex composed of YscKQLNO associates with the membrane rings and export apparatus to make an active secretion system (12). The early substrates, such as SctF (called YscF in *Yersinia*), are then secreted (14), allowing SctF to polymerize to form the T3SS needle. The middle substrates, the needle tip protein SctA and translocators SctE and SctB, are then secreted and make contact with host cells to trigger secretion of the late substrates, the effector proteins that alter host defenses (15). A number of regulators of the T3SS have been described in different bacteria, including those whose secretion by the T3SS alters their cytoplasmic concentration and therefore their activity. One example is Pseudomonas ExsE, which sequesters the T3SS master regulator EsxA through a partner switching mechanism, until host cell contact is made and ExsE is secreted, relieving ExsA repression and potentiating an increase in T3SS gene transcription (16, 17).

A number of small molecules, antibodies, and vaccines have been studied for T3SS targeted therapies (18). Despite showing promising effects on the T3SS *in vitro* and in animal models, only one antibody-based therapy has entered clinical trials. A bispecific antibody, MEDI3902, against the P. aeruginosa T3SS needle tip protein PcrV (SctA) and the Psl exopolysaccharide is effective against both acute and chronic infection models and is in phase II clinical trials for prevention of ventilator-associated pneumonia (19, 20). However, antibodies must be administered intravenously, so chemical inhibitors of the T3SS are needed.

As narrow-spectrum antimicrobials require more precise diagnostics, broad-spectrum T3SS inhibitors would be more valuable clinically than those only able to target one bacterial species. In addition, most mammalian pathogens that utilize a T3SS require their T3SS only during growth within, but not outside, the host animal. However, the *Chlamydiae*, which cause lung, genital, and eye infections, are obligate intracellular bacteria, and their T3SS is strictly required for their growth (21). Interestingly, the Chlamydia T3SS belongs to its own T3SS family (7, 8). Here, we identify a derivative of a synthetic cyclic peptomer family of T3SS inhibitors (22) that can inhibit the T3SS machinery of three evolutionarily distinct T3SS families used by five different bacterial species to cause human disease, including the Ysc (P. aeruginosa Psc, Y. pseudotuberculosis Ysc), Inv-Mxi-Spa (Y. enterocolitica Ysa, Salmonella enterica serovar Typhimurium SPI-1), and *Chlamydiales* (Chlamydia trachomatis) families. While having significant breath of activity among various injectisome T3SSs, these inhibitors do not affect bacterial growth or other secretion systems such as the flagellar T3SS or the twin-arginine translocation system.

## RESULTS

### Structure-activity relationship study of cyclic peptomers.

Previously, we identified a group of cyclic peptomers that inhibited secretion of substrates from Y. pseudotuberculosis and P. aeruginosa T3SSs but did not inhibit bacterial growth, motility, or HeLa cell metabolism (22). These results suggested a potential for development of the cyclic peptomers as pathogen-specific virulence blockers. Based on dose-response curves and concentration of half maximal inhibition (IC_50_) of the P. aeruginosa T3SS, 1EpDN (previously named EpD1,2N) was chosen for structure-activity relationship (SAR) analysis. The compounds used in SAR analysis are listed in Table 1.

**TABLE 1:** Compounds synthesized and used in this study

Simplified name	Full name/side chain identity (abbreviation of the 6 side chains)	Exact mass	Reference/source
1EpDN	EpD1,2N/propylamine, benzylamine, d-Leu, l-Ile, l-Leu, and d-Phe (PBDLLD)	732.46	22
1EpDN 1Sar	EpD1,2N 1Sar/sarcosine, benzylamine, d-Leu, l-Ile, l-Leu, and d-Phe (SarBDLLD)	704.43	This study
1EpDN 2Sar	EpD1,2N 2Sar/propylamine, sarcosine, d-Leu, l-Ile, l-Leu, and d-Phe (PSarDLLD)	656.43	This study
1EpDN 3Ala	EpD1,2N 3Ala/propylamine, benzylamine, d-Ala, l-Ile, l-Leu, and d-Phe (PB,d-Ala,LLD)	690.41	This study
1EpDN 4Ala	EpD1,2N 4Ala/propylamine, benzylamine, d-Leu, l-Ala, l-Leu, and d-Phe (PBD,l-Ala,LD)	690.41	This study
1EpDN 5Ala	EpD1,2N 5Ala/propylamine, benzylamine, d-Leu, l-Ile, l-Ala, and d-Phe (PBDL,l-Ala,D)	690.41	This study
1EpDN 6Ala	EpD1,2N 6Ala/propylamine, benzylamine, d-Leu, l-Ile, l-Leu, and d-Ala (PBDL,l,d-Ala)	656.43	This study
2EpDN	2-EpD1,2N/propylamine, benzylamine, l-Leu, l-Ile, l-Leu, and d-Phe (PBLLLD)	732.46	This study
3EpDN	3-EpD1,2N/propylamine, benzylamine, d-Leu, l-Ile, d-Leu, and d-Phe (PBDLDD)	732.46	This study
4EpDN	4-EpD1,2N/propylamine, benzylamine, l-Leu, l-Ile, d-Leu, and d-Phe (PBLLDD)	732.46	This study
5EpDN	5-EpD1,2N/propylamine, benzylamine, l-Leu, l-Ile, d-Leu, and l-Phe (PBLLDL)	732.46	This study
6EpDN	6-EpD1,2N/propylamine, benzylamine, d-Leu, l-Ile, d-Leu, and l-Phe (PBDLDL)	732.46	This study
7EpDN	7-EpD1,2N/propylamine, benzylamine, l-Leu, l-Ile, l-Leu, and l-Phe (PBLLLL)	732.46	This study
8EpDN	8-EpD1,2N/propylamine, benzylamine, d-Leu, l-Ile, l-Leu, and l-Phe (PBDLLL)	732.46	This study
9EpDN	9-EpD1,2N/propylamine, benzylamine, l-Leu, d-Ile, d-Leu, and l-Phe (PBLDDL) (enantiomer of 1EpDN)	732.46	This study
4EpDN 1Sar	4-EpD1,2N 1Sar/sarcosine, benzylamine, l-Leu, l-Ile, d-Leu, and d-Phe (SarBLLDD)	704.43	This study
4EpDN 2Sar	4-EpD1,2N 2Sar/propylamine, sarcosine, l-Leu, l-Ile, d-Leu, and d-Phe (PSarLLDD)	656.43	This study

**FIG 1:**
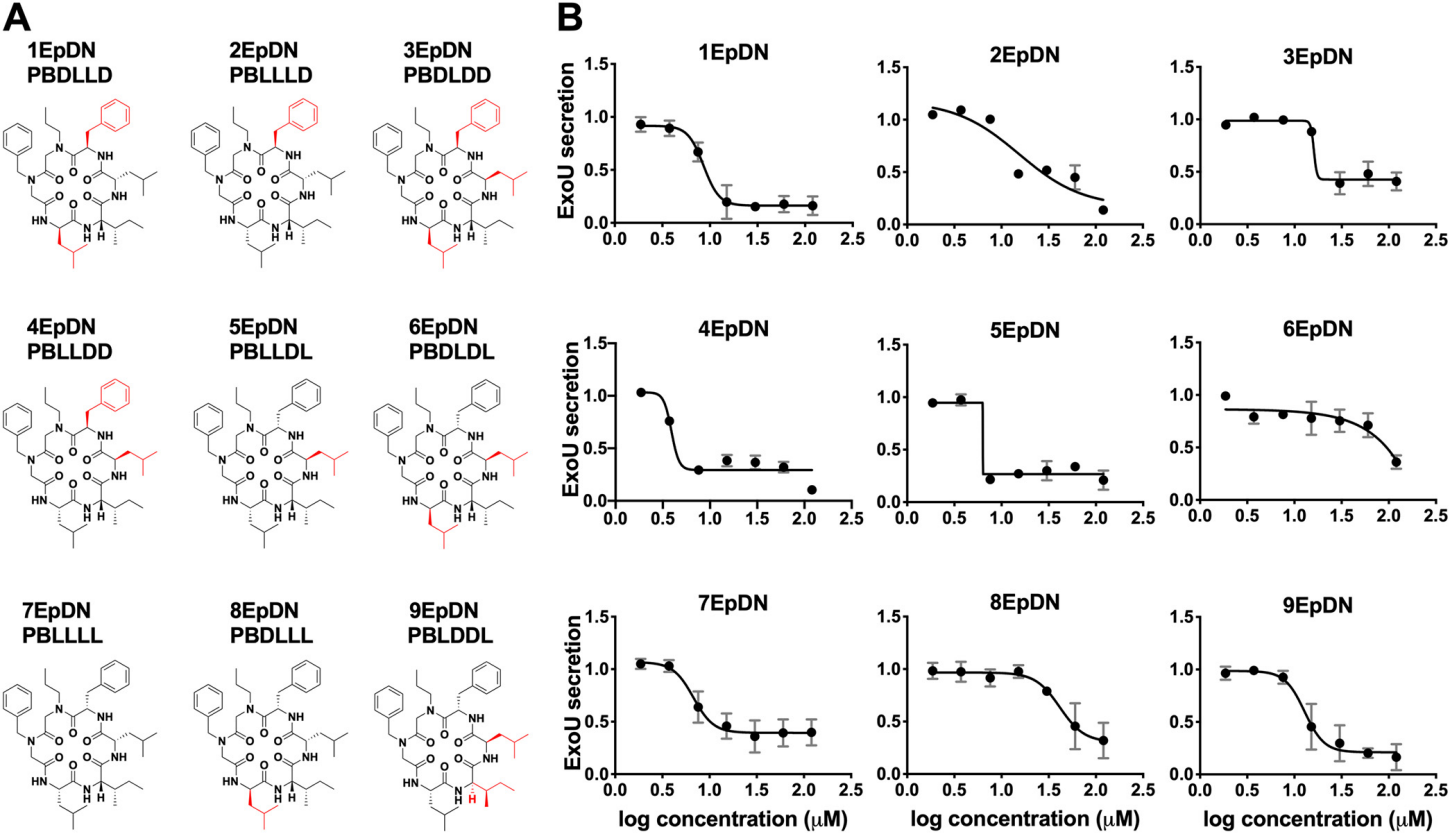
Stereochemistry scan of cyclic peptomers results in a more potent derivative, 4EpDN. (A) Structures of 1EpDN stereoisomers. Isomers were generated from different combinations of four side chains at positions 3 to 6. Numbers preceding compounds were used to distinguish the different isomers and the conformation of the four side chains. The d-amino acid side chain is shown in red. (B) WT P. aeruginosa PA103 was grown under T3SS-inducing conditions with increasing concentrations of cyclic peptomer isomers. Secretion of T3SS cargo into the culture supernatant was assessed by precipitating secreted proteins and visualizing them with Coomassie blue. ExoU band intensities were quantified and normalized to that of the DMSO control. The results are from at least two independent experiments. Nonlinear curve fitting is shown to depict the trend of inhibition. Error bars are standard errors of the mean.

**FIG 2:**
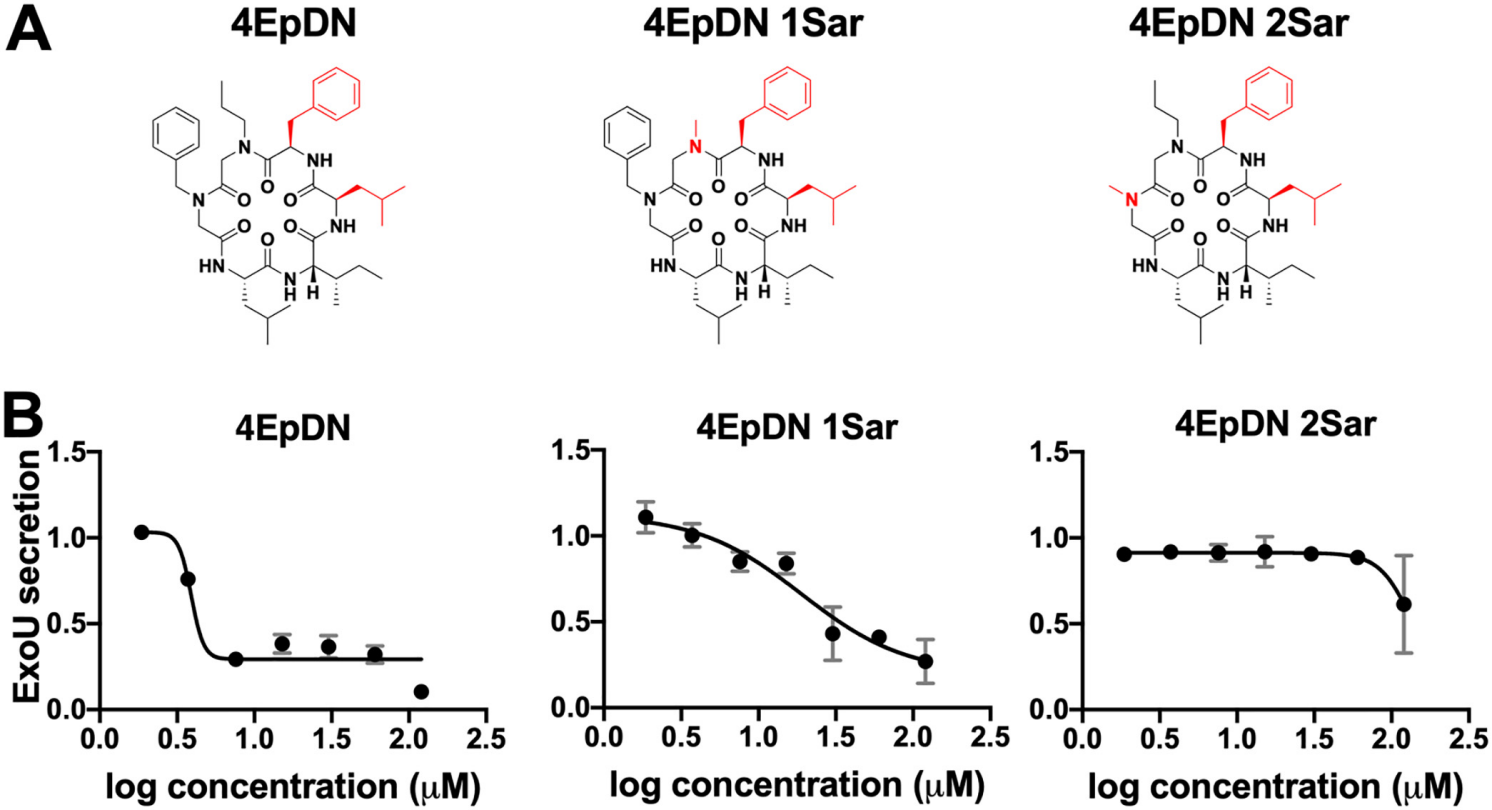
Sarcosine replacement of 4EpDN at position 1 or 2 eliminates activity. (A) Structures of 4EpDN and its derivatives, 4EpDN 1Sar and 4EpDN 2Sar. The d-amino acid side chain is shown in red. (B) WT P. aeruginosa PA103 was grown under T3SS-inducing conditions with increasing concentrations of compounds. Secretion of T3SS cargo into the culture supernatant was assessed on SDS-PAGE gel. ExoU band intensities were visualized with Coomassie blue, quantified, and normalized to that of the DMSO control. The results are from at least two independent experiments. Error bars are standard errors of the mean.

We first assessed the effect of alanine replacement at each of the six positions of the parent scaffold, 1EpDN. Note that because peptoids have side chains appended to a nitrogen atom rather than carbon as in amino acids, positions 1 and 2 were synthesized with *N*-methylglycine, also known as sarcosine (Sar), as the peptoid equivalent of alanine (Ala). Ala or Sar replacement at any of the six positions resulted in significant loss of activity, suggesting that all side chains contribute to the activity (see Fig. S1 in the supplemental material). Next, we carried out a stereochemistry scan, in which different combinations of l- and d-amino acids at positions 3 to 6 were generated. The parent compound, 1EpDN, has propylamine, and benzylamine at positions 1 and 2, and d-Leu, l-Ile, l-Leu, and d-Phe at positions 3 to 6. For the stereochemistry scan, we will refer to 1EpDN as PBDLLD. While most stereoisomers had the same or reduced T3SS inhibitory activity, 4EpDN (PBLLDD) showed improved activity, with an IC_50_ of ∼4 μM compared to the parent compound IC_50_ of ∼8 μM (Fig. 1A and B). Replacement of position 1 (4EpDN 1Sar) or position 2 (4EpDN 2Sar) with Sar significantly reduced activity of 4EpDN (Fig. 2A and B). 4EpDN and 4EpDN 2Sar were used as an active compound and a negative control, respectively, in most follow-up experiments. Importantly, 4EpDN and 4EpDN 2Sar did not affect bacterial viability in broth culture (Fig. S2).

### Cyclic peptomers do not inhibit secretion through the twin-arginine translocation (Tat) system.

**FIG 3:**
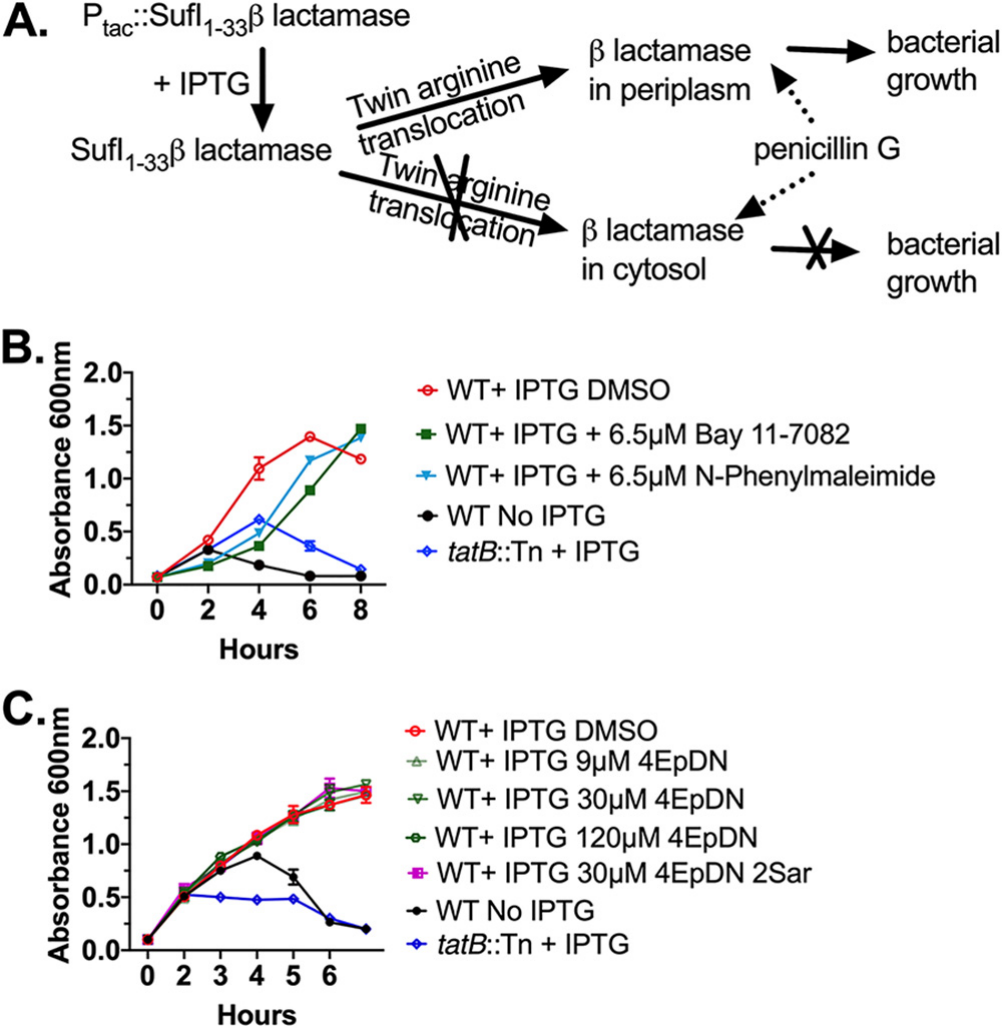
Cyclic peptomers do not affect the twin arginine translocation (Tat) system. (A) Y. pseudotuberculosis expressing a SufI-β-lactamase Tat reporter incubated in the presence of penicillin G will only grow if the Tat system remains functional. (B) Y. pseudotuberculosis SufI-β-lactamase reporters were treated with the Tat inhibitors Bay 11-7082, *N*-phenyl maleimide, or DMSO, and culture optical density was measured. WT refers to bacteria expressing a functional Tat secretion system. A mutant strain with a transposon insertion in the *tatB* gene serves as a control. (C) The same assay as in panel B was repeated in the presence of cyclic peptomers or DMSO. The result was from two independent replicates. Error bars are standard errors of the mean.

In order to determine if the cyclic peptomer 4EpDN inhibits the activity of secretion systems completely unrelated to the T3SS, we sought to assess the impact of cyclic peptomers on the twin arginine translocation (Tat) system. We chose to use Y. pseudotuberculosis for this because its Tat secretion system is well studied (23, 24). The Tat system translocates fully folded substrates across the inner membrane, while the T3SS translocates partially unfolded substrates across the inner, outer, and target host cell membranes (25). To monitor Tat secretion system activity, a reporter strain expressing an IPTG (isopropyl-β-d-thiogalactopyranoside)-inducible β-lactamase TEM-1 domain fused to the signal peptide of the SufI Tat substrate (24) was constructed. Following IPTG induction, β-lactamase confers resistance to the β-lactam peptidoglycan-targeting antibiotic penicillin G when the SufI-β-lactamase reporter has successfully translocated into the periplasm (Fig. 3A). The presence of known Tat inhibitors, Bay 11-7082 or *N*-phenylmaleimide (26), strongly reduced growth of bacteria after 4 and 6 h, while growth of bacterial cultures treated with cyclic peptomers was similar to that of the dimethyl sulfoxide (DMSO) control (Fig. 3B and C). These results suggested that 4EpDN does not inhibit the Tat secretion system.

### The 4EpDN cyclic peptomer inhibits secretion of T3SS substrates from the Inv-Mxi-Spa T3SS family but does not inhibit secretion through the flagellar T3SS.

The T3SSs were classified into seven families based on phylogenetic analysis (7, 8). We previously showed that cyclic peptomers inhibited the Ysc T3SS family found in P. aeruginosa and *Yersinia* (Fig. 1 and 2) (22). In order to test whether cyclic peptomers are active against other T3SS families, we evaluated the effect of cyclic peptomers on the Inv-Mxi-Spa T3SS in Y. enterocolitica and Salmonella enterica serovar Typhimurium.

**FIG 4:**
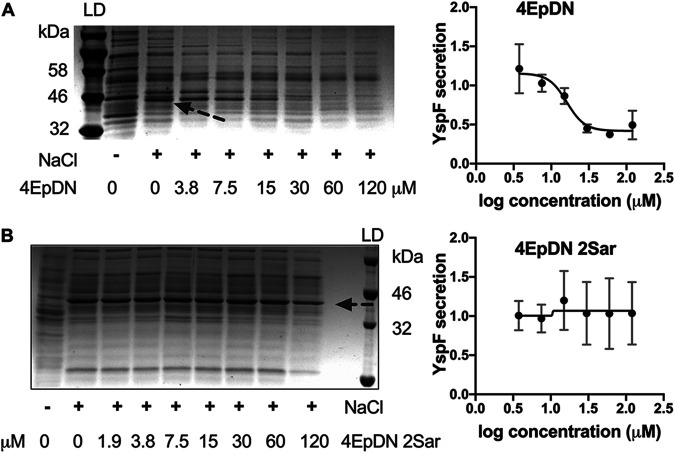
Effect of cyclic peptomers on secretion of *Yersinia* Ysa T3SS substrates. (A and B) Y. enterocolitica serotype O:8 was grown under T3SS-inducing conditions with increasing concentrations of cyclic peptomer isomers, 4EpDN (A) and 4EpDN 2Sar (B). Secretion of T3SS cargo into the culture supernatant was assessed by precipitating secreted proteins and visualizing them with Coomassie blue. Arrow points to the YscF protein band. YspF band intensities were quantified and normalized to that of the DMSO control. Representative gel images and quantification of YspF are shown. The results are from two independent experiments. Error bars are standard errors of the mean.

The Y. enterocolitica Ysa system, a chromosomally encoded T3SS, is distinct from the *Yersinia* Ysc T3SS and contributes to Y. enterocolitica colonization of the terminal ileum and gastrointestinal-associated tissues (27, 28). A Y. enterocolitica mutant that lacks expression of the Ysa T3SS (Δ*ysaT*) was used as a negative control, while a mutant lacking the Ysc T3SS (Δ*yscL*) (29) was used to evaluate the effect of compounds specifically on the Ysa system. Secretion of the Ysa effector protein YspF was quantified. 4EpDN inhibited secretion of YspF in a dose-dependent manner, while 4EpDN 2Sar did not affect its secretion (Fig. 4). Together, these results suggest that cyclic peptomers are active against both the Ysc and Ysa T3SSs in *Yersinia*.

**FIG 5:**
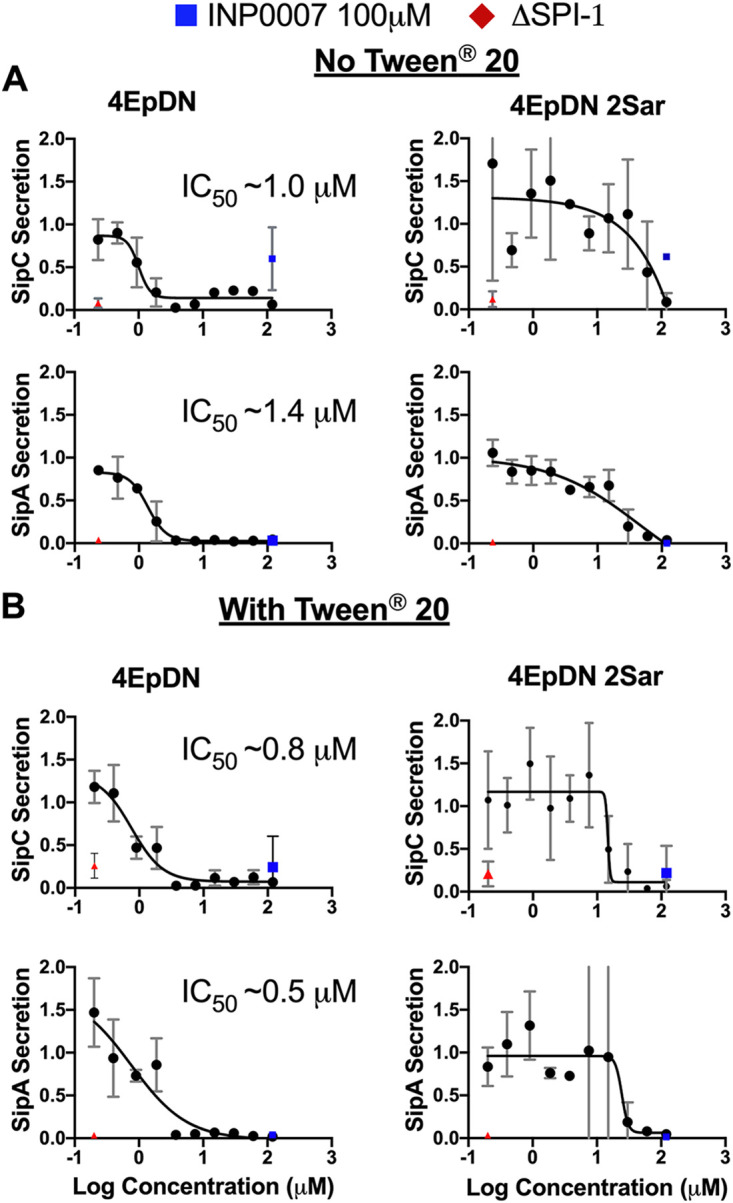
Cyclic peptomers inhibit the Salmonella SPI-1 T3SS. Salmonella enterica Typhimurium was grown with increasing concentrations of cyclic peptomer isomers. Secretion of SPI-1 T3SS cargo into the culture supernatant was assessed by precipitating secreted proteins and visualizing them with Coomassie blue. SipA and SipC band intensities were quantified and normalized to that of the DMSO control. (A and B) The experiments were carried out without the detergent Tween 20 (A) or with Tween 20 (B). A ΔSPI-1 Salmonella mutant and INP0007, a known SPI-1 inhibitor (64), were used as controls. The results are from at least two independent experiments. Error bars are standard errors of the mean.

In order to evaluate whether the cyclic peptomers are active against T3SSs distinct from the Ysc T3SS outside the *Yersinia* genus, we tested cyclic peptomer efficacy in Salmonella. Salmonella employs two T3SSs during infection, with the SPI-1 T3SS belonging to the Inv-Mxi-Spa T3SS family (7, 8). Inhibition of SPI-1 T3SS effector protein SipC and SipA (30–32) secretion by 4EpDN was observed at ∼1 μM and ∼1.4 μM, respectively, while 4EpDN 2Sar showed inhibition of SipC and SipA secretion only at concentrations greater than 30 μM (Fig. 5). It has previously been shown that compound aggregates can act as promiscuous inhibitors with nonspecific activity (33, 34). To rule out that 4EpDN activity was due to compound aggregation, we chose to measure Salmonella type III secretion in the presence of detergent. Comparison of various nonionic detergents (NP-40, Tween 20, and Triton X-100) at different concentrations suggested that Tween 20 at 0.003% was the highest concentration of detergent to have a minimal effect on secretion of effector proteins (Fig. S3); therefore, Tween 20 at this concentration was used for further analysis. Addition of Tween 20 did not reduce activity of 4EpDN but slightly increased it (Fig. 5), suggesting that activity of the cyclic peptomers does not result from aggregated compounds.

As 4EpDN inhibited both the Ysc and Inv-Mxi-Spa T3SS families, we tested whether this cyclic peptomer could inhibit the flagellar T3SS, which is the most distantly related T3SS family based on previous phylogenetic analysis (7). Conveniently, Salmonella expresses the SPI-1 and its flagellar system under the same conditions *in vitro* (rich media). This allowed us to investigate effects of cyclic peptomers on both the SPI-1 T3SS and flagellar systems under the same culture conditions. Because of the conservation between the injectisome and flagellar T3SSs, flagellar substrates can be secreted through both systems. Therefore, secretion of flagellar substrates (FliC and FliD) was quantified in both wild-type (WT) and ΔSPI-1 strains to distinguish secretion through both the SPI-1 T3SS and flagellar system (WT strain) or only through the flagellar system alone (ΔSPI-1 strain). 4EpDN inhibited FliC and FliD secretion in WT Salmonella at concentrations of ≥60 μM and ≥3.75 μM, respectively (Fig. S4), consistent with the ability of the SPI-1 T3SS being able to secrete flagellar substrates. However, 4EpDN only inhibited FliC and FliD secretion at high concentrations (≥60 μM) in the ΔSPI-1 mutant, with unfavorable dose-response curves compared to WT Salmonella. This suggests that the inhibitory effect of 4EpDN on FliD secretion in the WT strain was mainly through inhibition of its secretion through the SPI-1 T3SS. 4EpDN 2Sar had no significant effect on FliC secretion or FliD secretion. These data suggest that the cyclic peptomer 4EpDN does not significantly inhibit substrate secretion through the flagellar T3SS in Salmonella but strongly inhibits the SPI-1 T3SS under the same conditions.

### The 4EpDN cyclic peptomer affects the T3SS needle.

**FIG 6:**
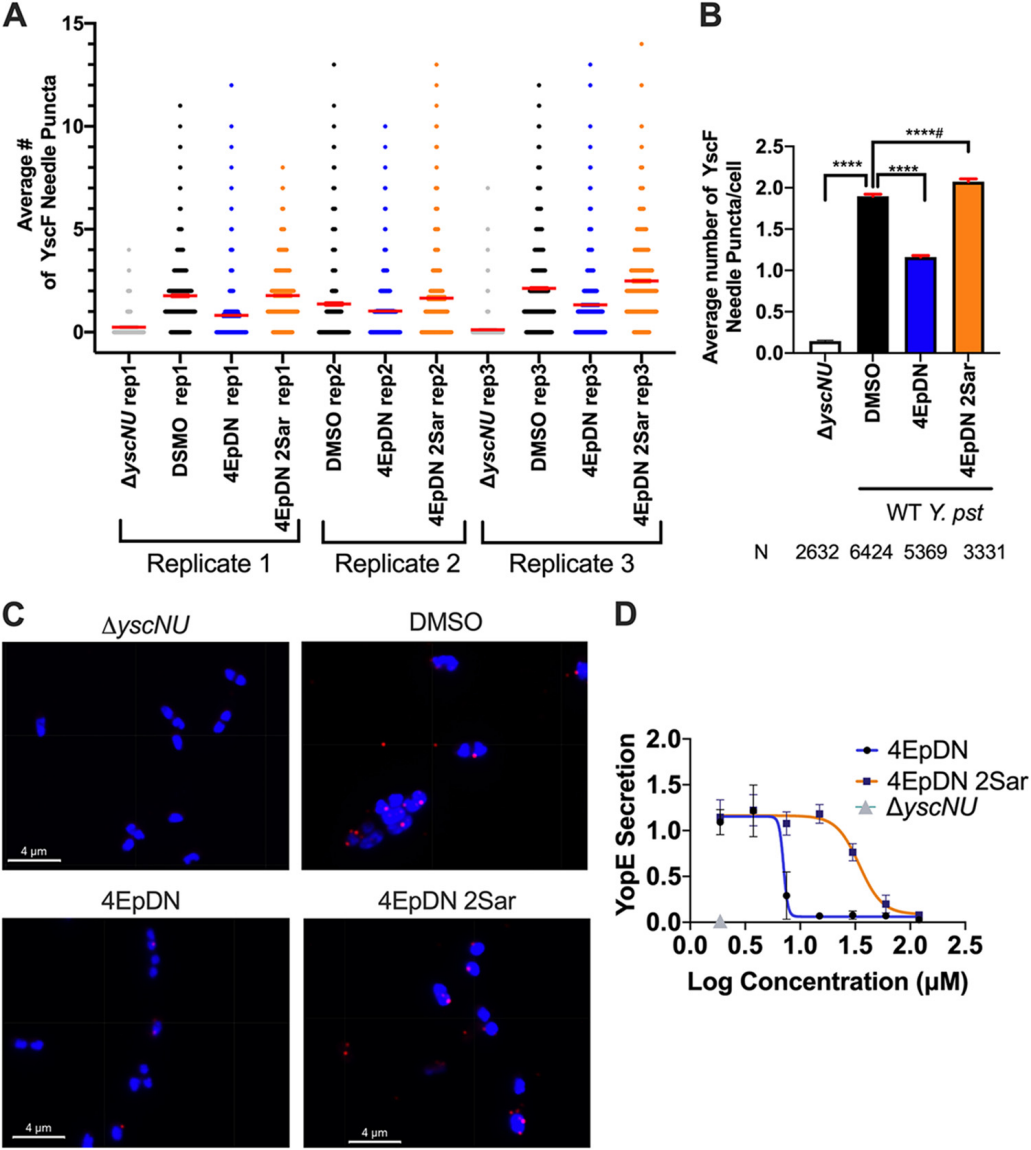
YscF puncta visualization using immunofluorescence. Y. pseudotuberculosis was grown under T3SS-inducing conditions (low Ca^2+^) in the presence of 60 μM cyclic peptomers or an equivalent volume of DMSO. A mutant lacking *yscN* and *yscU* was used as a negative control. (A) Scatterplot of YscF puncta/cell for the three replicates. Means ± the standard error of the mean (SEM) are shown in red. The width of distribution of points is proportional to the number of data points at the Y value. (B) Mean number of puncta/cell after treatment for all replicates combined ± SEM. (C) Representative images of YscF puncta in different conditions. (Imaris software displays grid lines within the images; they are not physical lines on the samples.) (D) Secretion of effector YopE in low-calcium medium on the presence of different concentrations of 4EpDN and 4EpDN 2Sar in WT Y. pseudotuberculosis stained with Coomassie stain and quantified. The data represent three independent experiments. The nonparametric Kruskal-Wallis test with Dunn’s multiple-comparison test was used. ****, *P* < 0.0001; ****, *P* < 0.0001, but the trend is in the reversed direction.

In order to determine how 4EpDN might inhibit type III secretion, we tested whether the cyclic peptomer inhibits assembly of the T3SS. We chose to use *Yersinia* for these experiments because of the existing microscopy tools to monitor assembly of T3SS components. The T3SS basal body must be assembled prior to T3SS substrate secretion (12, 35, 36). In *Yersinia*, the T3SS basal body component YscD (SctD) is an inner membrane ring protein that is conserved among injectisome T3SSs, but has low sequence homology with the flagellar ortholog FliG (9). The absence of YscD at the inner membrane prevents assembly of other T3SS machinery (YscL, YscK, YscQ) (12, 37) and secretion of T3SS substrates (38). We used a Y. enterocolitica strain expressing a YscD allele translationally fused with enhanced green fluorescent protein (EGFP) to visualize the effect of compounds on YscD assembly (12). 4EpDN caused only a modest reduction in the number of YscD puncta (Fig. S5). Once the T3SS basal body is assembled and functional, the next stage of T3SS assembly is polymerization of the T3SS needle subunit SctF (YscF in *Yersinia*) to form the T3SS needle (14). To determine whether the cyclic peptomers affect T3SS needle formation, we used an anti-YscF antibody to measure the number of YscF puncta at the bacterial surface by immunofluorescence microscopy. We found a 2-fold reduction in YscF puncta in the presence of 60 μM 4EpDN compared to the DMSO control (Fig. 6A to C). These data suggest that cyclic peptomers affect the assembly or stability of T3SS needles, ultimately dampening secretion of effector proteins.

### The 4EpDN cyclic peptomer does not inhibit transcription and secretion of the negative regulator ExsE.

**FIG 7:**
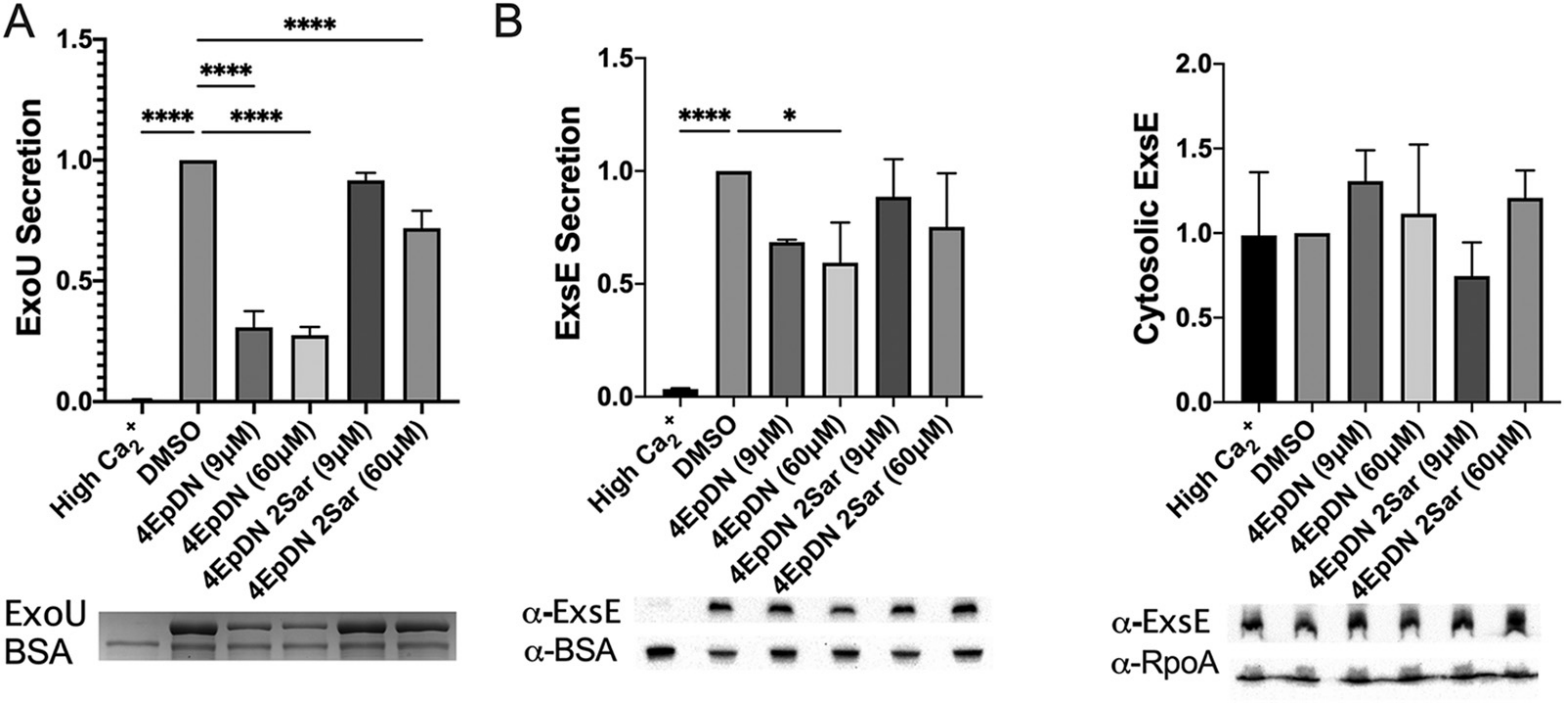
The cyclic peptomer 4EpDN inhibits secretion of the effector protein ExoU but not the regulator ExsE. PA103 was grown under T3S-inducing conditions in the presence of 9 μM or 60 μM cyclic peptomers or DMSO. (A) Secretion of ExoU was visualized using Coomassie blue and quantified. (B) In the same samples, Western blotting was carried out for secreted ExsE in the supernatant and ExsE in the cell pellets. BSA and RpoA were used as loading controls. Data were from three independent experiments. One-way ANOVA with Dunnett’s multiple-comparison test was used. *, *P* < 0.05; ****, *P* < 0.0001 compared to DMSO. Error bars are standard errors of the mean.

The cyclic peptomers did not decrease expression of T3SS genes in Salmonella or Pseudomonas (Fig. S6), suggesting that they do not act at the level of T3SS gene expression. In some bacteria with T3SSs, such as *Yersinia* and Pseudomonas, secretion of negative regulators through the T3SS leads to upregulation of T3SS gene expression (16, 17, 39–42). Thus, the observation that the cyclic peptomers did not affect Pseudomonas T3SS gene expression is surprising. To investigate this discrepancy, we observed the effect of 4EpDN on the P. aeruginosa negative regulator ExsE, which when secreted, relieves repression of the ExsA T3SS master regulator (17). 4EpDN significantly inhibited secretion of the effector protein ExoU by ∼75% at either 9 or 60 μM, while 4EpDN 2Sar had no effect (Fig. 7). In contrast, 4EpDN did not significantly inhibit ExsE secretion at 9 μM and only inhibited ExsE secretion ∼40% at 60 μM. This result explains the lack of impact of 4EpDN on the ExsE, which negatively regulates ExsA-mediated transcription. A major difference between ExsE and ExoU in terms of their protein properties is their size; ExsE is ∼9 kDa, while ExoU is ∼74 kDa. Taken together, these data suggest that 4EpDN may inhibit secretion of larger T3SS cargo more robustly than smaller cargo.

### Cyclic peptomers block Chlamydia infection.

**FIG 8:**
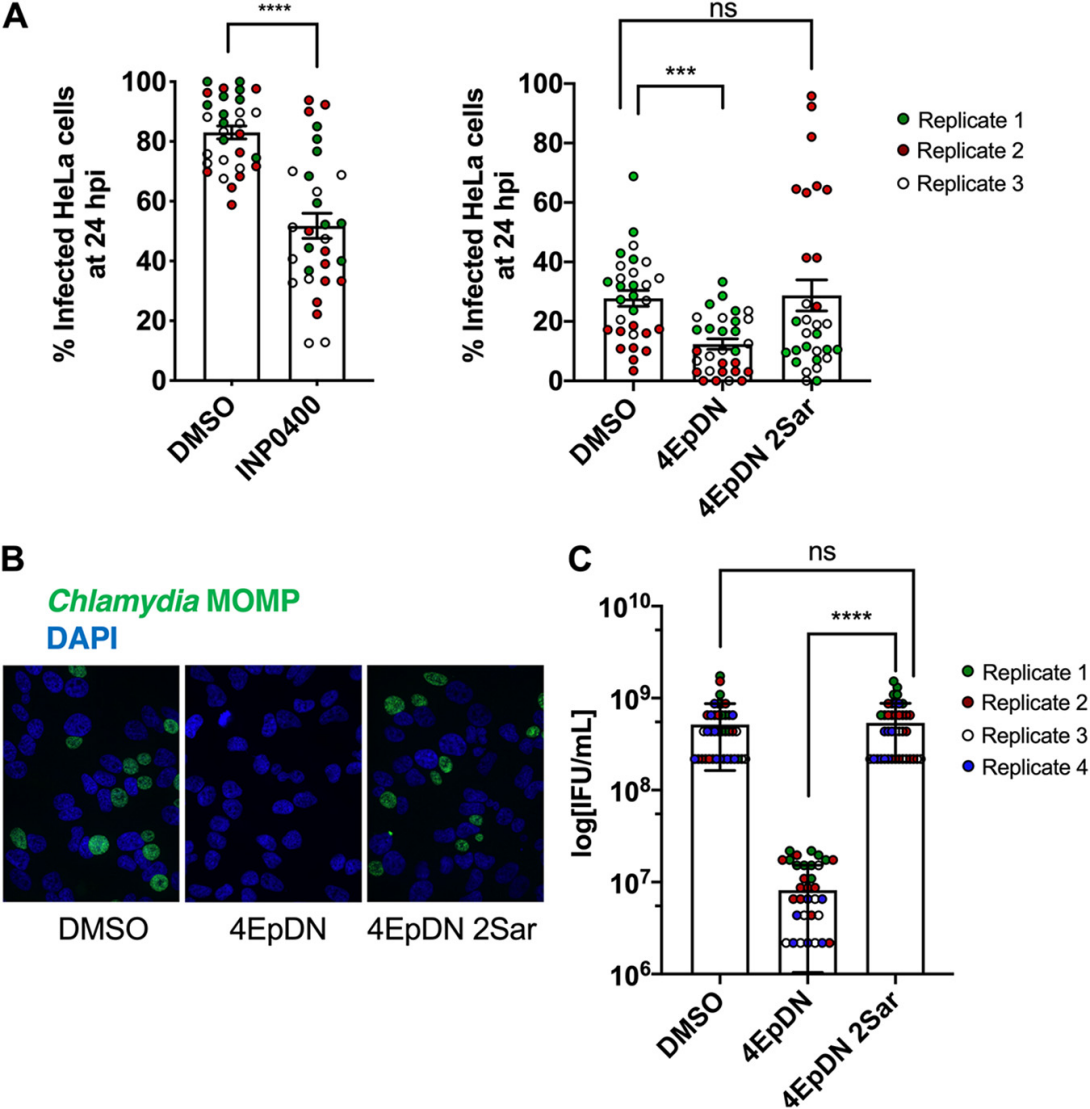
The cyclic peptomer 4EpDN inhibits Chlamydia infection. (A) HeLa cells were infected with C. trachomatis L2 at a multiplicity of infection (MOI) of 3 (left-hand panel) or 1 (right-hand panel) in the presence of 9 μM cyclic peptomers, 30 μM INP0400, or DMSO. Cells were stained for the Chlamydia major outer membrane protein (MOMP) and nucleic acids (DAPI) and imaged after 24 h of infection to determine the number of infected cells (primary infection). The Mann-Whitney test was used. (B and C) Infectious elementary bodies (EB) were harvested after 48 h of HeLa cell infection in the presence of inhibitors and were used to infect fresh HeLa cells without applying inhibitors (secondary infection). After 24 h, cells were imaged as in panel A. Representative images (B) and infectious units/ml (C) are shown from three to four independent experiments. Error bars are standard errors of the mean. The Kruskal-Wallis test with Dunn’s multiple-comparison test was used. ***, *P* < 0.0005; ****, *P* < 0.0001; ns: not significant.

In order to evaluate whether the 4EpDN cyclic peptomer can disarm bacterial virulence, we examined the effect of this compound on Chlamydia infection, as this pathogen requires the T3SS for infection and growth within human cells. The chlamydial life cycle involves two major bacterial forms, the extracellular infectious elementary bodies (EBs) and the intracellular replicative reticulate bodies (RBs). EBs are infectious and abundant around 48 h postinoculation (hpi). RBs are noninfectious and abundant at 24 hpi. Upon entry, EBs discharge preloaded T3SS effectors and are taken up into a membrane-bound compartment (the inclusion) where they differentiate into RBs, secrete additional T3SS effectors and replicate, and then redifferentiate into EBs. The initial stages of infection were assessed by quantifying the number of inclusions per cell at 24 hpi. In contrast, production of infectious progeny, which assays RB-EB redifferentiation and release of EBs, was assayed by collecting EBs at 48 hpi, infecting fresh monolayers for 24 hpi. INP0400, a known T3SS inhibitor was used as a control (43). 4EpDN but not 4EpDN 2Sar decreased primary inclusion formation ∼50% but inhibited formation of infectious progeny ∼98% (Fig. 8). These data show that the 4EpDN cyclic peptomer can completely block the chlamydial life cycle in human cells, which is dependent on the T3SS.

## DISCUSSION

In this study, we showed that the cyclic peptomer 4EpDN is a broad-spectrum injectisome T3SS inhibitor that disrupts T3SS assembly and secretion of T3SS effectors. 4EpDN inhibits secretion through the T3SS of a number of pathogens, including the nosocomial ESKAPE pathogen Pseudomonas aeruginosa, enteropathogenic *Yersinia*, and Salmonella, with IC_50_ in the low μM range. 4EpDN does not inhibit secretion from two other secretion systems—the flagellar T3SS and the Tat system. The 4EpDN cyclic peptomer has only a small effect on assembly of the basal body component YscD in the plasma membrane but inhibits T3SS needle assembly. Importantly, 4EpDN can completely block the ability of the obligate intracellular pathogen Chlamydia trachomatis to propagate in human cells, which requires the T3SS.

Through alanine and stereochemistry scans, we identified 4EpDN, a cyclic peptomer with an IC_50_ of 4 μM in inhibiting secretion of T3SS effector proteins in P. aeruginosa and 1 μM in inhibiting the Salmonella SPI-1 T3SS. Compared to previously published T3SS inhibitors (Table 2), this low μM activity is encouraging. The only published T3SS inhibitors with comparable IC_50_ are the phenoxyacetamides (MBX 2359 and its optimized derivatives), which inhibit P. aeruginosa T3SS secretion at 1 to 3 μM (44). Stereoisomers of 1EpDN showed a wide range of potencies, suggesting that differences in their three-dimensional structures affect their biological activity. 9EpDN is a true enantiomer of 1EpDN, with an IC_50_ of ∼13 μM, a lower potency than the 1EpDN parental compound’s ∼8 μM. Importantly, the activity of these isomers does not positively correlate with solubility (Fig. S7, Table S1), indicating that the observed activity is due to a specific molecular reaction rather than a nonspecific biophysical effect due to aggregation. Furthermore, the presence of nonionic detergent did not adversely affect the activity of compounds. These data suggest that 4EpDN is an active cyclic peptomer with specific T3SS inhibitory activity.

**TABLE 2:** Efficacy of cyclic peptomers and other type III secretion system inhibitors

Compound^*a*^	T3SS family and species								
	Psc/Ysc		Inv-Mxi-Spa		Ssa-Esc	Chlamydiales	Hrc1	Fla	
	PA	Ysc	Ysa	SPI-1	EPEC/EHEC	Chlamydia	PS	fliC	Reference/source
4EpDN	3.9 ExoU^*b*^	∼7.5 YopE^*b*^	16.1 YspF^*b*^	1 SipAC^*b*^		∼9^*c*^		NE^*d*^	This study
4EpDN 2Sar	139.5 ExoU^*b*^			∼30 SipAC^*b*^		NE^*d*^	NE^*d*^	This study
1EpDN	8.2 ExoU^*b*^	14.3 YopE^*b*^							22
MBX1641 Phe^*g*^	10 ExoS^*b*^					∼10^*c*^			65
MBX2359 Phe MBX2401 Phe	2.5 ExoS^*b*^ 1.2 ExoS^*b*^								44
Hydroxybenzimidazoles	∼3.5 ExsA^*e*^	∼3.9 LcrF^*e*^							66
INP1750 HQ^*g*^	∼80^*c*^	12.4 YopE^*e*^ EC_50_				25 MIC^*c*^		∼80	67, 68
INP1767 HQ		14.6 YopE^*e*^ EC_50_				12.5 MIC^*c*^			67
INP1855 HQ	∼60^*c*^	6.3 YopE^*e*^ EC_50_				3.13 MIC^*c*^		∼30	67, 69
INP0341 SAH^*g*^	∼80^*c*^					∼20^*c*^			68, 70
INP0400 SAH						∼20^*c*^			70
INP0403 SAH				∼100 SipAC^*b*^					64, 71
INP0007 SAH		∼50 YopE^*b*^		∼100 SipAC^*b*^					64, 72
C2, C4 SAH		∼20; ∼5 Yops^*b*^							73
ME0052 SAH		∼20 Yops^*b*^							74
INP0010 SAH C1 SAH		∼50 YopE^*b*^ ∼50 Yops^*b*^							75
ME0055 SAH (INP0031)					∼20 LEE genes^*e*^				76
RCZ12 SAH RCZ20 SAH					∼25 EspD^*b*^				77
INP0401 SAH 5277768 SAH							∼50 hrp^*e*^		78
Compound 3	13 ExoS^*b*^	6 YopE^*b*^							65
C20	∼60^*c*^	∼60 YopE^*f*^							79
Compound D	∼60 ExoU^*e*^	∼60 YopE^*b*^							80
Salicylideneanilide					15 EspB^*b*^				81
Piercidin A1, Mer-A 2026B		∼36; ∼9 YopM^*f*^							82
*N*-arylbenzylamines						∼50 IncA^*f*^			83
Baicalein flavonoid				3.6 SopE2^*b*^		0.5 mM^*c*^			84, 85
Licoflavonol				∼50 SipC^*b*^					86
Epigallocatechin gallate		∼16 μg/ml Yops^*b*^		∼12μg/ml Sips^*b*^	∼16 μg/ml EspB^*b*^				87
Sanguinarine chloride				∼5 SipA^*b*^^f^					88
Obovatol				19.8^*c*^					89
Thymol				∼0.2 mM SipA^*f*^					90
TTS29 thiazolidinone		∼380 Yops^*b*^	∼380 Ysps^*b*^	∼100 Sips^*b*^			∼380^*c*^		91
WEN05-03					∼100^*c*^				92
Fluorothiazinon (also CL-55)	∼20 μg/ml ExoT^*b*^ ExoY^*b*^			∼10 mg/kg^*c*^, ∼50 SipA^*a*^		∼25^*c*^			93 – 96
(-)-Hopeaphenol	∼50 ExoS^*b*^	3.3 YopD^*b*^				∼25^*c*^			97
Resveratrol oligomers							∼100 *hrpA*^*e*^		98
Paeonol				∼95 Sips^*b*^					99
Syringaldehyde				∼180 Sips^*b*^					100
Fusaric acid				53.5 SipC^*b*^					101
Cytosporone B				6.25 SipC^*b*^				NE^*d*^	102
Aurodox					0.5 μg/ml EspABCD^*b*^				103
W1227933, W1774182						25 IncA^*f*^			83
BCD03							67.3^*b*^		104
α-tocopherol	∼10^*c*^ ExoY^*f*^								105
Cinnamaldehyde				∼100^*c*^ SipAB^*e*^					106
Myricanol				41.34 SipC^*a*^					107
Myricetin				∼4 μg/ml SipA/B^*f*^					107
4-Hydroxybenozic acid							∼2.5 mM hrpA^*e*^		108
Vanillic acid							∼2.5 mM hrpA^*e*^		108
Epigallocatechin-3-gallate (EGCG)					1.8 EspF^*f*^				109
Tannic acid					0.65 EspF^*f*^				109
Sepantronium bromide (YM155)					∼2 SseK1/SseK2^*e*^, NleB^*e*^				110

a Species/T3SS family: PA, Pseudomonas aeruginosa; Ysc, Yersinia pseudotuberculosis Ysc; Ysa, Yersinia enterocolitica Ysa; SPI-1, Salmonella enterica Typhimurium SPI-1; SPI-II, Salmonella enterica Typhimurium SPI-II; EPEC/EHEC, enteropathogenic E. coli/enterohemorrhagic E. coli; PS, Pseudomonas syringae; Fla, flagella. Empty cell denotes activity not tested.

b IC_50_ (in μM, unless otherwise indicated) measured using the indicated organism/T3SS family/effector protein in a culture-based secretion assay. If IC_50_ data are not available, either the lowest known inhibitory concentration (indicated by “∼”), EC_50_ (half maximal effective concentration), or MIC is shown.

c IC_50_ (in μM) measured using cell-based infection assays.

d No effect observed.

e IC_50_ (in μM) measured using a biochemical assay (i.e., binding assay, enzymatic assay, qPCR).

f IC_50_ (in μM) measured using translocation assay.

g Phe, phenoxyacetamide; HQ, hydroxyquinoline; SAH, salicylidene acylhydrazides.

Secretion of protein substrates through the injectisome T3SS, the flagellar system, and the Tat system require the proton motive force (45–47). Although cyclic peptomers inhibited secretion from the injectisome T3SS, they did not inhibit the Tat system and only weakly inhibited flagellar substrate secretion, suggesting that the proton motive force is unaffected, as we previously suggested (22), and that the cyclic peptomers do not inhibit bacterial secretion in general. These results suggest that cyclic peptomers act as broad-spectrum, but specific, inhibitors of the injectisome T3SS.

The 4EpDN cyclic peptomer demonstrated efficacy against the T3SSs of P. aeruginosa, Y. pseudotuberculosis, Y. enterocolitica, Salmonella enterica Typhimurium, and Chlamydia trachomatis, with an IC_50_ in the range of 1 μM (for the Salmonella SPI-1 T3SS) to ∼16 μM (for the Y. pseudotuberculosis Ysa T3SS) (Table 2). Based on phylogenetic analysis of core T3SS proteins, T3SSs group into seven T3SS families, five of which contain T3SSs from human pathogens (48). 4EpDN has efficacy against T3SSs from at least three of these T3SS families, the Ysc (Ysc and Psc), Inv-Mxi-Spa (SPI-1 and Ysa), and *Chlamydiales*. Interestingly, the flagellar ATPase from Escherichia coli falls at the root of the phylogenetic tree (49), distinct from other T3SS families. As 4EpDN impacted secretion through the flagellar T3SS significantly less than through the injectisome T3SS in the same bacterial species and under the same culture and experimental conditions, we reason that the pathway targeted by cyclic peptomers is common to all injectisome T3SSs but absent from the flagellar system.

The T3SS is a complex system of ∼20 different proteins and is assembled in a hierarchical manner prior to secretion of effector proteins (35, 50). The T3SS of *Yersinia*, Pseudomonas, Salmonella, and Chlamydia share a number of orthologous basal body components that must be assembled before secretion can occur. However, 4EpDN only slightly reduced localization of the inner membrane ring protein YscD to the *Yersinia* cell envelope. But 4EpDN significantly inhibited the ability of the *Yersinia* T3SS needle to bind anti-YscF antibodies by approximately 2-fold. In contrast, T3SS effector secretion was inhibited 4-fold at the same concentration, suggesting that inhibition of T3SS assembly may not be the only mechanism by which 4EpDN blocks T3SS activity. Interestingly, it is possible that the 4EpDN cyclic peptomer is more effective at inhibiting secretion of large T3SS cargo (such as ExoU, ∼74 kDa) compared to smaller cargo (such as ExsE, ∼9 kDa). As the YscF needle subunit is also ∼9 kDa, this may explain why T3SS needle detection at the bacterial surface is not as robustly inhibited as ExoU secretion. It is possible that the 4EpDN cyclic peptomer interacts with the lumen of the T3SS needle, impacting large cargo secretion more than small cargo secretion. Alternatively, 4EpDN may disrupt normal assembly of the T3SS needle subunit, resulting in nonfunctional needles that cause dramatic effects on the secretion of effectors. It is also possible that both phenomena contribute to the observed effects.

4EpDN strongly inhibited Chlamydia from infecting HeLa cells during primary infection and subsequently completely prevented Chlamydia from infecting additional host cells. At the early stage of Chlamydia infection, T3SS plays major roles in invasion, EB to RB differentiation, and replication through presynthesized T3SS effectors and early and midcycle effectors (51). These effectors mediate nutrient acquisition and maintain the viability of the host cell. A decrease in inclusion number at the end of the midcycle (in primary infection) suggests inhibition of one or more of the above processes. At the late stage of infection (∼24 to 72 hpi), the RB to EB transition occurs, and late-cycle effectors are generated and packaged in progeny EB to prepare for the next infection cycle (51, 52). 4EpDN has a particularly strong effect on the secondary infection (assayed at 48 hpi), suggesting that the cyclic peptomer may inhibit secretion of presynthesized C. trachomatis effectors. This highlights the potential of cyclic peptomers to prevent the spread of Chlamydia infection. Chlamydia relies on its T3SS effector proteins to interact with host factors, such as the actin cytoskeleton, Golgi network, endoplasmic reticulum, and microtubule network, to mediate invasion and intracellular growth (51). It is possible that compounds that inhibit these host pathways could interfere with chlamydial growth (53–55). However, microscopic analysis of many cellular structures in HeLa cells in the presence of 4EpDN did not show any gross changes to the actin cytoskeleton, Golgi network, endoplasmic reticulum, or microtubule network at the concentration used in our Chlamydia infection (Fig. S8). C. trachomatis infection may cause infertility in female patients and eye damage, in addition to lung infections (21). Antibiotics, such as β-lactam antibiotics, are a common way to treat Chlamydia infection, but the chance of recurrence is high (56, 57). Current vaccine development efforts are under way, but multiple challenges remain (58). There is increased demand for drugs against Chlamydia due to antibiotic resistance (59). The strong efficacy of cyclic peptomers highlights their potential for development as an anti-chlamydial drug.

Overall, the cyclic peptomer 4EpDN specifically targets the injectisome T3SS of Gram-negative bacteria. As the T3SS is important to overcome host defense mechanisms, inhibition of this virulence mechanism may augment the function of the host immune system to clear infection. Thus, the cyclic peptomer has potential to be used as a prophylactic to prevent infections with T3SS-expressing pathogens or in combination with antibiotics to treat existing infections. The strong inhibitory effect of 4EpDN on C. trachomatis infection of human cells suggests the possibility of using this compound as a topical prophylactic against Chlamydia genital infection. Further pharmacokinetics studies will establish the stability of this and related compounds in the host, expanding the potential of the cyclic peptomers to be used as therapeutics against additional infections.

## MATERIALS AND METHODS

### Bacterial strains and culture conditions.

The bacterial strains and cell lines used in this study are listed in Table 3. All cultures were grown with shaking at 250 rpm unless otherwise noted. Y. pseudotuberculosis was grown in 2×YT (2× yeast extract and tryptone) at 26°C overnight. To induce the T3SS, the cultures were subcultured to an optical density at 600 nm (OD_600_) of 0.2 into low-calcium medium (2×YT with 20 mM sodium oxalate and 20 mM MgCl_2_). Y. enterocolitica was grown in brain heart infusion (BHI) medium at 26°C overnight. The Ysc T3SS in Y. enterocolitica was induced using low-calcium BHI (BHI with 20 mM sodium oxalate and 20 mM MgCl_2_). The Ysa T3SS was induced as described previously (60) using L medium (1% tryptone, 0.5% yeast extract) with 290 mM NaCl at 26°C. P. aeruginosa and S. enterica were grown in Luria-Bertani (LB) medium overnight at 37°C. For P. aeruginosa, the T3SS was induced using low-calcium medium (LB with 5 mM EGTA and 20 mM MgCl_2_). SPI-1 T3SS secretion was assessed after subculturing into fresh LB at 37°C unless noted otherwise. C. trachomatis serovar L2 (LGV 434) was propagated in HeLa 229 cells. C. trachomatis EBs were harvested from infected cells and purified using a Renografin step-gradient as previously described (61).

**TABLE 3:** Bacterial strains used in this study

Strain	Description	Reference/source
Y. pseudotuberculosis strains		
Wild type	Y. pseudotuberculosis IP2666	111
*tatB::Tn*; Bla	IP2666 Δ*YopHEMOJ* *tatB*::TnHimar1 insertion; carrying 30aa_sufI_::β-lactamase TEM1	22
Wild type; Bla	IP2666 carrying 30aa_sufI_::β-lactamase TEM1	This study
Pseudomonas aeruginosa strains		
Wild type	P. aeruginosa PA103	112
Δ*exoUT*	PA103 Δe*xoU*/Δe*xoT*	113
PAO1 efflux pump mutant	PAO1 Δ(mexAB-oprM) nfxB Δ(mexCD-oprJ) Δ(mexEF-oprN) Δ(mexJKL) Δ(mexXY) ΔopmH362::pGSV3-Spr -exoT′-aacC1::miniCTXexoS(E379A/E381A)-blaM	44
Yersinia enterocolitica		
Wild type	Y. enterocolitica 8081 serotype O:8	114
pYV40-EGFP-yscD	Y. enterocolitica serotype O9 strain E40 carrying EGFP-yscD	12
Salmonella enterica Typhimurium strains		
WT	S. enterica Typhimurium SL1344	115
Δ*fliC*	SL1344 Δ*fliC*	115
Escherichia coli		
E. coli DH5α	E. coli DH5α carrying 30aa_sufI_::β-lactamase TEM1	This study
Chlamydia trachomatis	C. trachomatis serovar L2	Joanne Engle

HeLa cells (ATCC) were cultured in Dulbecco’s modified Eagle’s medium (DMEM) with 10% fetal bovine serum (FBS). All cell lines were incubated at 37°C with 5% CO_2_.

### Bacterial viability assay.

Overnight cultures of WT P. aeruginosa (PAO1) or Y. pseudotuberculosis were back-diluted 1:40 in LB or in 2×YT, respectively, and grown for 1.5 h at 37°C (Pseudomonas) or 26°C (*Yersinia*). Then, 384-well plates were prepared with one-half final volume of medium, compounds, and 10% vol/vol resazurin-based alamarBlue high-sensitivity (HS) cell viability reagent (catalog number A50101; Invitrogen). After incubation, the cultures were centrifuged for 5 min at 14,800 rpm. Supernatant was removed, and the pellets were resuspended in medium. Cultures were then normalized to an OD_600_ of 0.0005 and added to the prepared 384-well plates. Plates were incubated in a plate reader (PerkinElmer Envision 2105) at 37°C (Pseudomonas) or room temperature (*Yersinia*), and fluorescence was measured every hour for 12 h.

### Preparation of bacteria for T3SS induction.

Visualization of secreted proteins was carried out as described previously (25). Briefly, Y. pseudotuberculosis, P. aeruginosa, or S. enterica was grown in T3SS-inducing medium (as described above) in the presence of cyclic peptomers or an equivalent volume of DMSO at 37°C for 2 h for Y. pseudotuberculosis Ysc T3SS, 3 h for P. aeruginosa, 4 h for S. enterica, or at 26°C for 6 h for the Y. enterocolitica Ysa T3SS. The cultures were normalized to bacterial density (OD_600_) and then centrifuged for 15 min at 14,800 rpm. The supernatants were transferred to new tubes and mixed with trichloroacetic acid (TCA) to a final volume of 10% by vortexing vigorously for 30 s. Samples were incubated on ice for 1 h and then spun down at 4°C for 15 min at 13,200 rpm. The supernatants were carefully removed, and pelleted proteins were washed with acetone and spun down at 4°C for 15 min at 13,200 rpm for a total of three washes. The pellet was then resuspended in final sample buffer (FSB) and 20% dithiothreitol (DTT) and boiled for 15 min prior to SDS-PAGE. Tween 20 was added to the bacterial culture at the same time as the compounds in S. enterica secretion assays at 0.003% (vol/vol).

### T3SS secretion cargo quantification.

Image Lab software (Bio-Rad) was used to quantify T3SS cargo protein bands relative to those of DMSO-treated controls. The WT Y. pseudotuberculosis YopE, P. aeruginosa ExoU, or S. enterica SipA, SipC, FliC, and FliD bands in DMSO control samples were set to 1.00. To evaluate type III secretion of ExsE in P. aeruginosa, Western blotting against T3SS cargo was carried out using a polyvinylidene difluoride (PVDF) membrane (Millipore). Prior to blocking, membranes were incubated with acetone at 4°C for 30 min with gentle shaking. Membranes were then moved to Tris-buffered saline with 0.1% Tween 20 (TBST) and heated to 50°C for 30 min. Blots were blocked in 2.5% nonfat milk for 1 h at room temperature and incubated with anti-ExsE at 4°C overnight with gentle shaking. Blots were washed three times for 5 min each in TBST. Horseradish peroxidase conjugated secondary antibody was then incubated for 1 h at room temperature. Signals were detected with a luminol kit (catalog number sc-2048; Santa Cruz Biotechnology, Inc.) after washing. ExsE, BSA, RpoA, and SipC were visualized with anti-ExsE antibody (courtesy of Timothy Yahr) (20% tris-tricine gel), anti-BSA (catalog number 2A3E6; Santa Cruz Biotechnology, Inc.), anti-RpoA (gift from Melanie Marketon) (7.5% tris-glycine gel), and anti-SipC (catalog number ABIN335178; Antibodies-online, Inc.) (10% tris-glycine gel), respectively.

### YscD visualization assay.

Y. enterocolitica expressing YscD-EGFP was cultured overnight in BHI supplemented with nalidixic acid (35 μg/ml) and diaminopimelic acid (80 μg/ml) at 26°C with shaking (12), followed by subculturing into low-calcium BHI medium (20 mM NaOX, 20 mM MgCl_2_, 0.4% glycerol) with nalidixic acid and diaminopimelic acid to an OD_600_ of 0.2 for 1.5 h. Compounds or an equivalent volume of DMSO was added prior to inducing the T3SS. After 3 h at 37°C with shaking, cells were pelleted and resuspended in M9 medium supplemented with diaminopimelic acid, and compounds, spotted onto a 0.1% agarose pad supplemented with diaminopimelic acid and compounds and imaged live at ×63/1.4 oil magnification using a Zeiss AxioImager widefield microscope. Analysis of YscD puncta was carried out in Imaris 8 using spot tracking analysis with the same arbitrary threshold to call bacterial cells and puncta for all samples. Samples were prepared blinded, and each sample was imaged at the same 10 selected views covering the entire sample.

### YscF needle staining assay.

Y. pseudotuberculosis was cultured, and the T3SS was induced as described above. Compounds or an equivalent volume of DMSO was added prior to inducing the T3SS. Cells were fixed by mixing 500 μl of bacterial culture with 800 μl 4% paraformaldehyde (PFA), 1 μl of 25% gluteraldehyde, and 40 μl of 0.5 M sodium phosphate. The mixture was gently inverted repeatedly to mix and left at room temperature for 15 min before being moved to ice for an additional 30 min. Cells were pelleted gently at 5,000 × *g* for 3 min at 4°C. Pellets were gently resuspended and washed with ice-cold phosphate-buffered saline (PBS) for a total of three times. The final pellet was resuspended in GTE buffer (50 mM glucose, 25 mM Tris base, pH 8.0, and 10 mM EDTA). Cells were spotted and spread onto a coverslip and allowed to partially dry. Then, 1% bovine serum albumin (BSA) in phosphate-buffered saline with 0.1% Tween 20 (PBST) was added to the coverslips and left overnight, gently shaking at 4°C. The BSA/PBST block was removed, and anti-YscF antibody was diluted in 1% BSA/PBST and added to the coverslips. Coverslips were incubated with anti-YscF antibody for 4 h at 4°C while gently shaking. Anti-YscF antibody was removed, and coverslips were washed with PBST three times for 5 min each. mCherry-tagged secondary antibody was diluted in 1% BSA/PBST, added to the coverslips, and incubated in the dark at 4°C for 2 h while gently shaking. Secondary antibody was removed, and coverslips were washed with PBST three times for 5 min each. Hoescht 33342 (Thermo Fisher Scientific) was diluted in PBST, added to the coverslips, and incubated in the dark for 20 min at room temperature while gently shaking. Hoescht stain was washed away by washing the coverslips 3 times with PBST for 5 min each. Coverslips were mounted to slides using ProLong Gold (Life Technologies). Slides were imaged at ×63/1.4 oil magnification using a Zeiss AxioImager widefield microscope. Analysis of YscF puncta was carried out in Imaris 8 using spot tracking analysis with the same arbitrary threshold to call bacterial cells and puncta for all samples. Analysis was performed in batches for all conditions within a replicate.

### mRNA quantification by qPCR.

Overnight P. aeruginosa (PA103 or PAO1) cultures were subcultured and shifted to T3SS-inducing conditions (see above) in the presence of 60 μM 1EpDN, 60 μM 1EpDN 2Sar, or 50 μM MBX1641. Samples were taken after 3 h of induction. Overnight Salmonella cultures were subcultured into fresh LB with 0.3 M NaCl at 37°C in the presence of 9 μM 4EpDN, 4EpDN 2Sar, or equivalent DMSO. Samples were taken after 2 h and 4 h of induction. Samples were stored in RNAprotect reagent (Qiagen) and processed within a week. Total RNA was isolated using an RNAeasy kit (Qiagen) according to the manufacturer’s instructions, followed by two rounds of Turbo DNase (Thermo Fisher scientific) treatment. A total of 2 μg of RNA was used to make cDNA, and quantitative PCRs (qPCRs) were run with SYBR green PCR master mix (Applied Biosystems). DNA helicase (*dnaB*) and 16S rRNA genes were used as a reference for P. aeruginosa and Salmonella samples, respectively. Two to three technical replicates were averaged for each sample. The primers used are listed in Table S2. Results were analyzed using Bio-Rad CFX software.

### Tat assay.

To make Tat targeting constructs, plasmid pMMB67EH (ATCC 37622) was digested with KpnI. TEM1 of β-lactamase was PCR-amplified from *yopH*-Bla (courtesy of Melanie Marketon) using primers oHL210 and oHL217 (Table S2), and *sufI* signal peptide DNA was PCR-amplified from genomic DNA of Y. pseudotuberculosis with primers oHL218 and oHL219 (Table S2). The digested pMMB67EH, TEM1, and *sufI* signal peptide DNA were assembled into a plasmid (*sufI*-Bla) using Gibson assembly.

WT *Yersinia* or *tatB*::*Tn* carrying sufI-Bla was grown in 2×YT supplemented with 15 μg/ml gentamicin at 26°C with shaking. Overnight cultures were subcultured to an OD_600_ of 0.1 and grown for 1.5 h at 26°C with shaking. Then, 5 mM IPTG was added to the culture for 0.5 h to allow for expression and translocation of SufI-Bla. Penicillin G (25 μg/ml) was added to the cultures. Cultures were then treated with cyclic peptomers or DMSO, and the OD_600_ was measured every hour up to 8 h.

### Chlamydia infection and imaging.

**Primary infections.** HeLa cell monolayers were infected with C. trachomatis serovar L2 at a multiplicity of infection (MOI) of 1.0 in the presence of one of the following compounds at 9 μM: DMSO, 4EpDN, or 4EpDN 2Sar. Cells were incubated for 24 h in the presence of the above-listed compounds at 37°C and then fixed with 4% paraformaldehyde (PFA). Cells were stained for IncA (Chlamydia inclusion membrane marker), DNA (with DAPI), and MOMP (Chlamydia major outer membrane protein) simultaneously. This method of staining allows visualization of the inclusion independent of MOMP synthesis or transport. The percentage of cells infected (i.e., stained positively for the listed Chlamydia markers) in the presence of the compounds was quantified using confocal microscopy. Quantifications of inclusion take into account both DAPI staining and indirect immunofluoresence with an antibody to MOMP. Ten randomly selected fields of view were measured per experiment. The data represent three biological replicates.

**Secondary infections.** HeLa cell monolayers were infected with C. trachomatis serovar L2 at an MOI of 1.0 in the presence of one of the following compounds at 9 μM: DMSO, 4EpDN, or 4EpDN 2Sar. Cells were incubated for 48 h in the presence of the above-listed compounds at 37°C. Infected cells were then lysed, and the lysate was applied to fresh HeLa monolayers to enumerate infectious particles. These secondary infections were fixed in 4% PFA at 24 hpi and were stained against MOMP and DNA. The infectious units per ml (IFU/ml) were calculated by averaging the number of infected cells in each of 10 randomly selected fields of view at ×40 magnification on a confocal microscope and multiplying this by the appropriate dilution and area factors. The data represent four biological replicates.

### Cytological profiling (CP).

Briefly, HeLa cells were cultured and seeded into 384-well plates at 2,500 cells/well. After 48 h, compounds were added using a Janus MDT robot (PerkinElmer). Two stain sets were used—stain set 1, Hoechst, EdUrhodamine, anti-phosphohistone H3, and GM130; stain set 2, Hoechst, fluorescein isothiocyanate (FITC)-alpha tubulin, rhodamine-phalloidin, and calnexin. For stain set 1, cells were incubated with 20 μM EdUrhodamine for 1 h prior to fixing in 4% formaldehyde solution in PBS for 20 min. Cells were then washed with PBS and permeabilized with 0.5% Triton X-100 in PBS for 10 min before blocking with 2% BSA in PBS solution for at least 1 h. Following this, cells were incubated with primary antibodies overnight at 4°C. The following day, excess primary antibody was washed off with PBS and Alexa-488, and Alexa-647 secondary antibodies and Hoechst solution were incubated for 1 h. Plates were washed with PBS and preserved with 0.1% sodium azide in PBS solution prior to imaging. For stain set 2, cells were fixed with a 4% formaldehyde solution in PBS for 20 min. Cells were then washed with PBS and permeabilized with 0.5% Triton X-100 in PBS for 10 min before blocking with 2% BSA in PBS solution for at least 1 h. Following this, cells were incubated with primary antibodies overnight at 4°C. After blocking, the cells were washed and then incubated with FITC conjugated anti-alpha tubulin antibody and rhodamine-phalloidin overnight at 4°C. The following day, the cells were washed and then incubated with secondary Alexa-647 and Hoechst stain for 1 h. Plates were washed with PBS and preserved with 0.1% sodium azide in PBS solution prior to imaging.

Two images per well were captured with an ImageXpress Micro XLS automated epiflourescent microscope (Molecular Devices). Images were then processed as described in reference 62. Briefly, initial image processing was performed using MetaXpress image analysis software, using built-in morphometry metrics, the multiwavelength cell scoring, transfluor, and micronuclei modules. Custom-written scripts were used to compare the treated samples with the DMSO control wells and then to convert each feature to a histogram difference (HD) score. This produced a 452-feature vector CP fingerprint. Compound treatment wells were labeled as dead if the cell count for the treatment well was <10% of the median cell count in the treatment plate. In addition to the CP fingerprint, feature cell counts (nuclei, EdU S-phase, and phospho-histone H3) were used to determine the effects of compounds on HeLa cell replication.

### Synthesis of cyclic peptomers.

**Cyclic peptide synthesis.** Peptides were synthesized using standard Fmoc solid-phase peptide synthesis, utilizing the submonomer approach for peptoid synthesis (63), either at room temperature or with microwave assistance. Cyclization was done in solution at a high dilution. Fmoc-Xaa (10 mmol) was added to a flame-dried round-bottomed flask and dried in a vacuum desiccator with phosphorous pentoxide overnight. Then, 50 ml of dry dichloromethane (DCM) was cannula-transferred into the flask, followed by 2.5 ml of *N*,*N*-diisopropylethylamine (DIPEA) transferred via syringe. After sonication for 10 min, 5 g of 2-chlorotrityl resin was added under a stream of nitrogen and allowed to shake for 4 h. The resin was capped with a 15-ml solution of 1:2:17 methanol (MeOH):DIPEA:dimethylformamide (DMF) (3 times for 15 min each). The resin was washed with DMF (3 times with 15 ml each) followed by DCM (3 times with 15 ml each). The loading value was calculated by determining the mass increase of dried, loaded resin.

### Amino acid coupling at room temperature.

Four equivalents (eq) of Fmoc-Xaa, 8 eq of DIPEA, and 4 eq of 1-[Bis(dimethylamino)methylene]-1H-1,2,3-triazolo[4,5-b]pyridinium 3-oxide hexafluorophosphate, hexafluorophosphate azabenzotriazole tetramethyl uronium (HATU) were added to the resin in DMF. The reaction mixture was agitated via shaking for 45 min and then drained. The resin was washed with DMF (3 times with 3 ml each) and DCM (3 times with 3 ml each). The reaction was monitored by liquid chromatography-mass spectrometry (LC-MS) and repeated until the starting material was no longer observed. For microwave conditions, a solution of 4 eq of Fmoc-Xaa, 4 eq of HATU, and 6 eq of DIPEA in DMF was allowed to prereact for 5 min. This solution was added to the deprotected peptide on-resin and allowed to react for 10 min at 50°C under microwave heating. The solution was drained, and the resin was washed with DMF (3 times with 3 ml each) and DCM (3 times with 3 ml each). The reaction was monitored by LC-MS and repeated until the starting material was no longer observed.

### Coupling of BrAcOH at room temperature.

A solution of 10 eq of bromoacetic acid (BrAcOH) and 5 eq of *N*,*N*-diisopropylcarbodiimide (DIC) in DMF was allowed to prereact for 10 min. This solution was added to the deprotected peptide on-resin. The reaction mixture was agitated via shaking for 45 min and then drained. The resin was washed with DMF (3 times with 3 ml each) and DCM (3 times with 3 ml each). The reaction was monitored by LC-MS and repeated until the starting material was no longer observed. The reaction was monitored by LC-MS and repeated until the starting material was no longer observed.

### Peptoid side chain addition.

A solution of 5 eq of the desired amine was prepared in a minimum volume of DMF. The resin containing the BrAc-peptide was swollen with DCM for 5 min prior to reaction. The amine was added, and the reaction mixture was agitated via shaking for 3 to 20 h. The solution was drained, and the resin was washed with DMF (3 times with 3 ml each) and DCM (3 times with 3 ml each). The reaction was monitored by LC-MS and repeated until the starting material was no longer observed.

### Removal of the N-Fmoc protection group at room temperature.

A solution of 2% piperidine and 2% 1,8-diazabicyclo[5.4.0]undec-7-ene (DBU) in DMF was added to the resin. The reaction mixture was agitated via shaking for 20 min and then drained. The resin was washed with DMF (3 times with 3 ml each) and DCM (3 times with 3 ml each). For microwave conditions, a solution of 2% piperidine and 2% DBU in DMF was added to the resin. The reaction mixture was allowed to react for 5 min at 50°C under microwave heating and then drained. The resin was washed with DMF (3 times with 3 ml each) and DCM (3 times with 3 ml each).

### Peptide cleavage.

Complete linear peptides were cleaved off the resin in 5 resin volumes of 2.5% trifluoroacetic acid (TFA) in DCM for 4 min, three times, with a 5-resin-volume DCM wash between steps. Solvent was removed under N2, followed by dissolution in acetone or DCM and evaporation under reduced pressure. Residual TFA was removed *in vacuo* overnight.

### Cyclization with COMU.

Linear peptides were dissolved in 20 ml of dry acetonitrile (ACN) with 4 eq of DIPEA and added dropwise (final concentration, 1 mg crude peptide per ml) to a solution of 1:1 tetrahydrofuran (THF)-ACN containing 2 eq of (1-cyano-2 ethoxy-2 oxoethylidenaminooxy) dimethylamino-morpholinocarbenium hexafluorophosphate (COMU). Reaction mixtures were stirred for 0.5 to 24 h, until complete cyclization was achieved as monitored by LC-MS. The reaction mixture was reduced *in vacuo* for purification via high pressure liquid chromatography (HPLC).

### Purification of peptides.

COMU by-products were removed after solution-phase cyclization on a Biotage Isolera Prime system equipped with a SNAP Ultra-C18 30-g column eluting with H_2_O-acetonitrile modified with 0.1% TFA. The mass spectra of all peptides are shown in Fig. S9 in the supplemental material.

### Proton NMR of peptides.

Peptides were analyzed through nuclear magnetic resonance (NMR) spectroscopy measured in ppm and were obtained on a 500-MHz spectrometer using CDCl_3_ (δ = 7.26) as an internal standard for ^1^H-NMR. Identity of compounds for SAR study was confirmed by LCMS and ^1^H-NMR (Fig. S9).

### Kinetic solubility.

A 15 mM stock of the compounds in DMSO was prepared, and 125 μl of M9 and DMEM (no antibiotics) was dispensed into a 96-well v-bottom plate. Then, 1 ml of 15 mM stock compound was added to make a solution of 120 μM final concentration with 0.8% DMSO. The solution was shaken at 37°C for ∼2 h. The solution was passed over a 0.7- μM glass fiber filter. Then, the solution was diluted 1:4 in acetonitrile to crash out any proteins. The solution was centrifuged at 500 × *g* for 10 min. Avoiding the pellet, 10 μl of supernatant was added to a fresh plate with 90 μl of acetonitrile. The final dilution is 40 times lower. Next, 10 μl of 40× dilution of solution was injected on the Orbi-trap. A 1 μM standard was used for the ratiometric comparison, and the assay was done in triplicate.

### Cyclic peptide manipulation.

Stock peptides were stored at 15 mM at −70°C. All treatment and control pairs, in all assays, had the same DMSO volumes. The compounds were prediluted in DMSO prior to experiments for lower concentrations when they were performed in conjunction with higher concentration treatment to maintain the same volume of DMSO.

### Statistical analysis.

Prism 9 (GraphPad Software, La Jolla, CA, USA) was used to calculate the mean, standard error of the mean, median, standard error of median, and one-way analysis of variance (ANOVA) values shown.
