# Supplementary material for: Developing Cyclic Peptomers as Broad-Spectrum Type III Secretion System Inhibitors in Gram-Negative Bacteria
Source: Antimicrob Agents Chemother. 2021 Jun 17;65(7):e01690-20. doi: 10.1128/AAC.01690-20 (PMC8373237; doi:10.1128/AAC.01690-20)
Supplement: Supplemental file 1 — Supplemental material. Download AAC.01690-20-s0001.pdf, PDF file, 13.1 MB [file aac.01690-20-s0001.pdf]

Table S1: Solubility of cyclic peptomers

	Solubility (μM)			IC_{50}
Compounds	PBS	DMEM	M9	
1EpDN 3Ala	180	ND[a]	ND[a]	>1000*
1EpDN 4Ala	75	ND[a]	ND[a]	>1000*
1EpDN 5Ala	127	ND[a]	ND[a]	>1000*
1EpDN 6Ala	125	ND[a]	ND[a]	>1000*
1EpDN	14-17	ND[a]	ND[a]	8.2
2EpDN	12	ND[a]	ND[a]	15.3
3EpDN	18	ND[a]	ND[a]	16
4EpDN	13	12	10	3.9
5EpDN	13	ND[a]	ND[a]	6.3
6EpDN	1	ND[a]	ND[a]	>1000*
7EpDN	26	ND[a]	ND[a]	6.7
8EpDN	1	ND[a]	ND[a]	41.5
9EpDN	10	ND[a]	ND[a]	12.8
4EpDN 1Sar	ND[a]	6	4	19
4EpDN 2Sar	ND[a]	61	61	~139.5

* >1000 indicates IC_{50} could not be calculated

[a]: Not Determined (ND)

Table S2. Primers used in this study

Name	Sequence	Description
oHL364	TACTGGAAACGGTGGCTAATAC	16S Forward for *Salmonella* qPCR
oHL365	TACCTCACCAACAAGCTAATCC	16S Reverse for *Salmonella* qPCR
oHL362	GCCAACGACGGTGAAACTA	fliC Forward for *Salmonella* qPCR
oHL363	GCCGTATCGCTGACCTTATATT	fliC Reverse for *Salmonella* qPCR
oHL346	ACGACTCATACATTGGCGATAC	hilA Forward for *Salmonella* qPCR
oHL347	CTGCGATAATCCCTTCACGATAG	hilA Reverse for *Salmonella* qPCR
oHL348	GGCGCTCTCTATGCACTTATC	hilD Forward for *Salmonella* qPCR
oHL349	GCAGGAAAGTCAGGCGTATAG	hilD Reverse for *Salmonella* qPCR
oHL350	GCAGCAAATTATTACGCCTTCTC	invF Forward for *Salmonella* qPCR
oHL351	CTGGTTGACTGAGCGAGTAAAT	invF Reverse for *Salmonella* qPCR
oHL352	ATGCGTTGTCCGGTAGTATTT	sipC Forward for *Salmonella* qPCR
oHL353	TTAAGCGCGCCTCTTTCA	sipC Reverse for *Salmonella* qPCR
oHL354	TCTTGTTATGCAGGAGGTGATG	sipA Forward for *Salmonella* qPCR
oHL355	GTCAACAAGGTGCGTAAGATTG	sipA Reverse for *Salmonella* qPCR
BQ89	CATGACCATCGCCTGATCTT	dnaB Forward for *P. aeruginosa* qPCR
BQ90	GTTGTCCTTCCTTCTCCAACT	dnaB Reverse for *P. aeruginosa* qPCR

oHL258	ATGCGGTAATGGACAAGGTC	exoT Forward for *P. aeruginosa* qPCR
oHL259	ACTCGCCGTTGGTATAGAGA	exoT Reverse for *P. aeruginosa* qPCR
oHL282	CCGGCAGATGTCCATTTC	DsbA Forward for P. *aeruginosa* qPCR
oHL283	CTCGACACCCATGCTTTC	DsbA Reverse for *P. aeruginosa* qPCR
BQ91	CTCTACACCGGCATTCACTAC	exoS Forward for *P. aeruginosa* qPCR
BQ92	CATACCTTGGTCGATCAGCTT	exoS Reverse for *P. aeruginosa* qPCR
oHL210	agcgaattcgagctcggtaccATGTCACTCAGTCGTCGC	Forward primer for 33 amino acid of *sufI* N terminus
oHL217	tttctgggtgAGGTTGCTGAGTACTACTAG	Reverse primer for 33 amino acid of *sufI* N terminus
oHL218	tcagcaacctCACCCAGAAACGCTGGTG	Forward primer for β-lactamase gene
oHL219	tctagaggatccccgggtaccTTACCAATGCTTAATCAGTGAGG	Reverse primer for β-lactamase gene

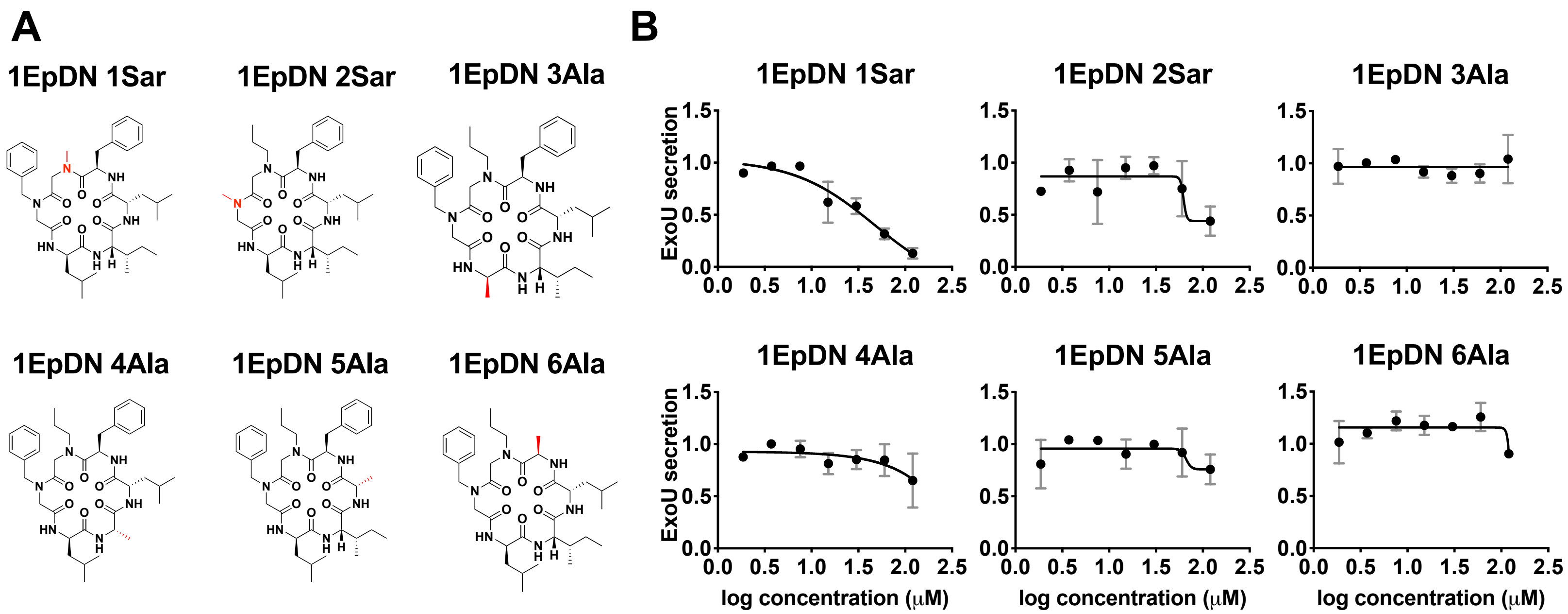
A
1EpDN 1Sar
1EpDN 2Sar
1EpDN 3Ala
1EpDN 4Ala
1EpDN 5Ala
1EpDN 6Ala
B
1EpDN 1Sar
1EpDN 2Sar
1EpDN 3Ala
1EpDN 4Ala
1EpDN 5Ala
1EpDN 6Ala
ExoU secretion
1.5
1.0
0.5
0.0
0.0 0.5 1.0 1.5 2.0 2.5
log concentration (μM)

Figure S1: 1EpDN alanine/Sarcosine scan suggests peptoid sidechains are important for biological activity. (A) Structures of 1EpDN alanine derivatives. D-form of side chain is shown in red. **(B)** WT *P. aeruginosa* PA103 was grown under T3SS-inducing conditions with increasing concentrations of cyclic peptomers. Secretion of T3SS cargo into the culture supernatant was assessed by precipitating secreted proteins and visualizing them with Coomassie blue. ExoU band intensities were quantified and normalized to that of the DMSO control. The results are from at least two independent experiments. Error bars are standard errors of the mean. Nonlinear curve fitting is shown to depict the trend of inhibition.

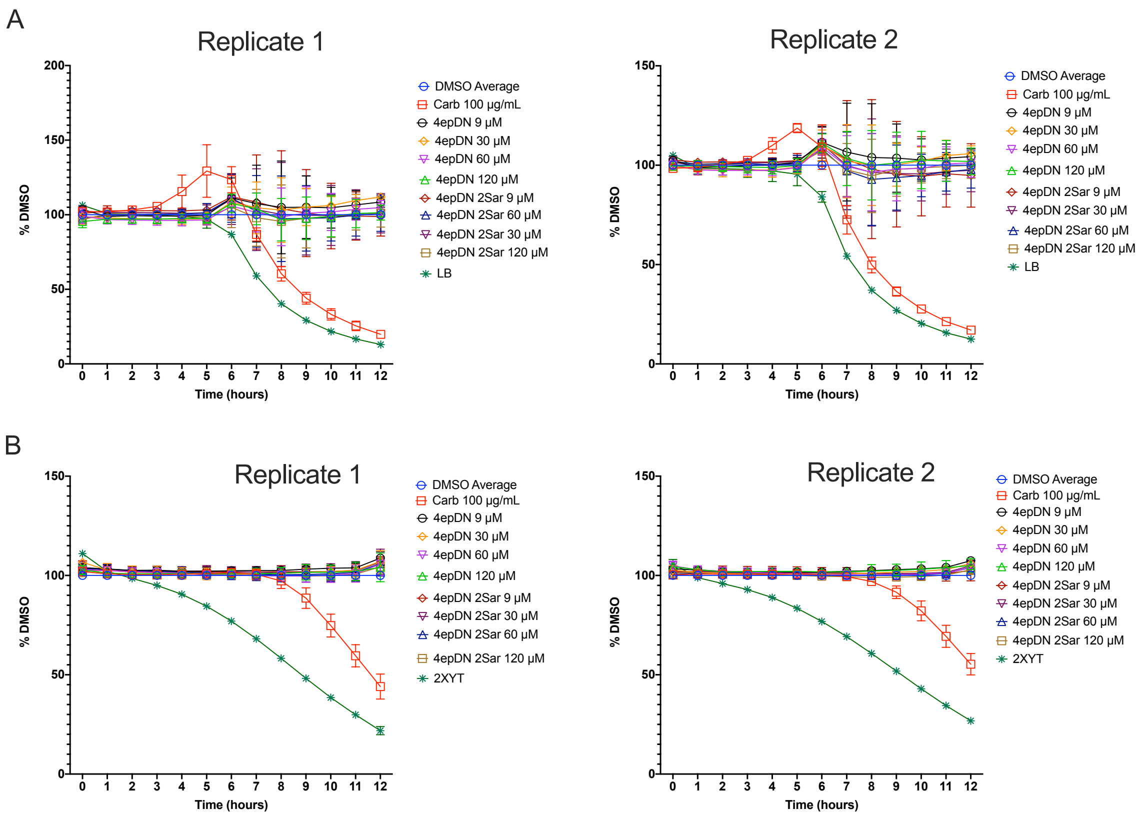

Figure S2: Cyclic peptomers do not affect bacterial viability. *P. aeruginosa* PA01 (A) or *Y. pseudotuberculosis* (B) was grown in LB or 2xYT medium at 37˚C or 26˚C in the presence of cyclic peptomers at 9 µM, 30 µM, 60 µM and 120 µM, equivalent volume of DMSO or Carbenicillin (100 µg/mL). Resazurin-based alamarBlue HS cell viability reagent was used to monitor metabolic activity of the bacteria over a period of 12 hrs. Fluorescence of treatment over control was plotted over time. Media only served as a control for bacterial growth. The experiment was carried out in two biological replicates with three technical replicate each.

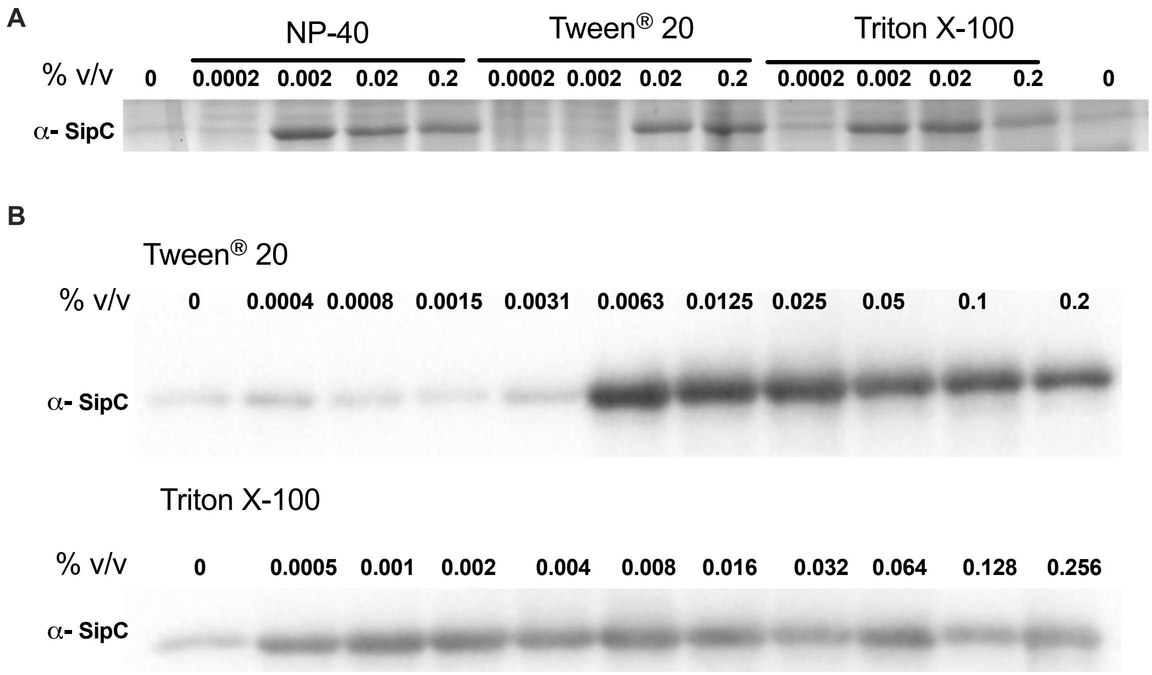

Figure S3: Secretion of *Salmonella* T3SS substrate in the presence of non-ionic detergents. Detergents without T3SS inhibitors were used to determine the highest concentration of detergent that can be added to the cultures without causing an observable effect on the T3SS**. (A)** *Salmonella enterica* Typhimurium was grown in LB with increasing concentrations of NP-40, Tween® 20, or Triton X-100. Secretion of SPI-1 T3SS effector SipC into the culture supernatant was assessed by precipitating secreted proteins and visualizing them with Coomassie blue. **(B)** Secretion of SipC in the presence of increasing concentrations of Tween® 20 or Triton X-100 was detected by Western Blot.

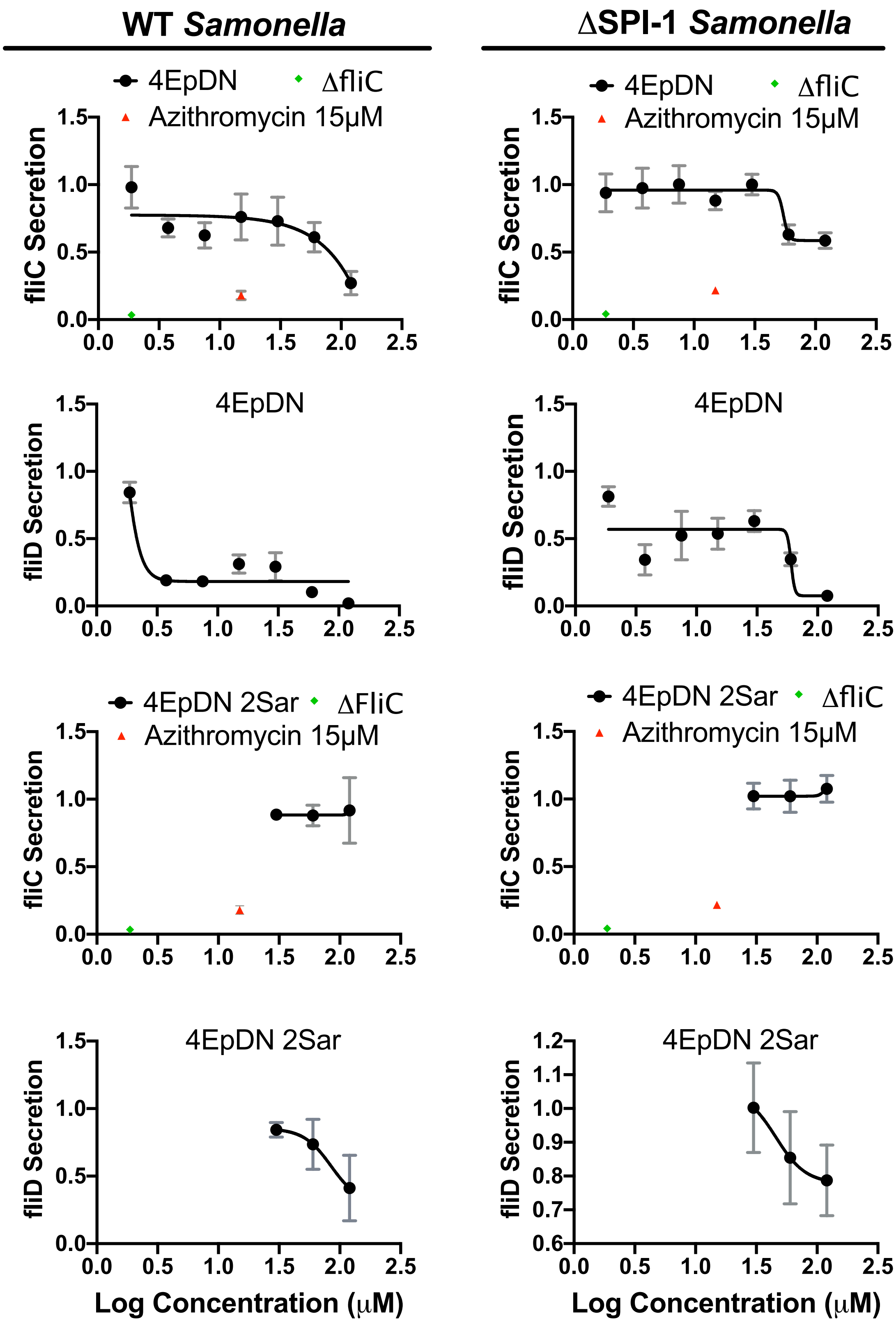
WT Samonella
ΔSPI-1 Samonella
4EpDN
ΔfliC
Azithromycin 15μM
fliC Secretion
fliD Secretion
4EpDN 2Sar
ΔFliC
Log Concentration (μM)

Figure S4: Cyclic peptomers do not affect secretion of flagellar proteins. *Salmonella enterica* Typhimurium was grown in LB with increasing concentrations of cyclic peptomers. Secretion of flagellar structural proteins FliC and FliD were assessed by precipitating the secreted proteins and visualizing them with Coomassie blue. A Δ*fliC* mutant and azithromycin (1), which inhibits flagellin secretion, were used as controls. The SPI-1 mutant and WT *Salmonella* were both tested, as flagella substrates can be secreted through both flagellar and SPI-1 T3SS systems. The results are from at least two independent experiments. Error bars are standard errors of the mean.

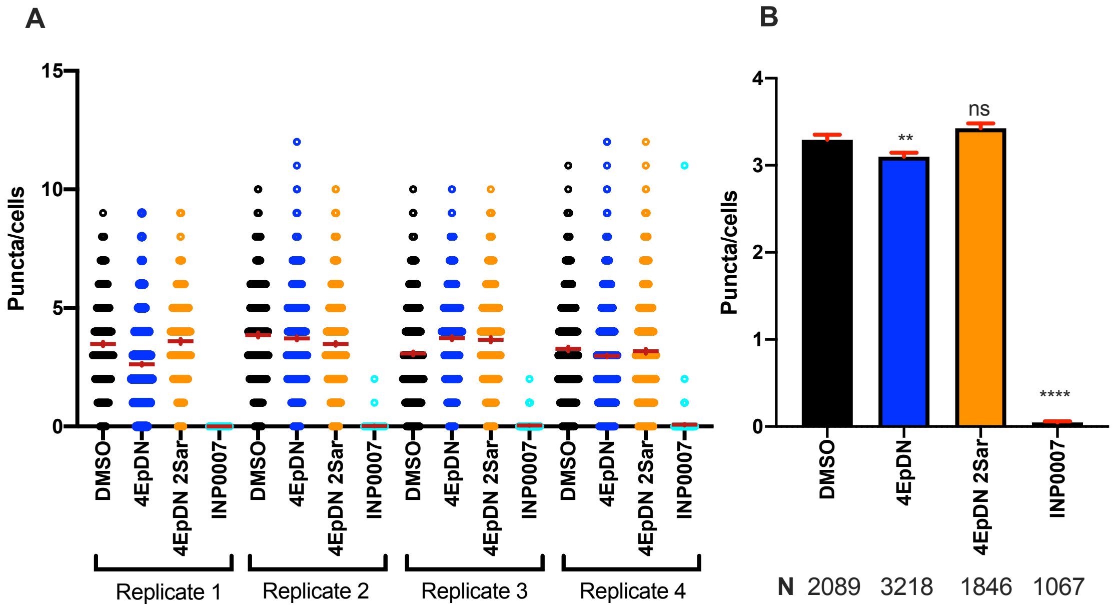

Figure S5: The cyclic peptomer 4EpDN effect on localization of the *Yersinia* T3SS basal body component YscD. *Y. enterocolitica* expressing YscD-EGFP was grown under T3SS inducing condition (low Ca^{2+}) in the presence of 9 µM cyclic peptomers, 50 µM INP0007, or DMSO. **(A)** Scatter plot of YscD puncta/cell for four replicates. Mean ±SEM was shown in red. Width of distribution of points is proportional to the number of data points at the Y value. **(B)** Mean number of puncta/cell after treatment for all replicates combined ±SEM. Data represents four independent experiments. Non-parametric Kruskal-Wallis test with Dunn's multiple-comparison test was used. ****, $P < 0.0001$; **, $P < 0.005$; ns: not significant.

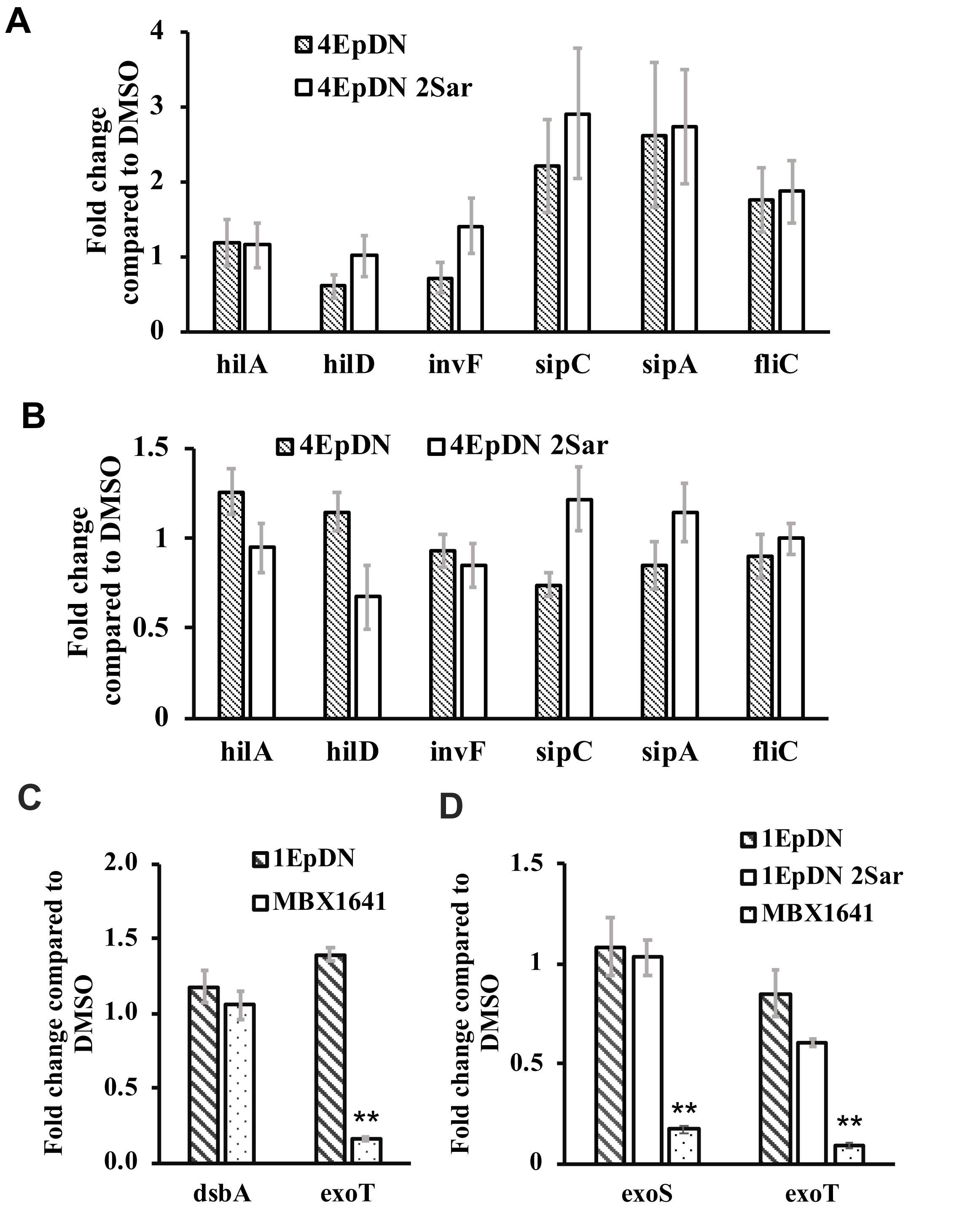

Figure S6: Cyclic peptomers do not affect transcription of T3SS genes in *Salmonella* and *P. aeruginosa*. *Salmonella enterica* Typhimurium was grown in LB with 300 mM NaCl in the presence of 9 μM cyclic peptomers or DMSO. Samples were taken 2 hrs (**A**)

and 4 hrs (**B**) after addition of compounds at 37°C and expression of flagellar (*fliC*) and injectisome T3SS (*hilA, hilD, invF, sipC, sipA*) genes were assessed using qPCR. *P. aeruginosa* PA103 **(C)** or PA01 **(D)** was grown in low calcium media in the presence of 60M cyclic peptomers or DMSO. Samples were collected 3 hrs after induction for qPCR analysis. The phenoxyacetamide MBX1641 (2), a known T3SS inhibitor predicted to inhibit type III secretion by binding to the T3SS needle subunit (3), was used as a control. Data are from two to three replicates, analyzed by one-way ANOVA with Dunnett's multiple-comparison test. **, $P < 0.01$.

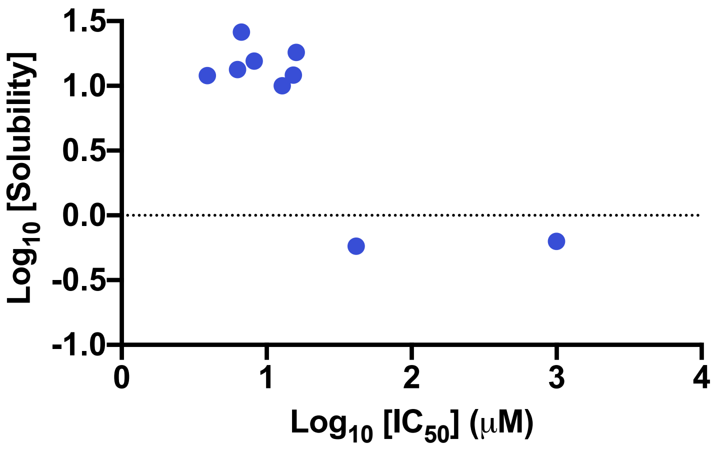

Figure S7: **Relationship between solubility and activity of cyclic peptomers**. IC_{50} of stereoisomers and their solubility (table S1) were plotted on a log_{10} scale. Average solubility was used when the solubility was measured in different conditions.

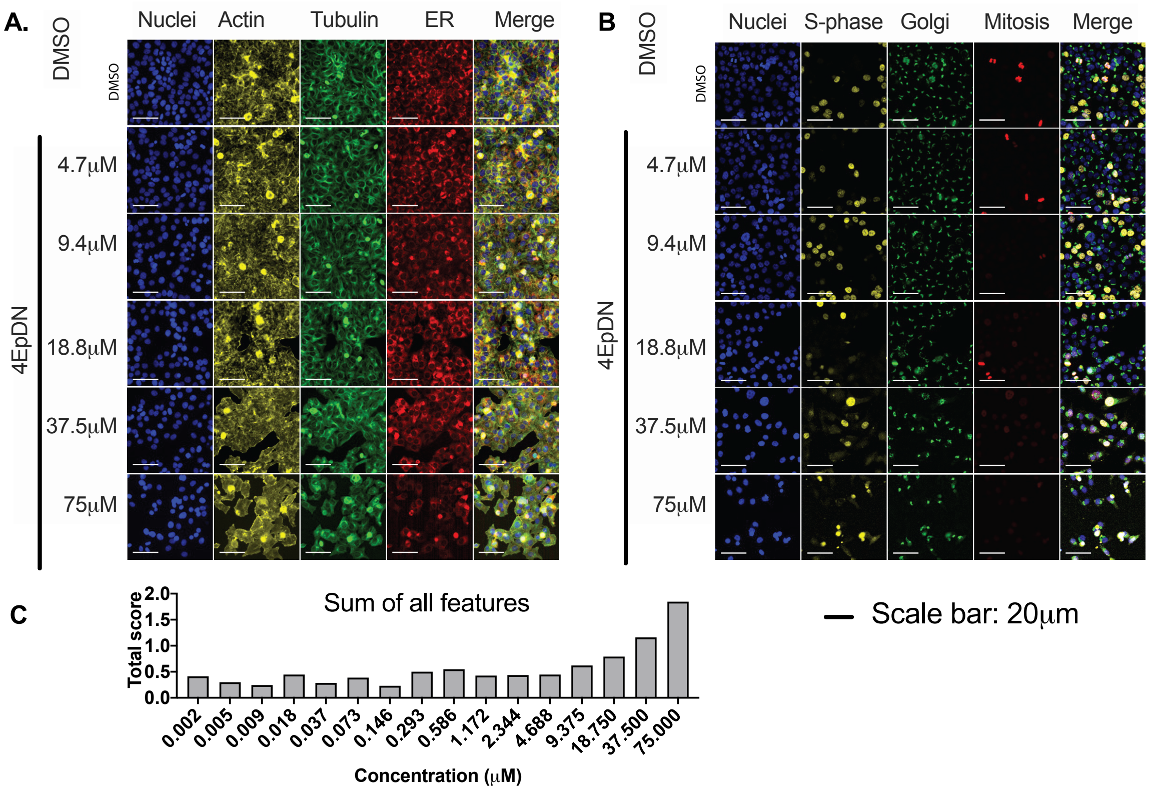

Figure S8: Effect of cyclic peptomers on HeLa cells. HeLa cells were incubated with compounds for 48 hrs. Cells were then stained with: **(A)** Stain Set 1: Hoechst, FITC-alpha tubulin, rhodamine-phalloidin (actin), and Calnexin (ER induced protein); or **(B)** Stain set 2: Hoechst, EdU-rhodamine (S-phase detection), anti-Phosphohistone H3 (mitosis marker), and GM130 (Golgi matrix protein). Representative images of cells treated with different concentrations of 4EpDN or DMSO are shown. **(C)** Quantification of all cell features for 4EpDN-treated cells. The total CP score is the square root of sum of square of the difference between treatment and DMSO for all measured features.

Figure S9: Characterization of cyclic peptomers. Drawn structures, SMILE structures, molecular weight, LCMS Spectra, and ^{1}H-NMR Spectra are shown.

1EpDN

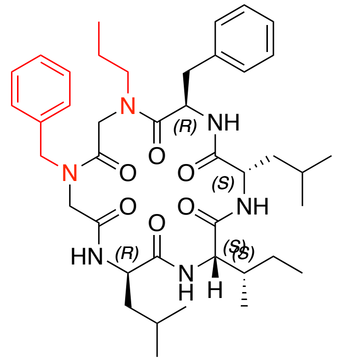

Exact Mass: 732.46
Molecular Weight: 732.97

SMILE structure:
O=C([C@H](CC(C)C)NC([C@@]([C@@H](C)CC)([H])N1)=O)N[C@@H](C(N(CC(N(CC2=CC=CC=C2)CC(N[C@H](CC(C)C)C1=O)=O)=O)CCC)=O)CC3=CC=CC=C3

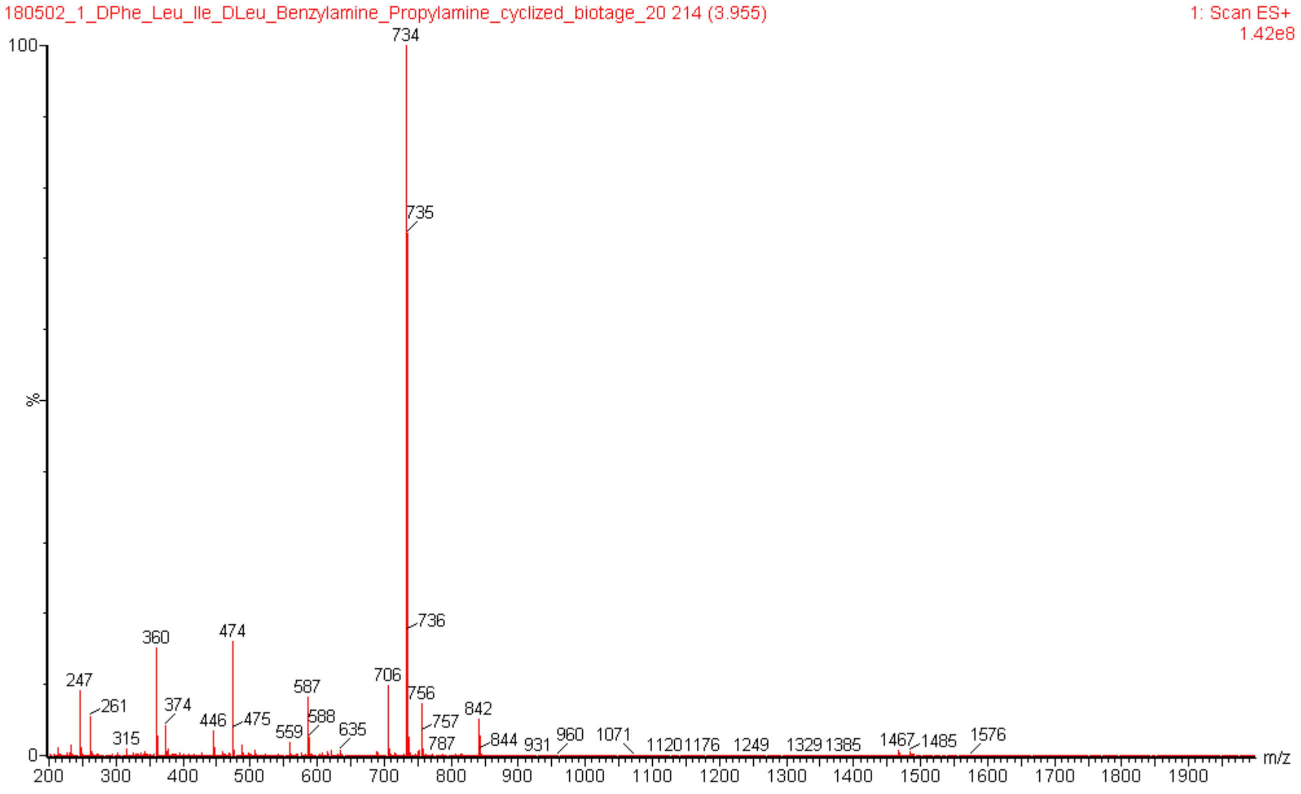

1EpDN (^{1}H NMR, 500 MHz, $CDCl_3$)

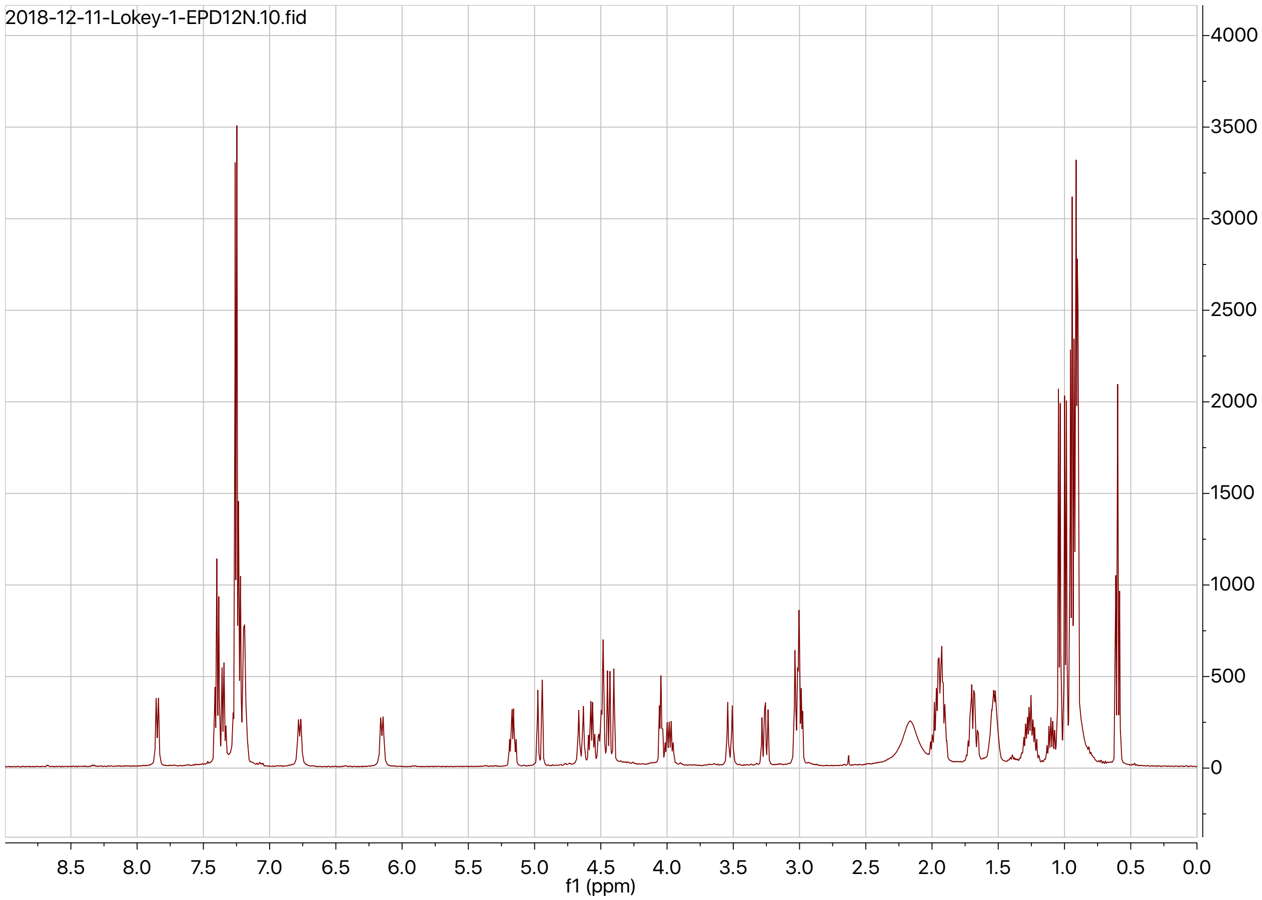

2EpDN

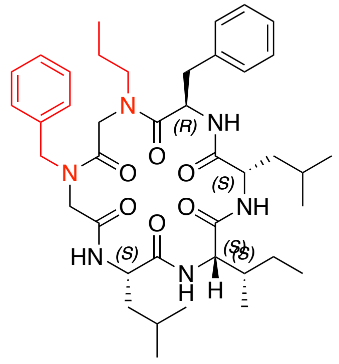

Exact Mass: 732.46
Molecular Weight: 732.97

SMILE structure:
O=C([C@H](CC(C)C)NC([C@@]([C@@H](C)CC)([H])N1)=O)N[C@@H](C(N(CC(N(CC2=CC=CC=C2)CC(N[C@@H](CC(C)C)C1=O)=O)=O)CCC)=O)CC3=CC=CC=C3

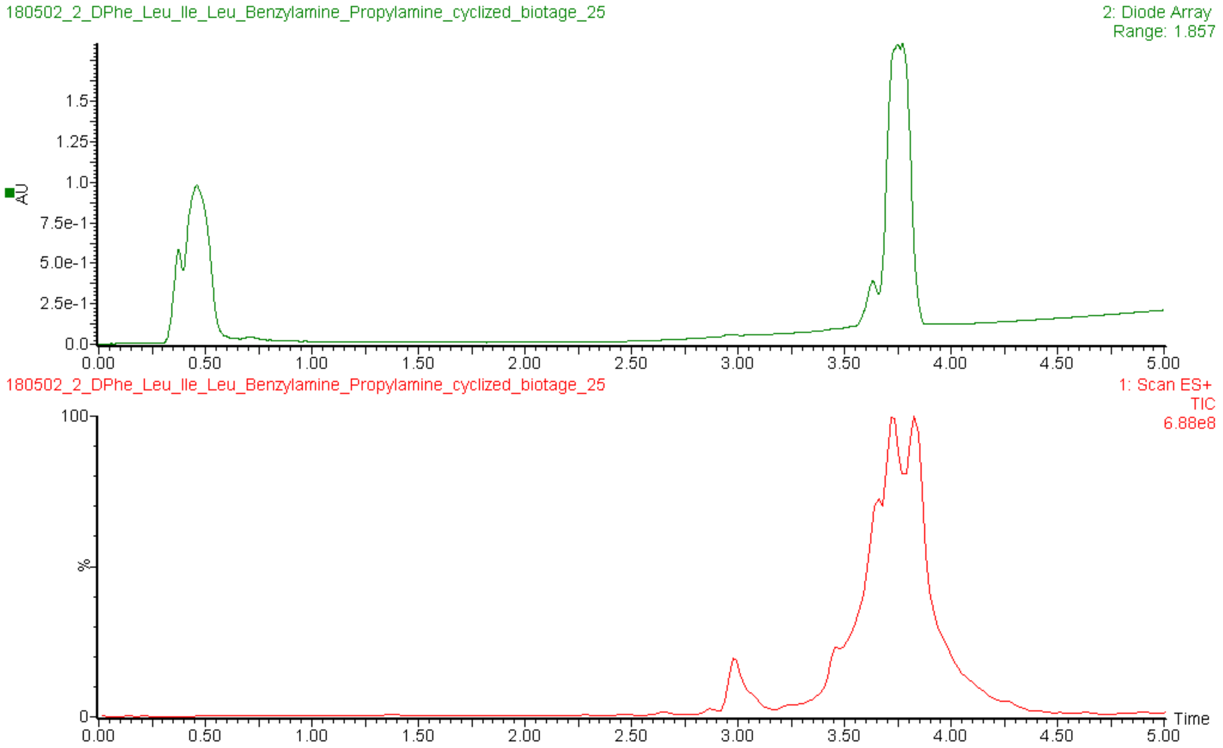

LCMS spectra

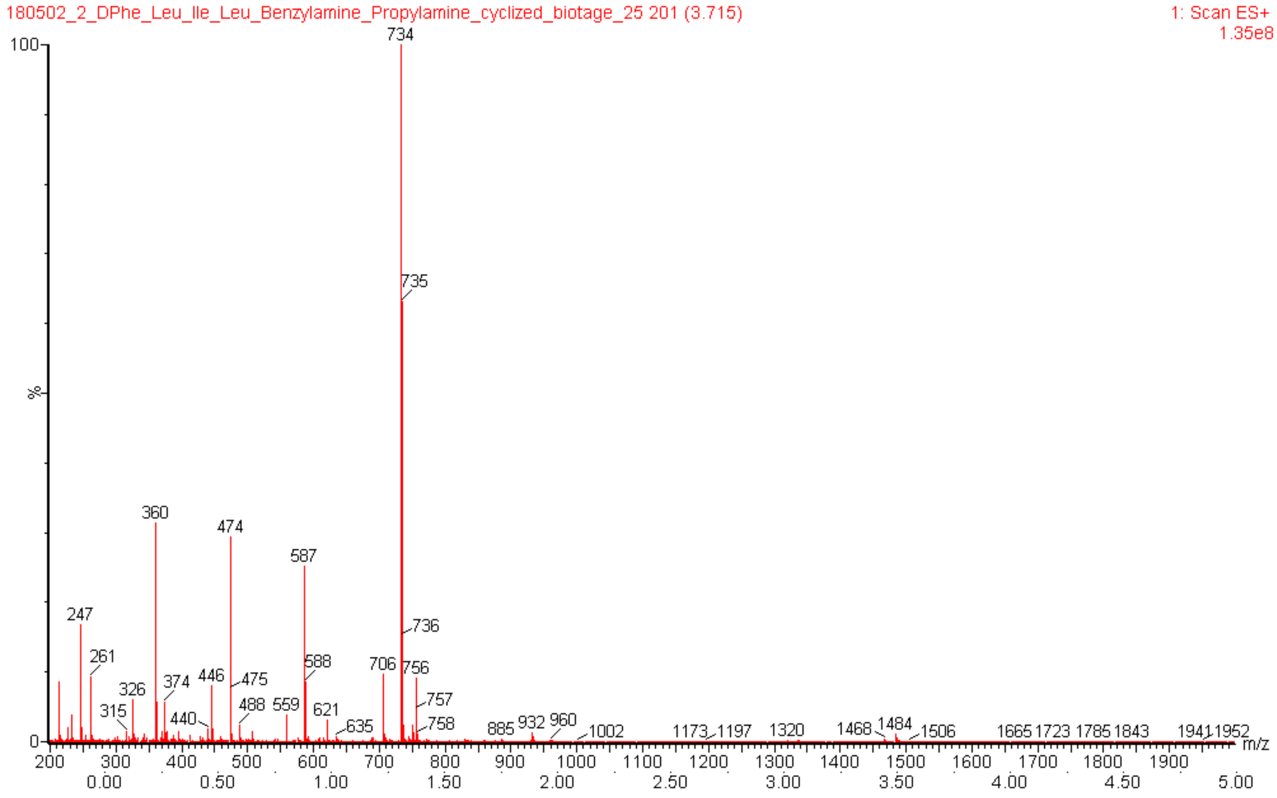

2EpDN (^{1}H NMR, 500 MHz, $CDCl_3$)

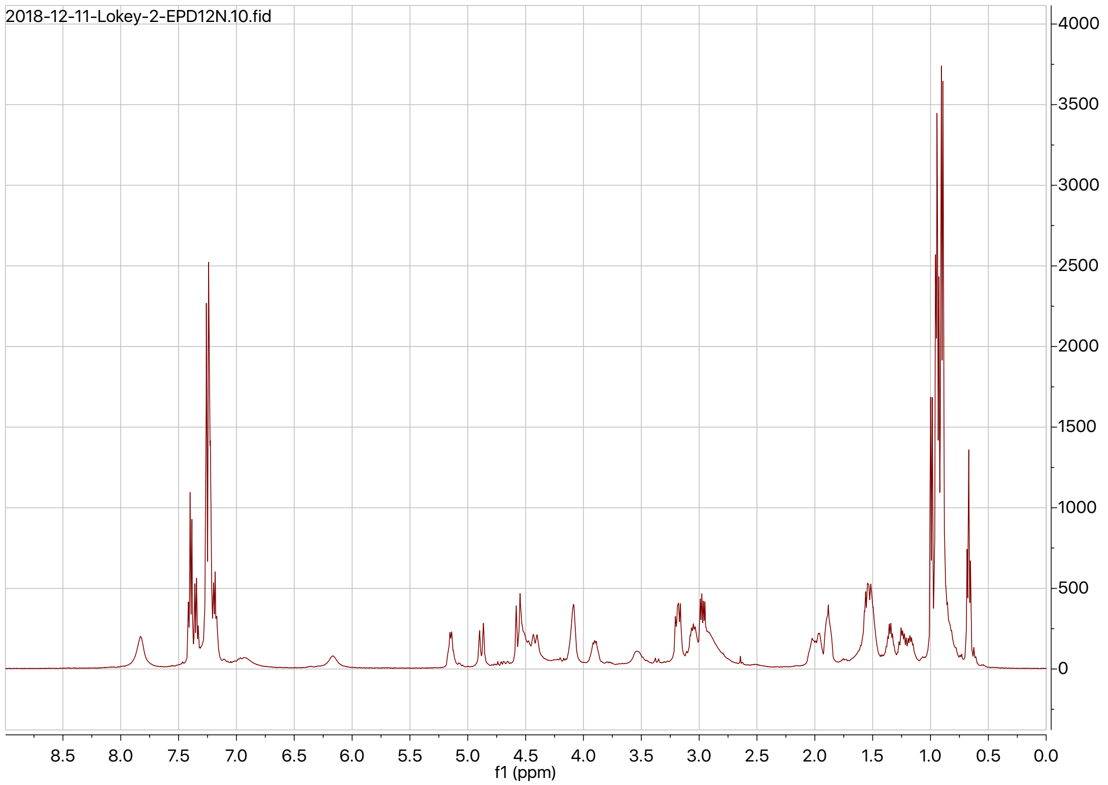

3EpDN

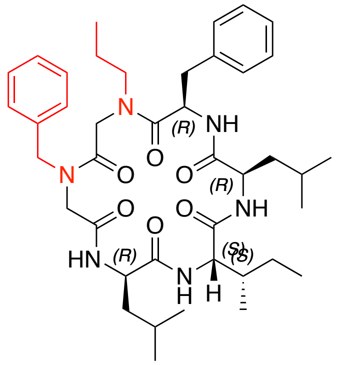

Exact Mass: 732.46
Molecular Weight: 732.97

SMILE structure:
O=C([C@@H](CC(C)C)NC([C@@]([C@@H](C)CC)([H])N1)=O)N[C@@H](C(N(CC(N(CC2=CC=CC=C2)CC(N[C@H](CC(C)C)C1=O)=O)=O)CCC)=O)CC3=CC=CC=C3

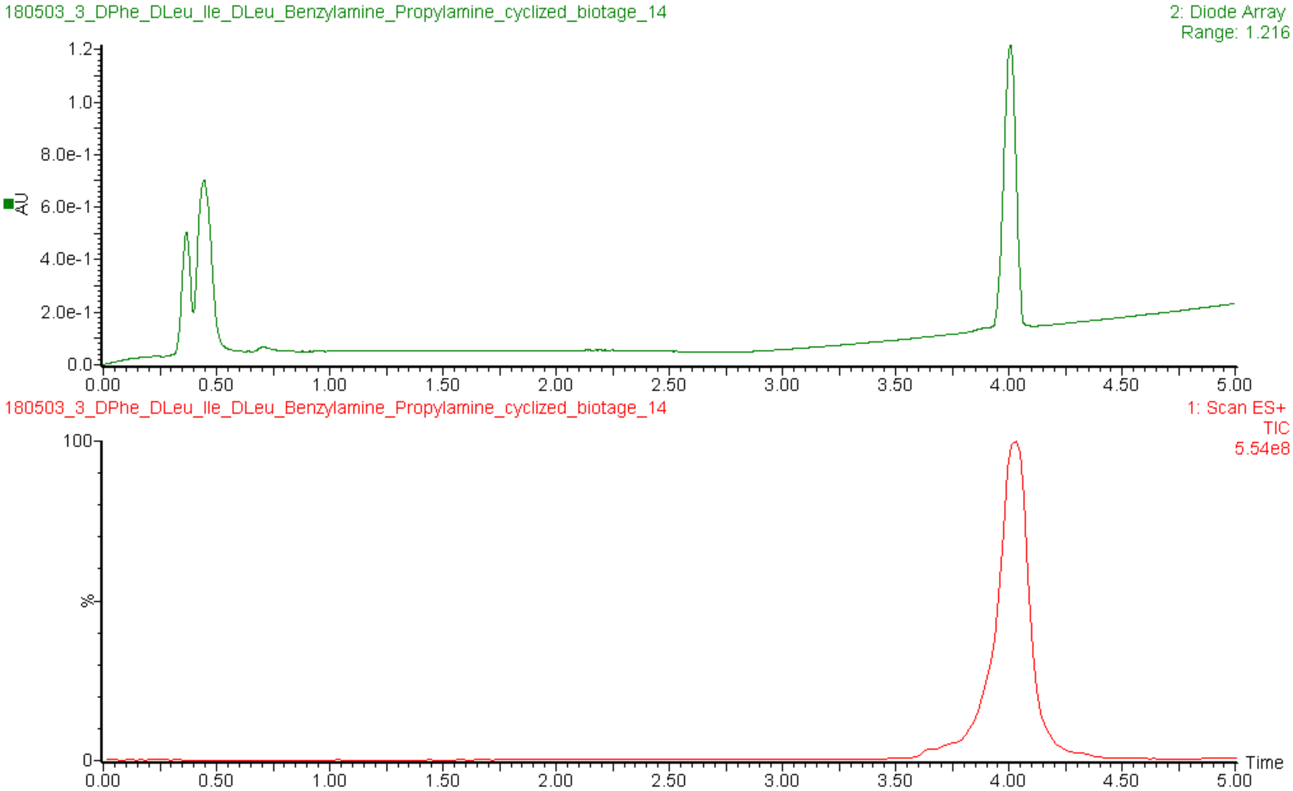

LCMS spectra

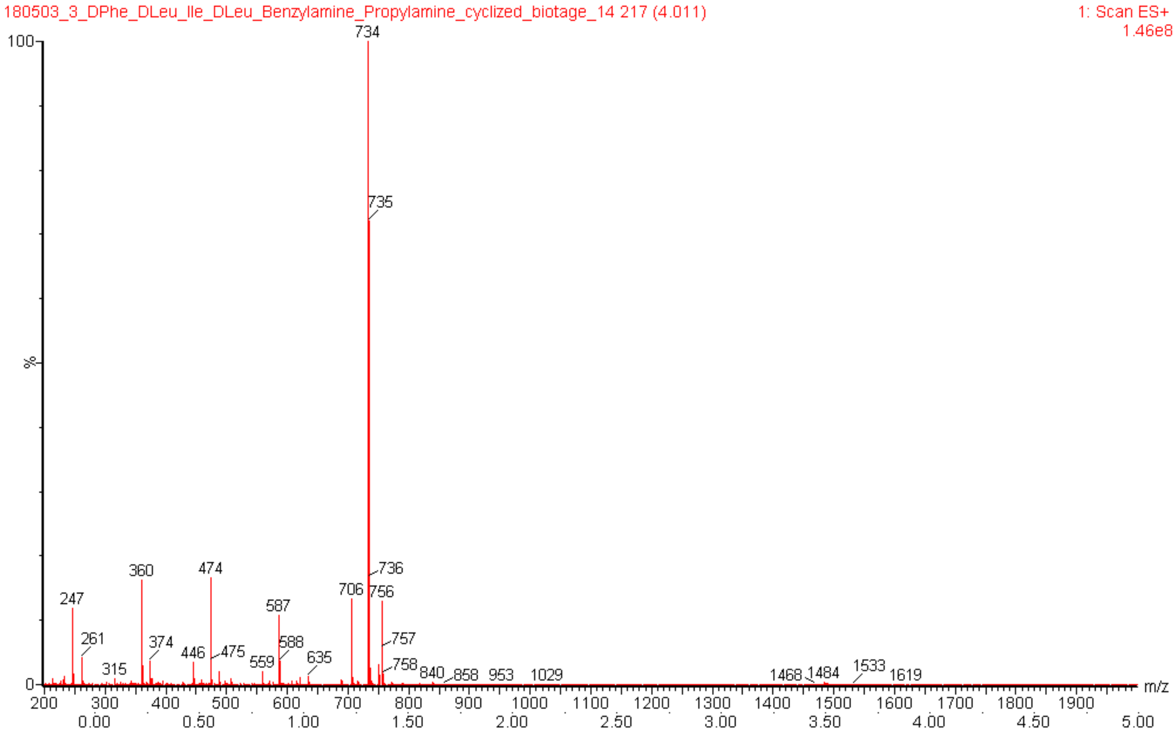

3EpDN (^{1}H NMR, 500 MHz, $CDCl_3$)

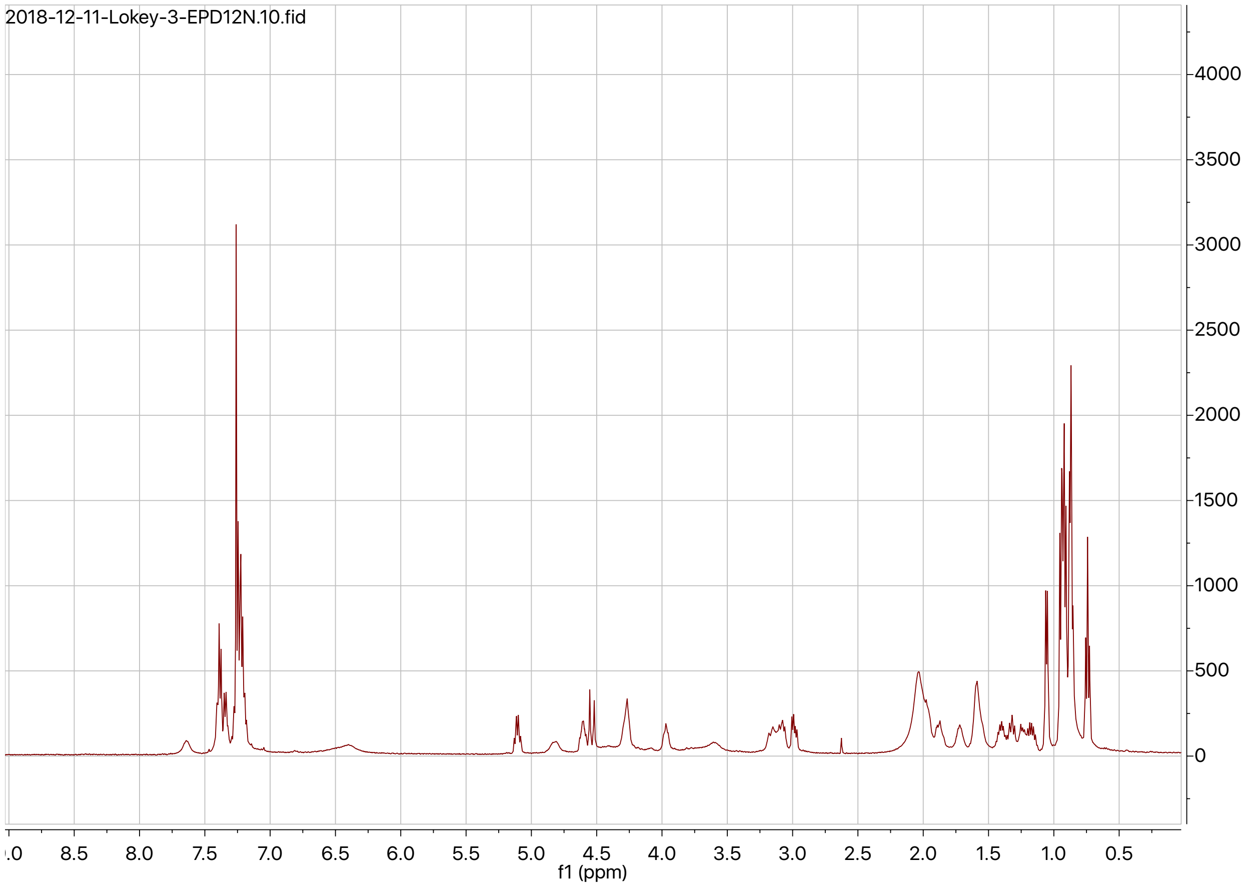

4EpDN

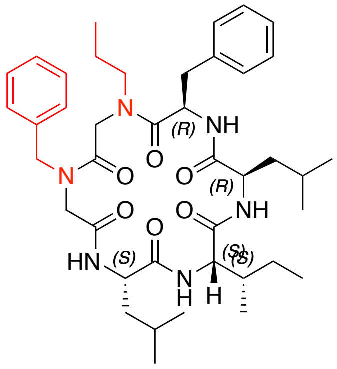

Exact Mass: 732.46
Molecular Weight: 732.97

SMILE structure:
O=C([C@@H](CC(C)C)NC([C@@]([C@@H](C)CC)([H])N1)=O)N[C@@H](C(N(CC(N(CC2=CC=CC=C2)CC(N[C@@H](CC(C)C)C1=O)=O)=O)CCC)=O)CC3=CC=CC=C3

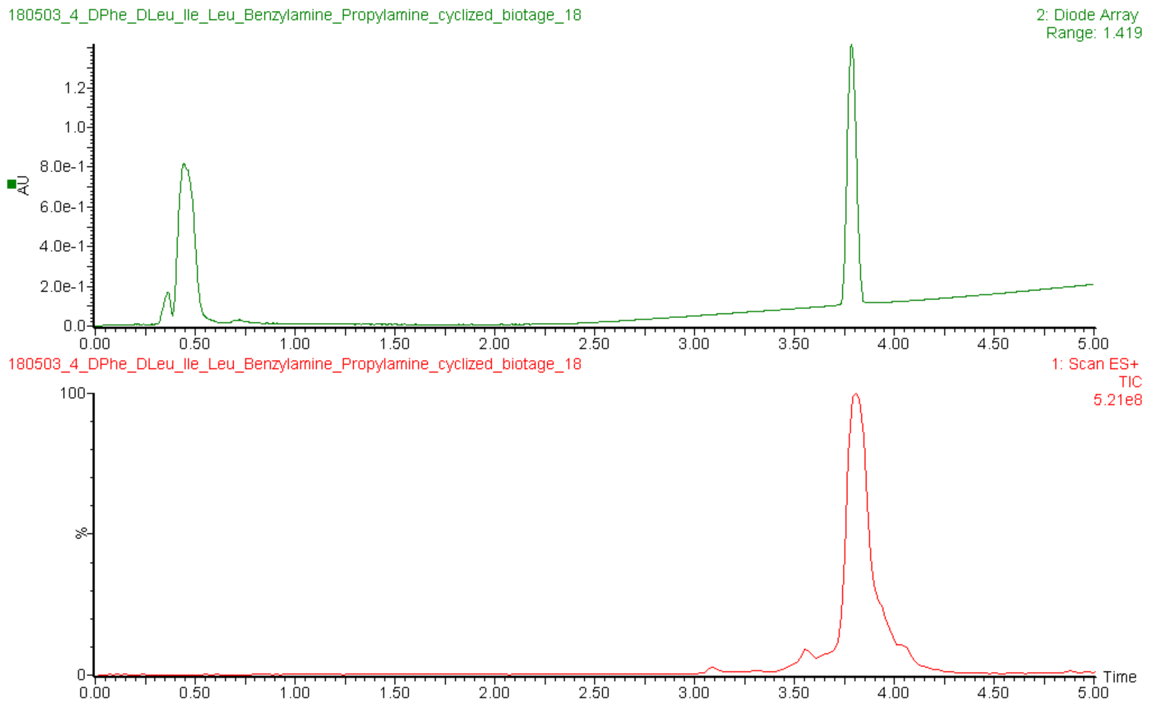

LCMS spectra

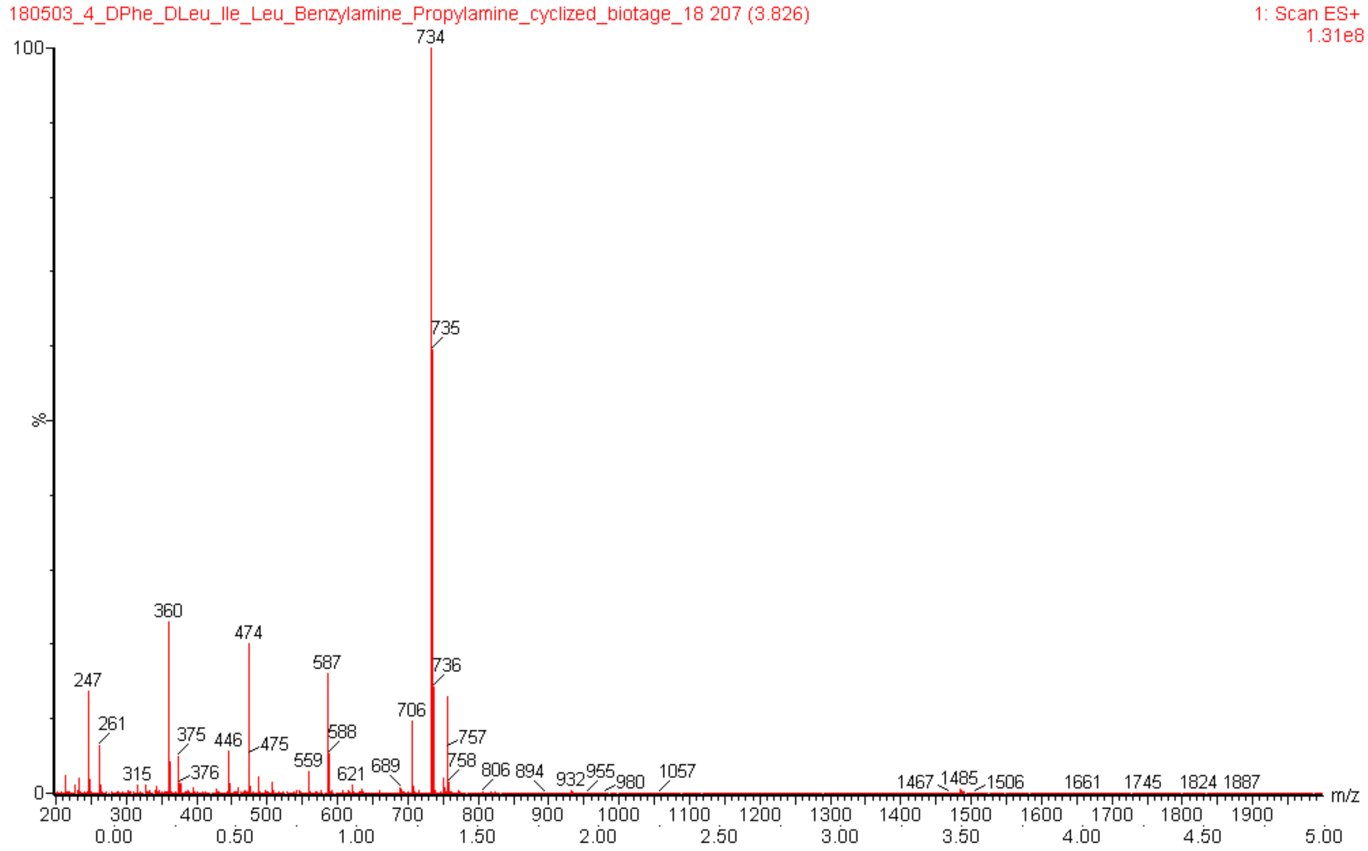

4EpDN (^{1}H NMR, 500 MHz, $CDCl_3$)

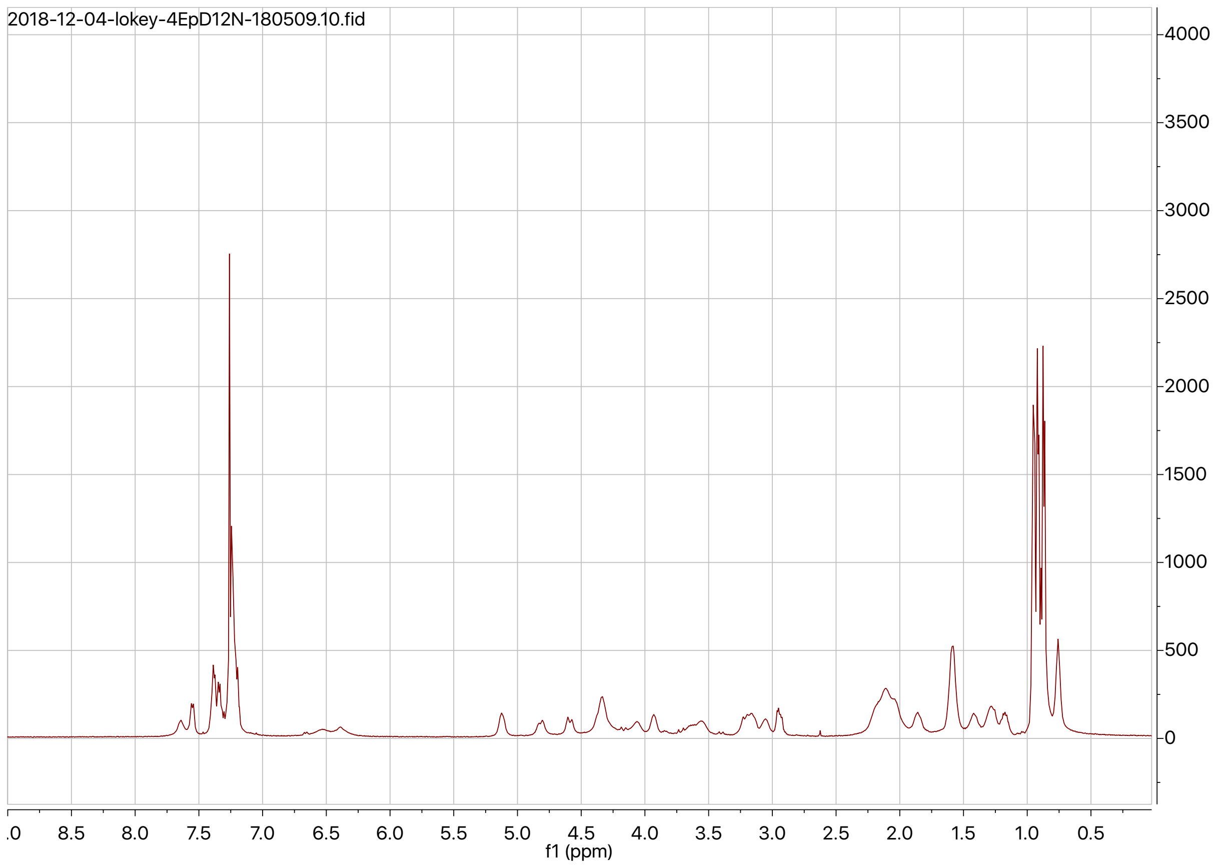

5EpDN

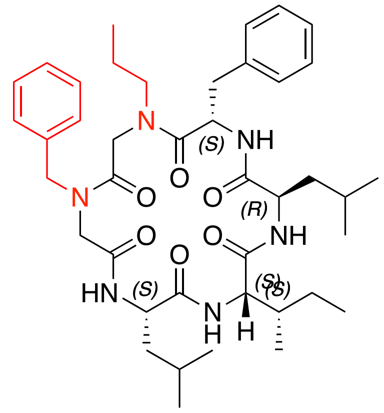

Exact Mass: 732.46
Molecular Weight: 732.97

SMILE structure:
O=C([C@@H](CC(C)C)NC([C@@]([C@@H](C)CC)([H])N1)=O)N[C@H](C(N(CC(N(CC2=CC=CC=C2)CC(N[C@@H](CC(C)C)C1=O)=O)=O)CCC)=O)CC3=CC=CC=C3

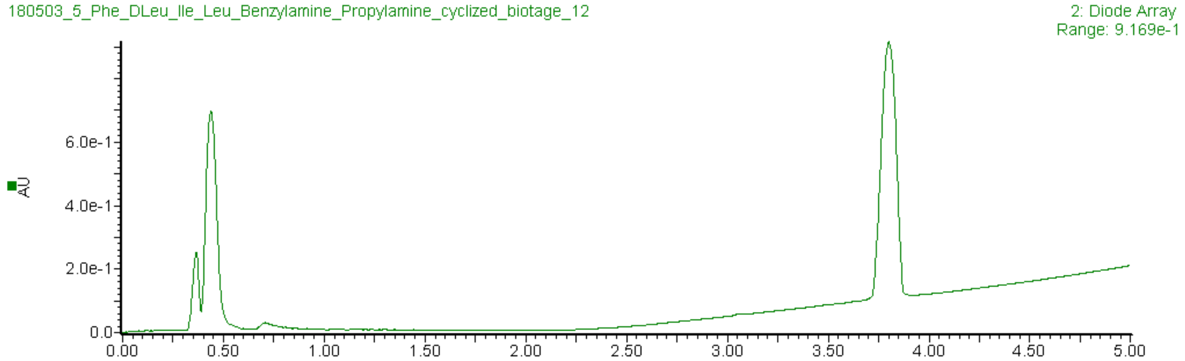

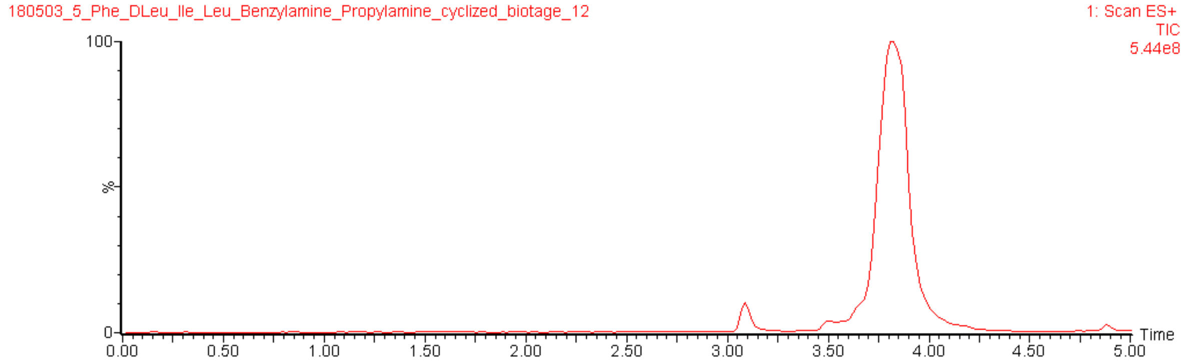

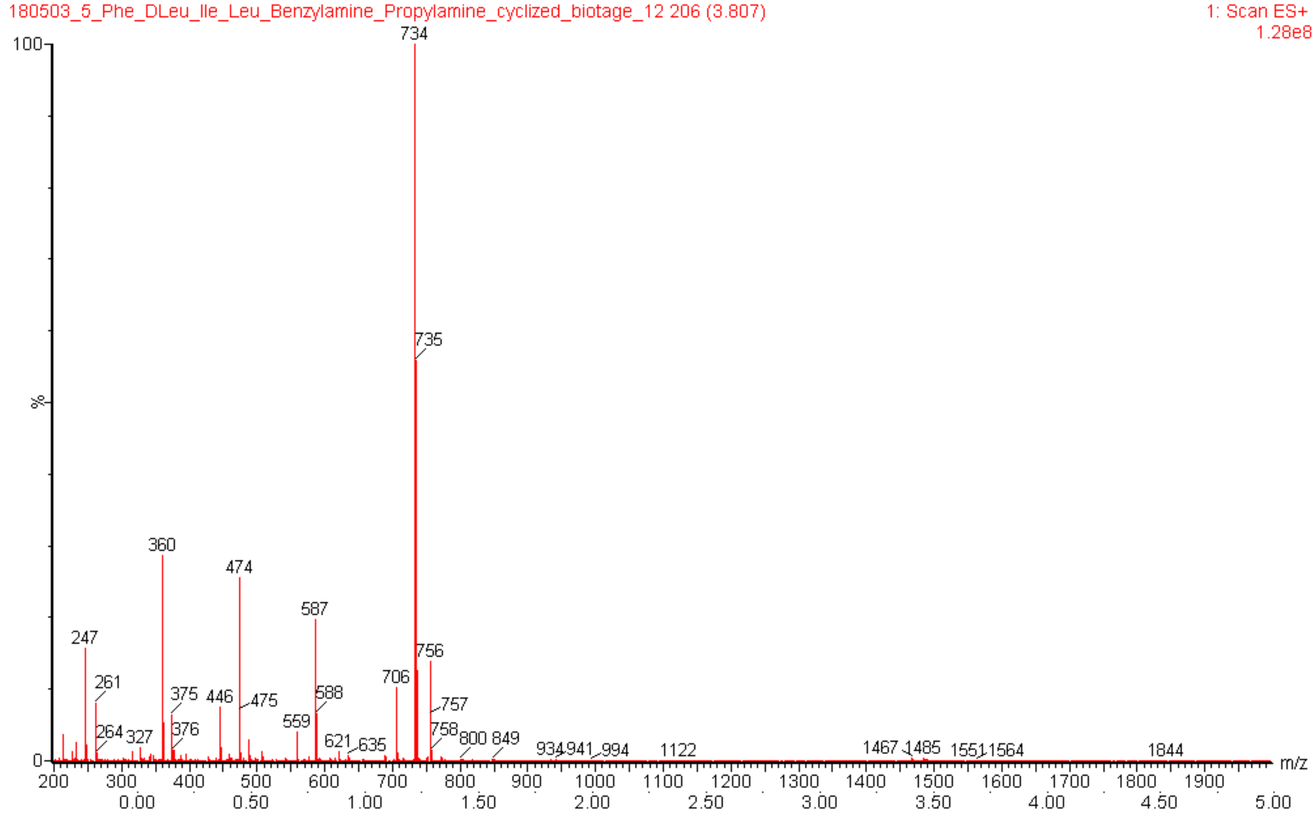

5EpDN (^{1}H NMR, 500 MHz, $CDCl_3$)

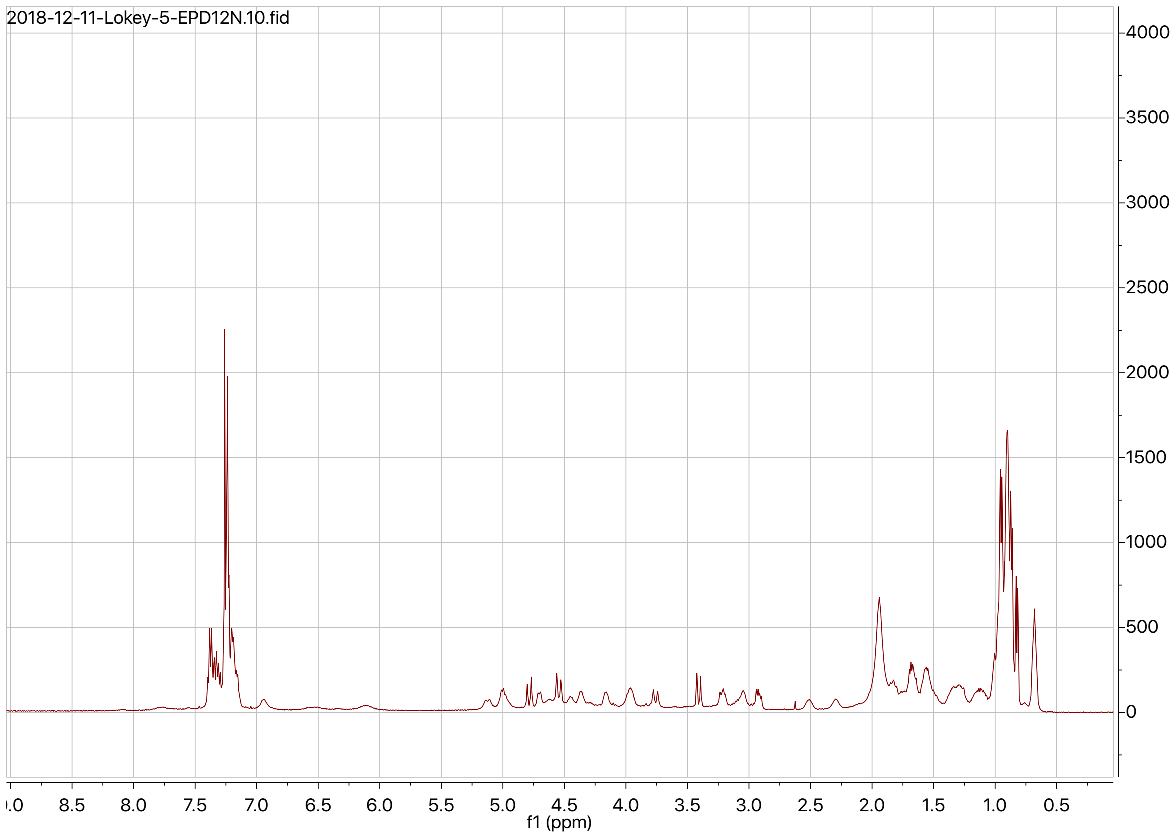

6EpDN

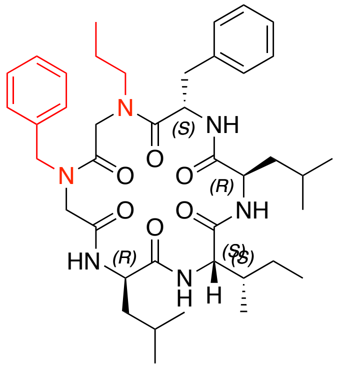

Exact Mass: 732.46
Molecular Weight: 732.97

SMILE structure:
O=C([C@@H](CC(C)C)NC([C@@]([C@@H](C)CC)([H])N1)=O)N[C@H](C(N(CC(N(CC2=CC=CC=C2)CC(N[C@H](CC(C)C)C1=O)=O)=O)CCC)=O)CC3=CC=CC=C3

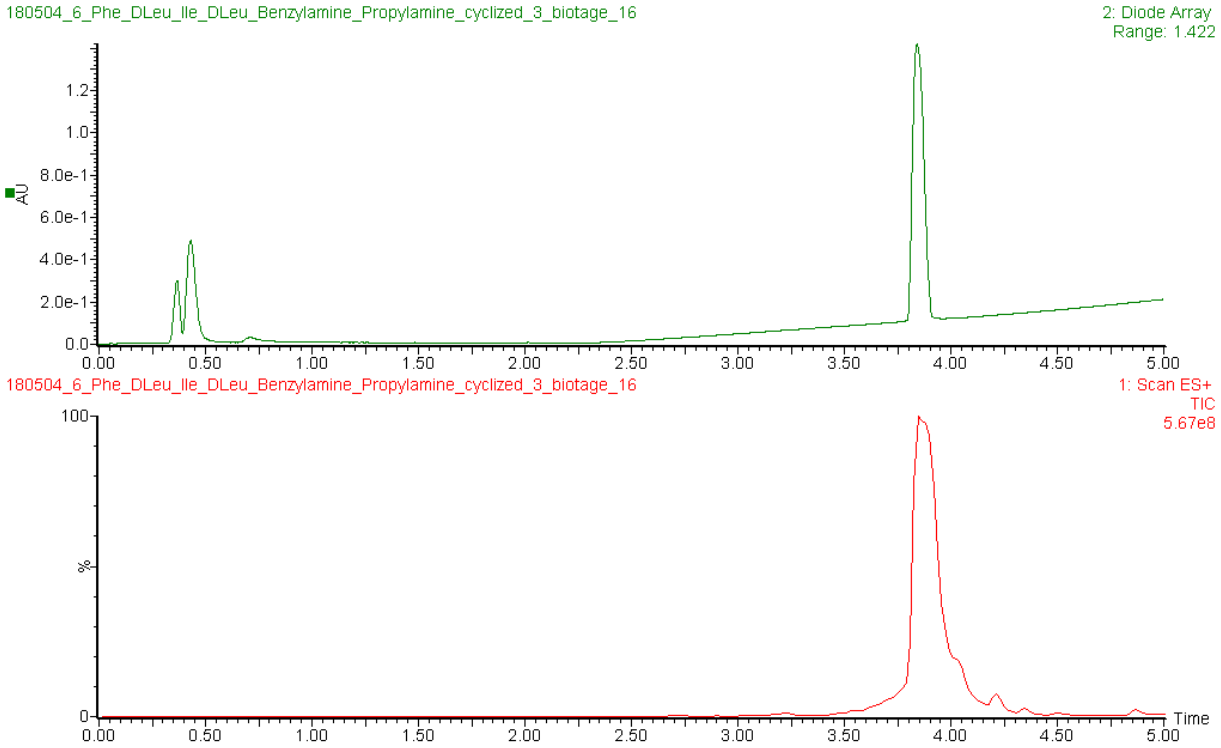

LCMS spectra

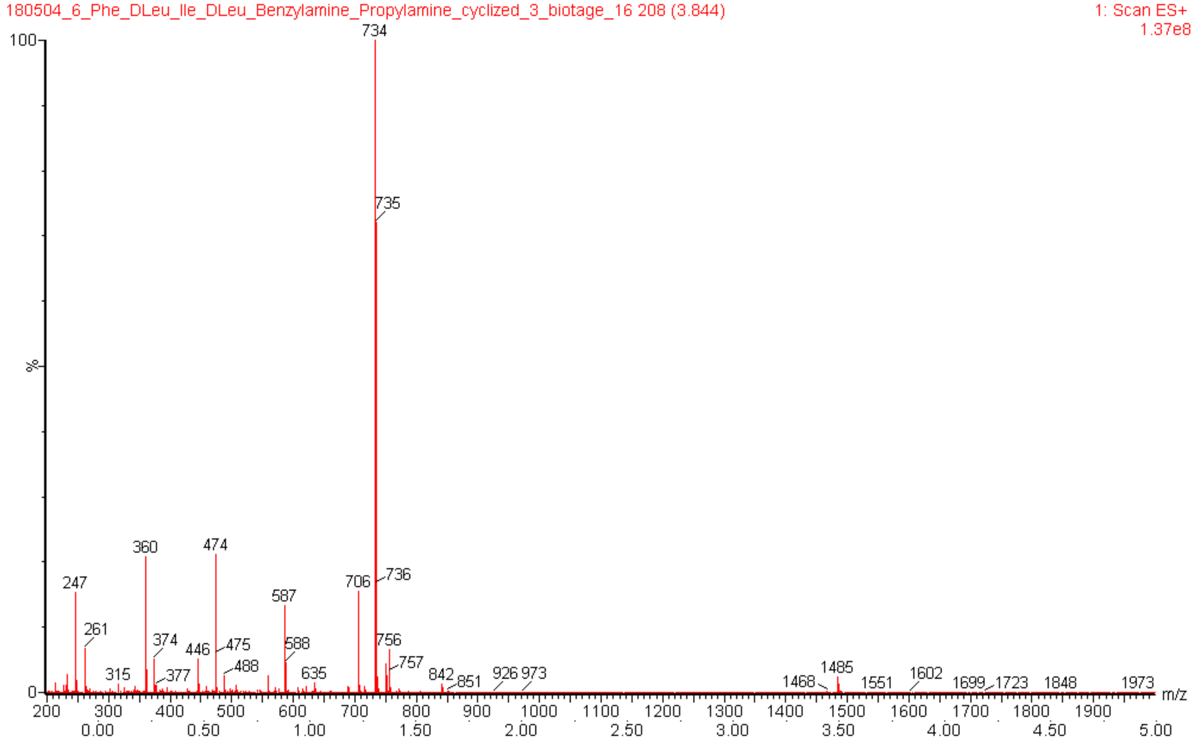

6EpDN (^1H NMR, 500 MHz, $CDCl_3$)

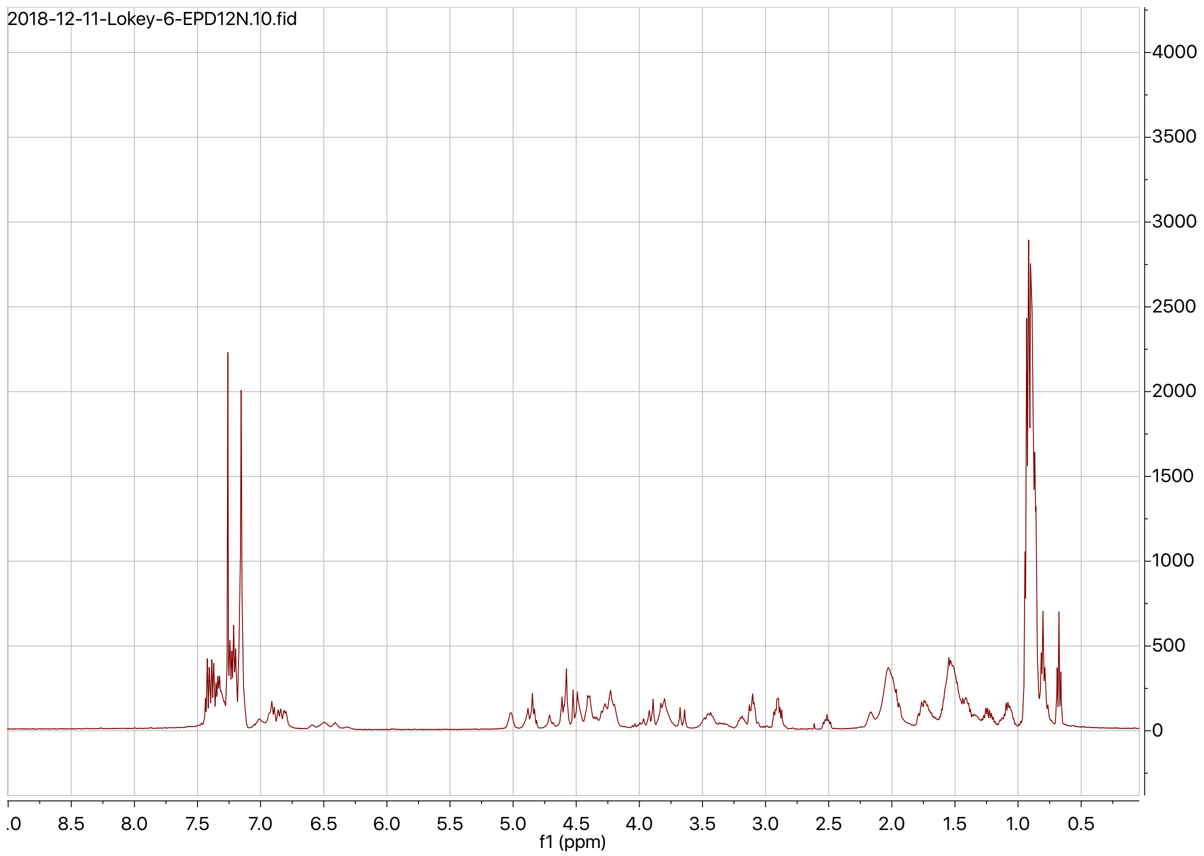

7EpDN

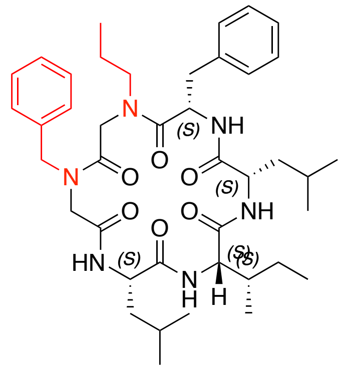

Exact Mass: 732.46
Molecular Weight: 732.97

SMILE structure:
O=C([C@H](CC(C)C)NC([C@@]([C@@H](C)CC)([H])N1)=O)N[C@H](C(N(CC(N(CC2=CC=CC=C2)CC(N[C@@H](CC(C)C)C1=O)=O)=O)CCC)=O)CC3=CC=CC=C3

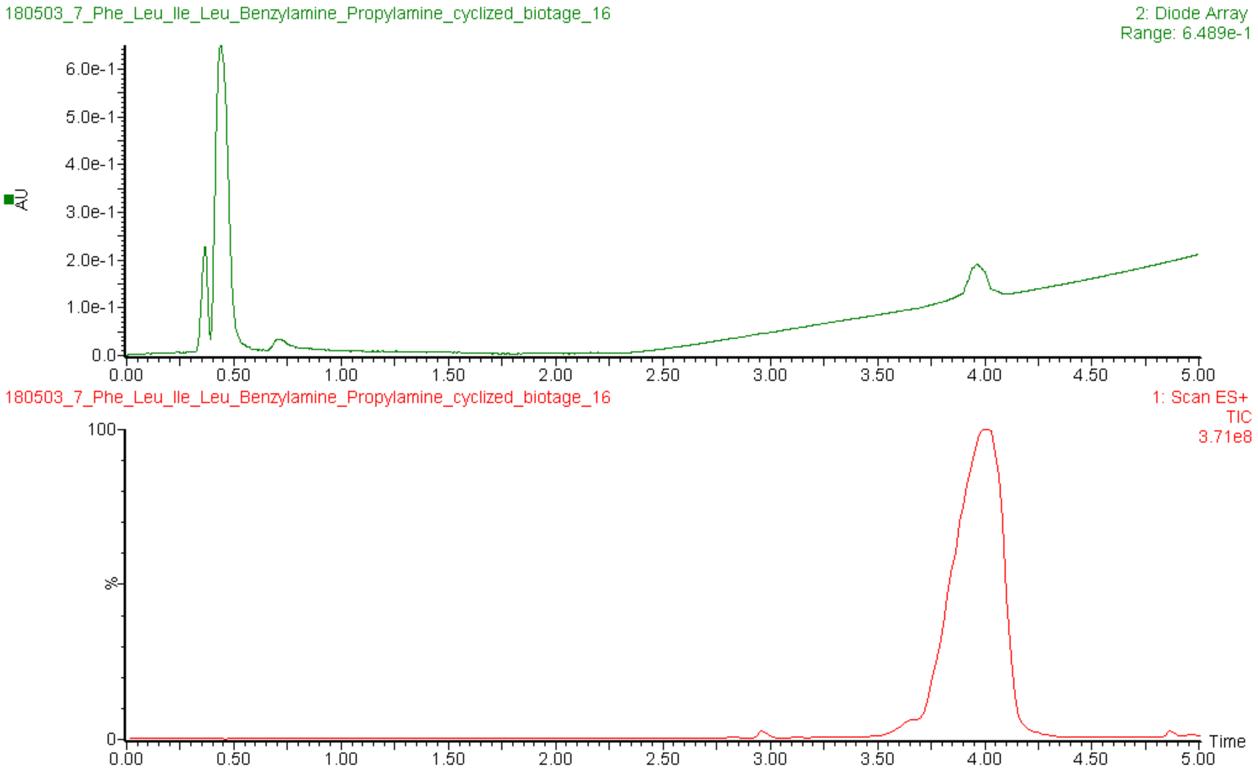

LCMS spectra

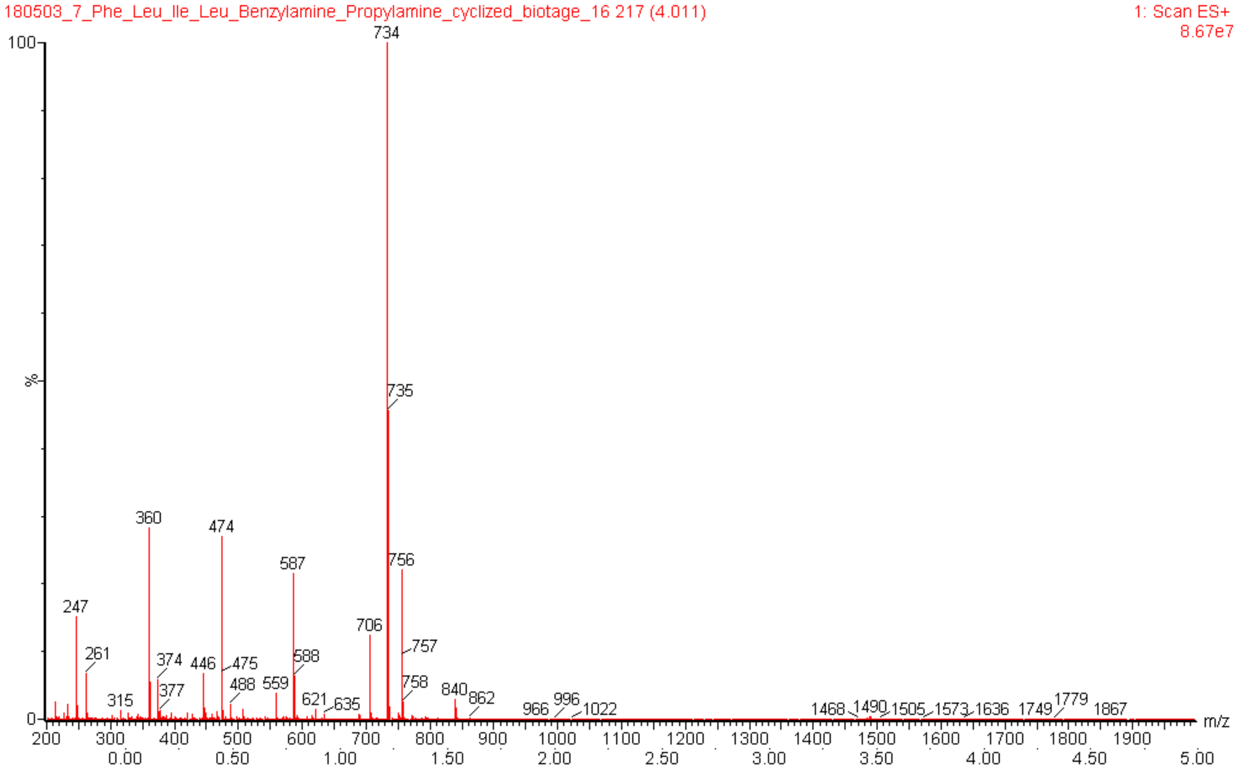

7EpDN (^{1}H NMR, 500 MHz, $CDCl_3$)

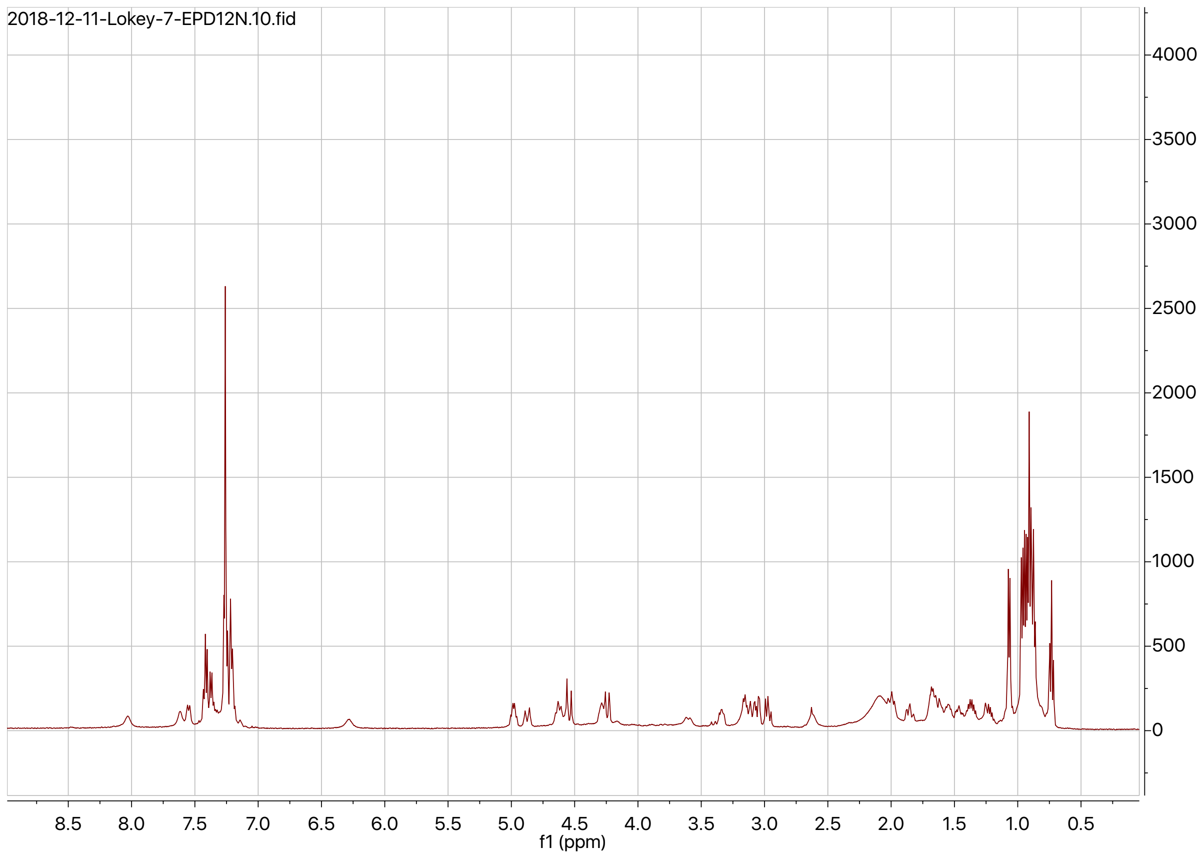

8EpDN

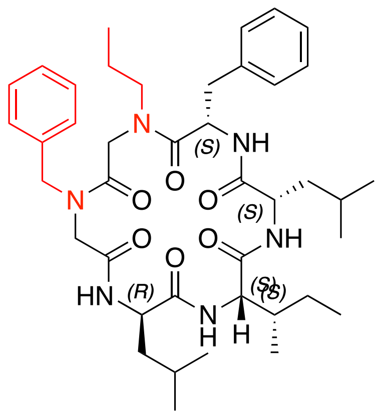

Exact Mass: 732.46
Molecular Weight: 732.97

SMILE structure:
O=C([C@H](CC(C)C)NC([C@@]([C@@H](C)CC)([H])N1)=O)N[C@H](C(N(CC(N(CC2=CC=CC=C2)CC(N[C@H](CC(C)C)C1=O)=O)=O)CCC)=O)CC3=CC=CC=C3

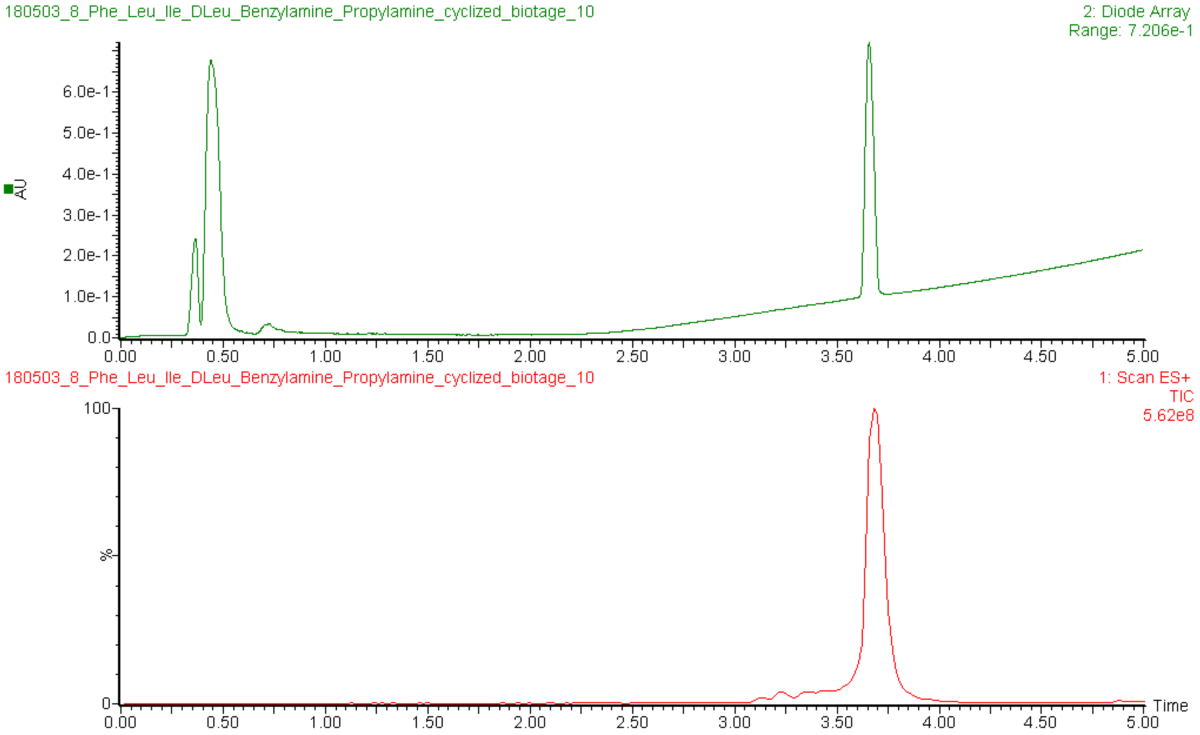

LCMS spectra

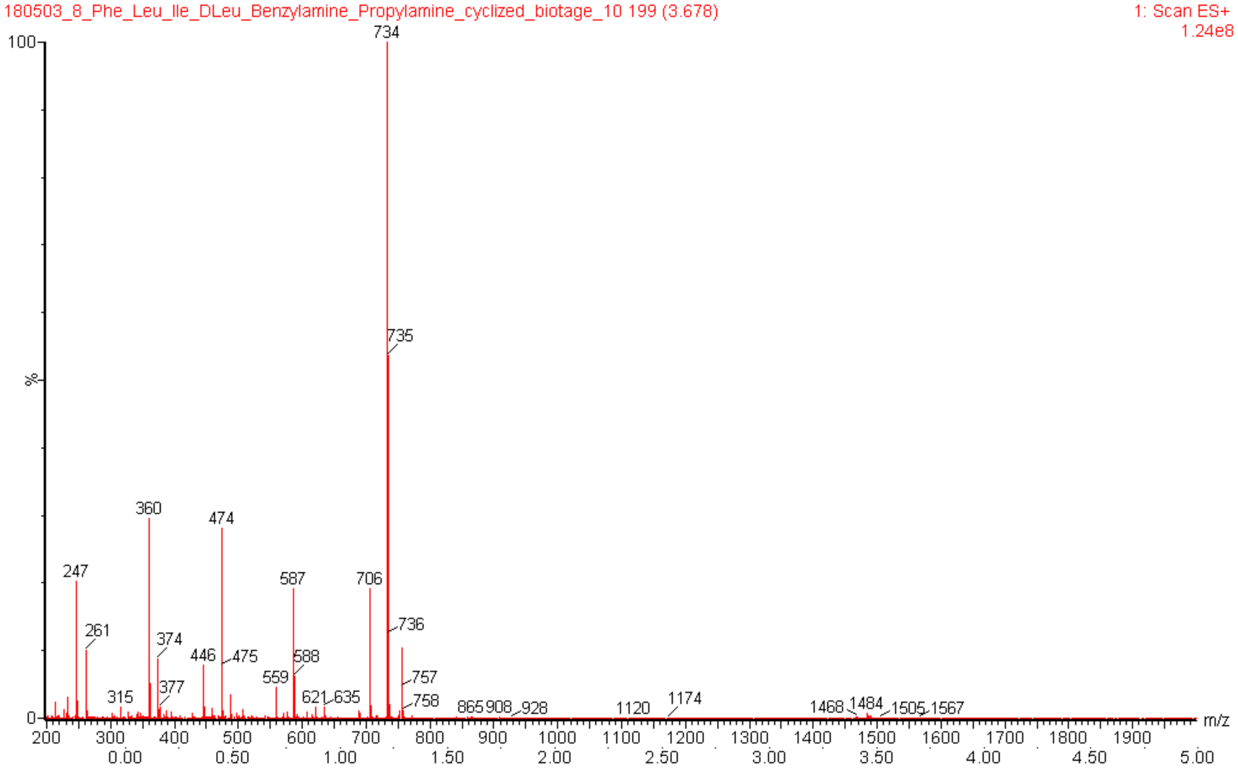

8EpDN (^1H NMR, 500 MHz, $CDCl_3$)

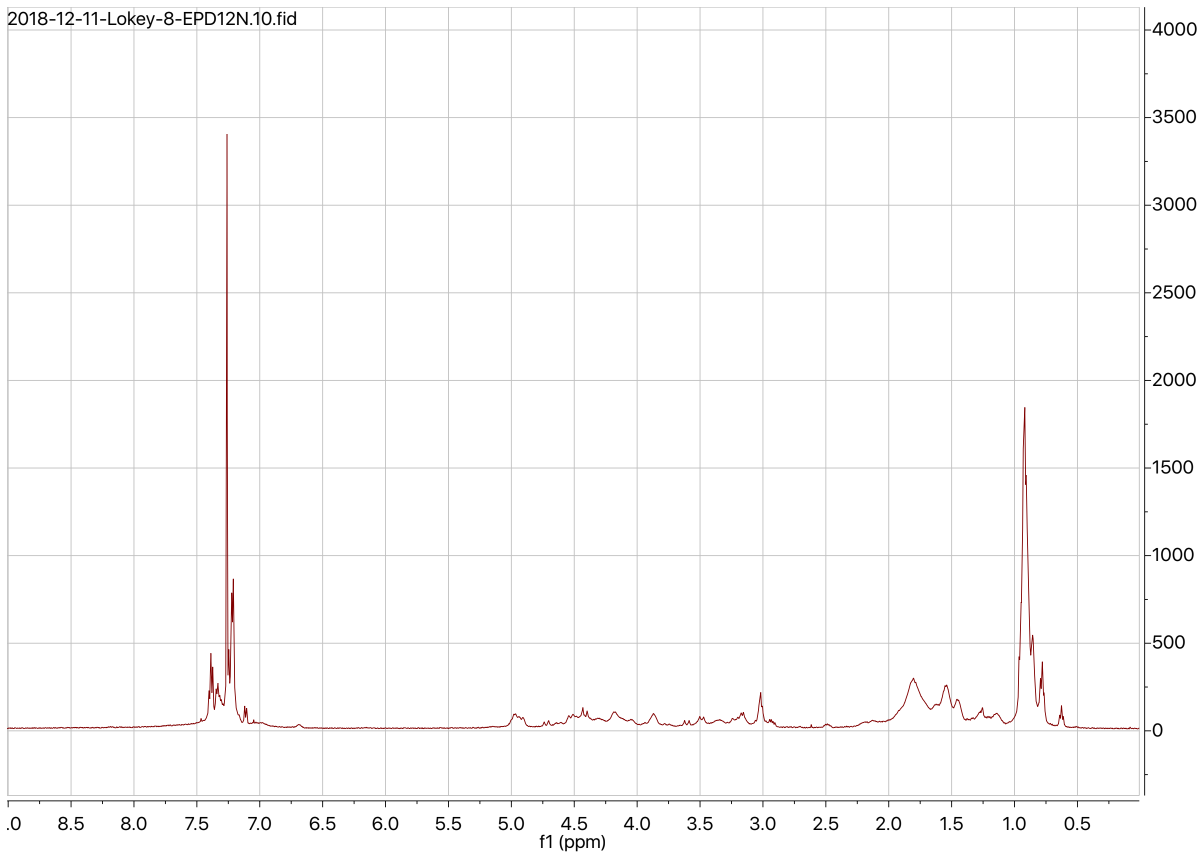

9EpDN

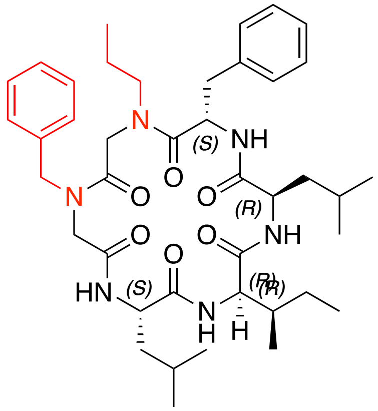

Exact Mass: 732.46
Molecular Weight: 732.97

SMILE structure:
O=C([C@@H](CC(C)C)NC([C@]([C@H](C)CC)([H])N1)=O)N[C@H](C(N(CC(N(CC2=CC=CC=C2)CC(N[C@@H](CC(C)C)C1=O)=O)=O)CCC)=O)CC3=CC=CC=C3

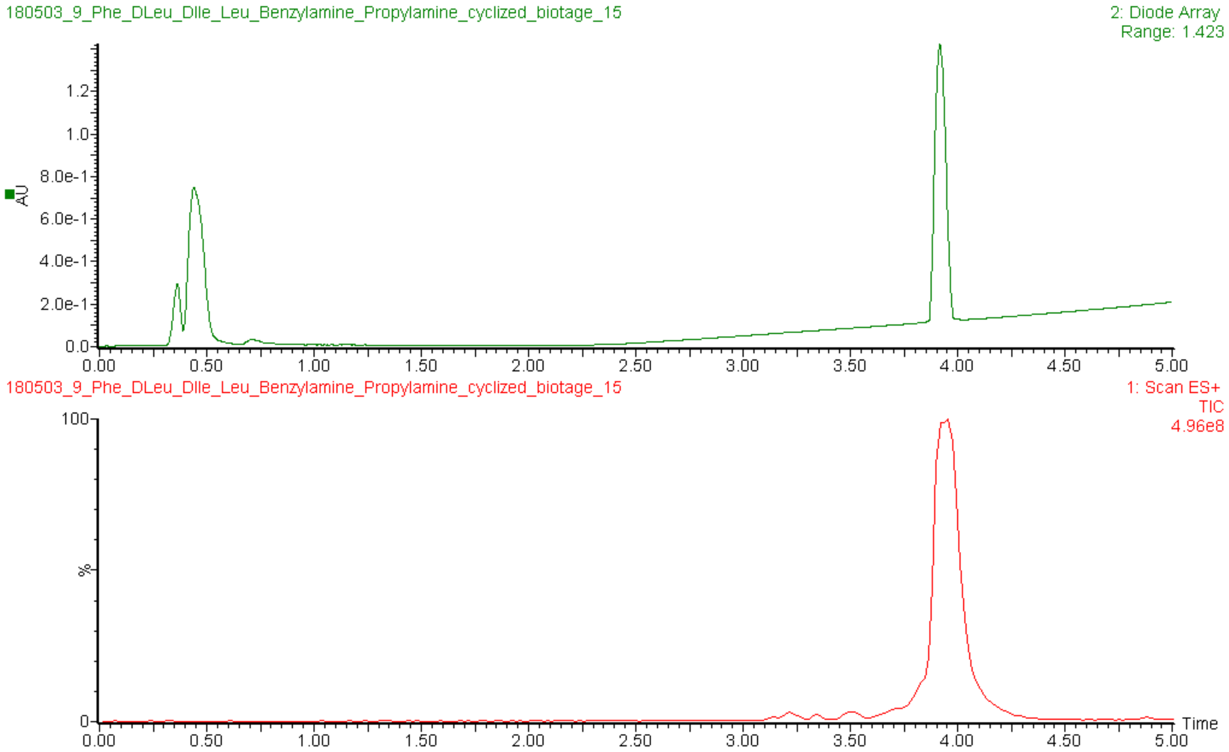

LCMS spectra

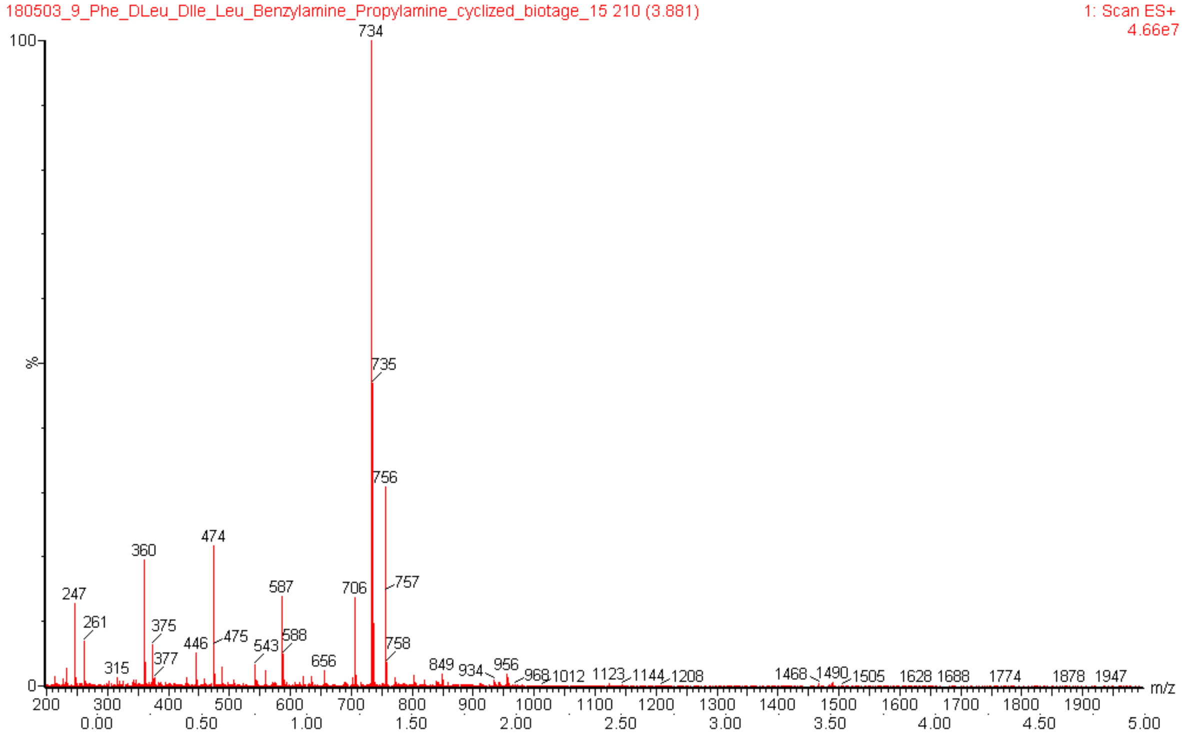

9EpDN (^{1}H NMR, 500 MHz, $CDCl_3$)

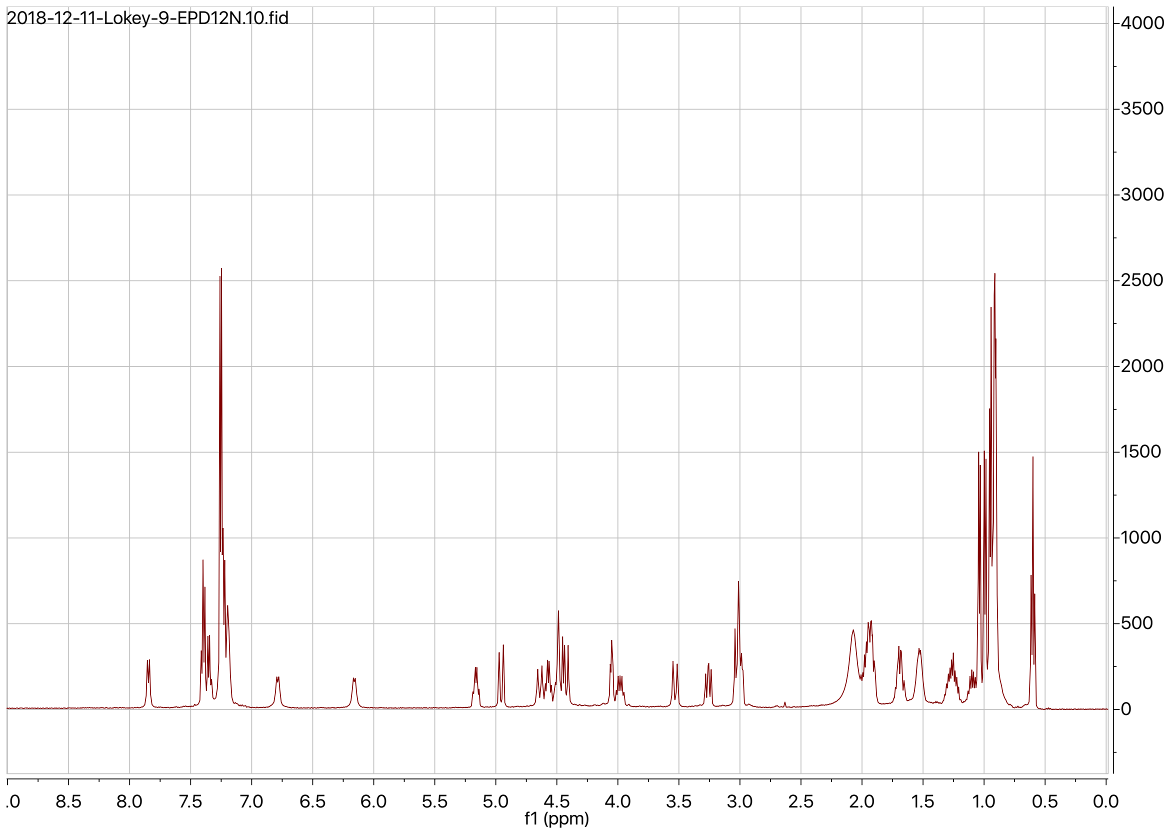
2018-12-11-Lokey-9-EPD12N.10.fid
f1 (ppm)
.0
8.5
8.0
7.5
7.0
6.5
6.0
5.5
5.0
4.5
4.0
3.5
3.0
2.5
2.0
1.5
1.0
0.5
0.0
4000
3500
3000
2500
2000
1500
1000
500
0

1EpDN 6Ala

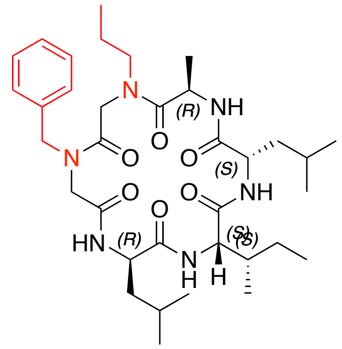

Exact Mass: 656.43
Molecular Weight: 656.87

SMILE structure:
O=C([C@H](CC(C)C)NC([C@@]([C@@H](C)CC)
([H])N1)=O)N[C@@H](C(N(CC(N(CC2=CC=CC=
C2)CC(N[C@H](CC(C)C)C1=O)=O)=O)CCC)=O)C

1EpDN 6Ala (^{1}H NMR, 500 MHz, $CDCl_3$)

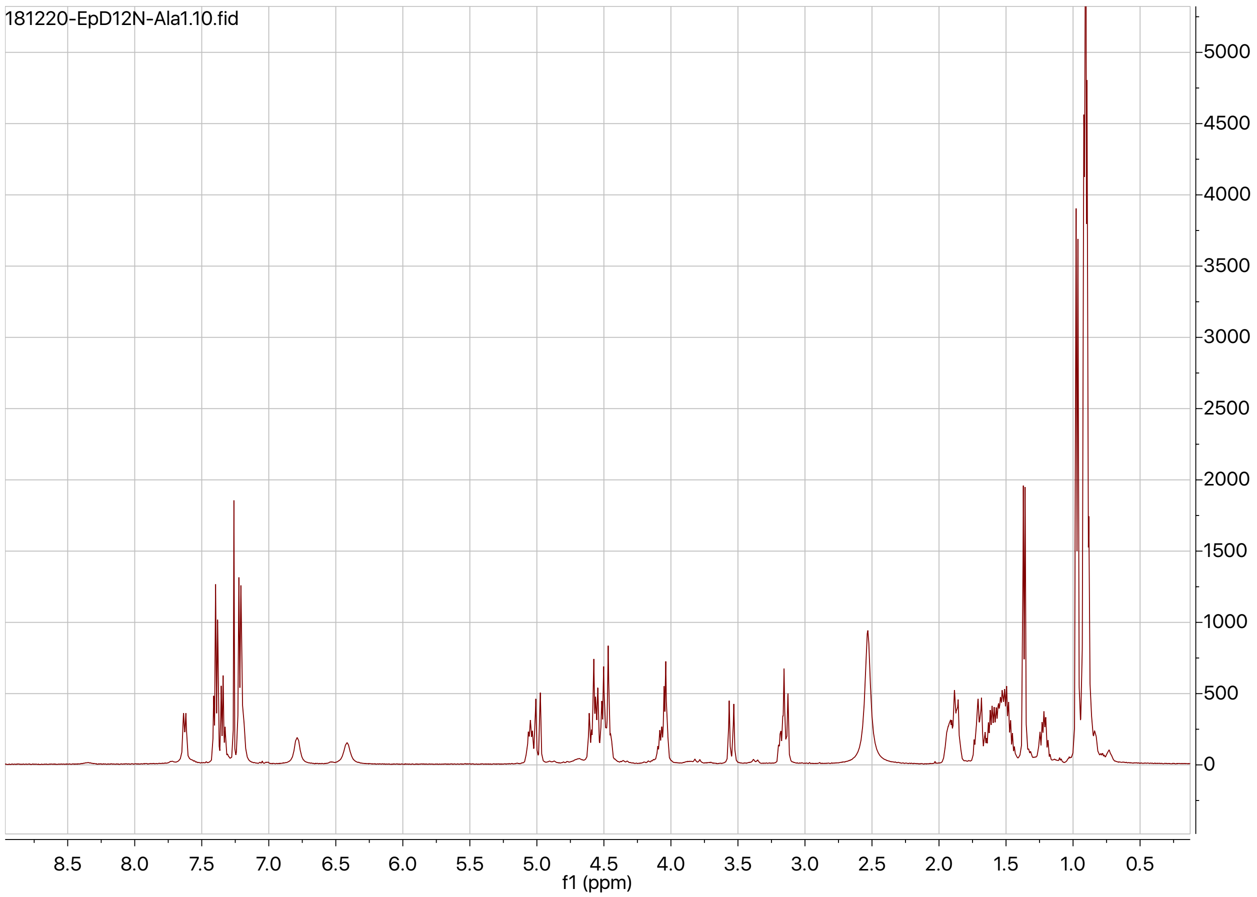

1EpDN 5Ala

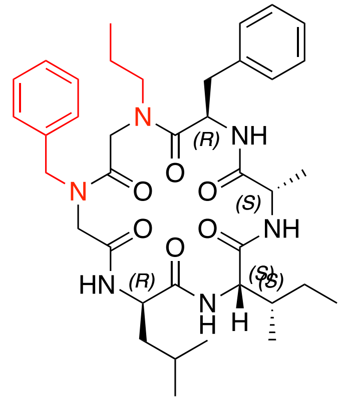

Exact Mass: 690.41
Molecular Weight: 690.89

SMILE structure:
C[C@@H](C(N[C@@H](C(N(CC(N(CC
1=CC=CC=C1)CC(N[C@H](CC(C)C)C2=
O)=O)=O)CCC)=O)CC3=CC=CC=C3)=O)
NC([C@@]([C@@H](C)CC)([H])N2)=O

1EpDN 5Ala (^{1}H NMR, 500 MHz, $CDCl_3$)

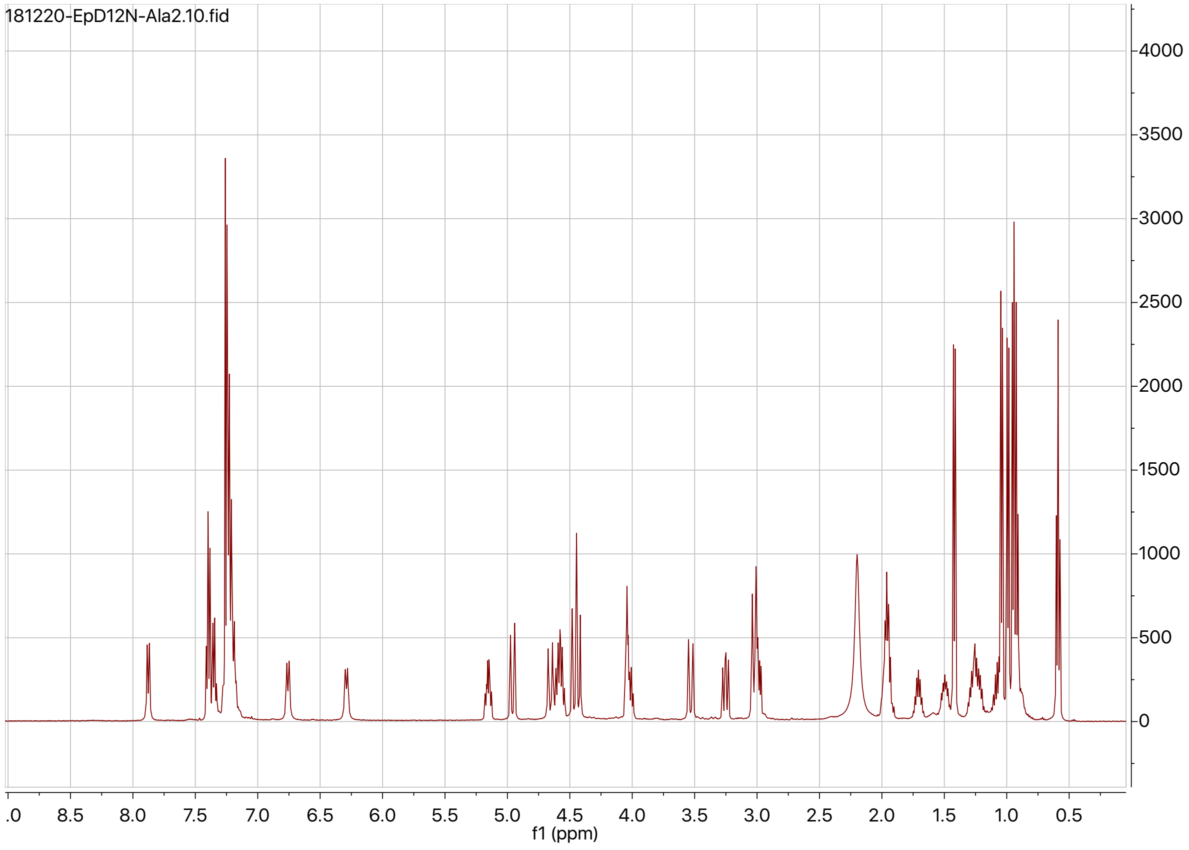

1EpDN 4Ala

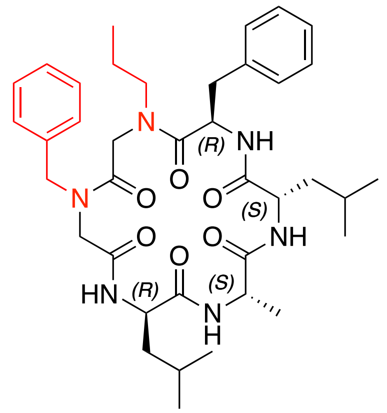

Exact Mass: 690.41
Molecular Weight: 690.89

SMILE structure:
O=C([C@H](CC(C)C)NC([C@H](C)N1)=O)N[C@@
H](C(N(CC(N(CC2=CC=CC=C2)CC(N[C@H](CC(C)C
)C1=O)=O)=O)CCC)=O)CC3=CC=CC=C3

1EpDN 4Ala (^1H NMR, 500 MHz, $CDCl_3$)

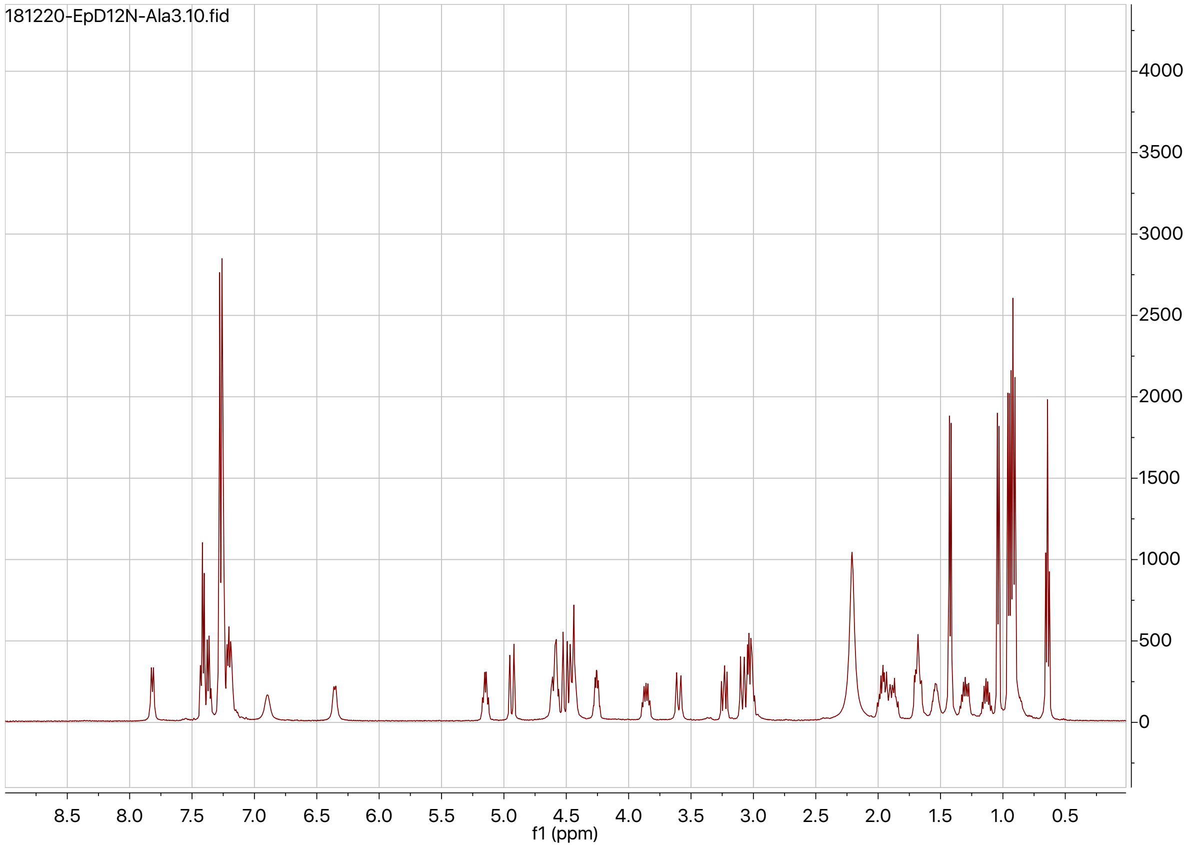

1EpDN 3Ala

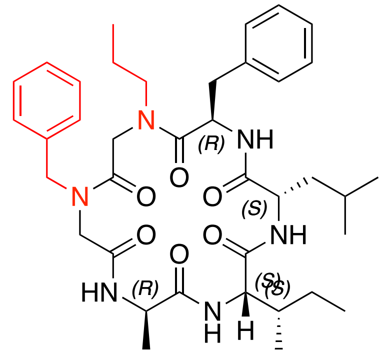

Exact Mass: 690.41
Molecular Weight: 690.89

SMILE structure:
O=C([C@H](CC(C)C)NC([C@@]([C@@H](C)CC)([H])N1)=O)N[C@@H](C(N(CC(N(CC2=CC=CC=C2)CC(N[C@H](C)C1=O)=O)=O)CCC)=O)CC3=CC=CC=C3

1EpDN 3Ala (^{1}H NMR, 500 MHz, $CDCl_3$)

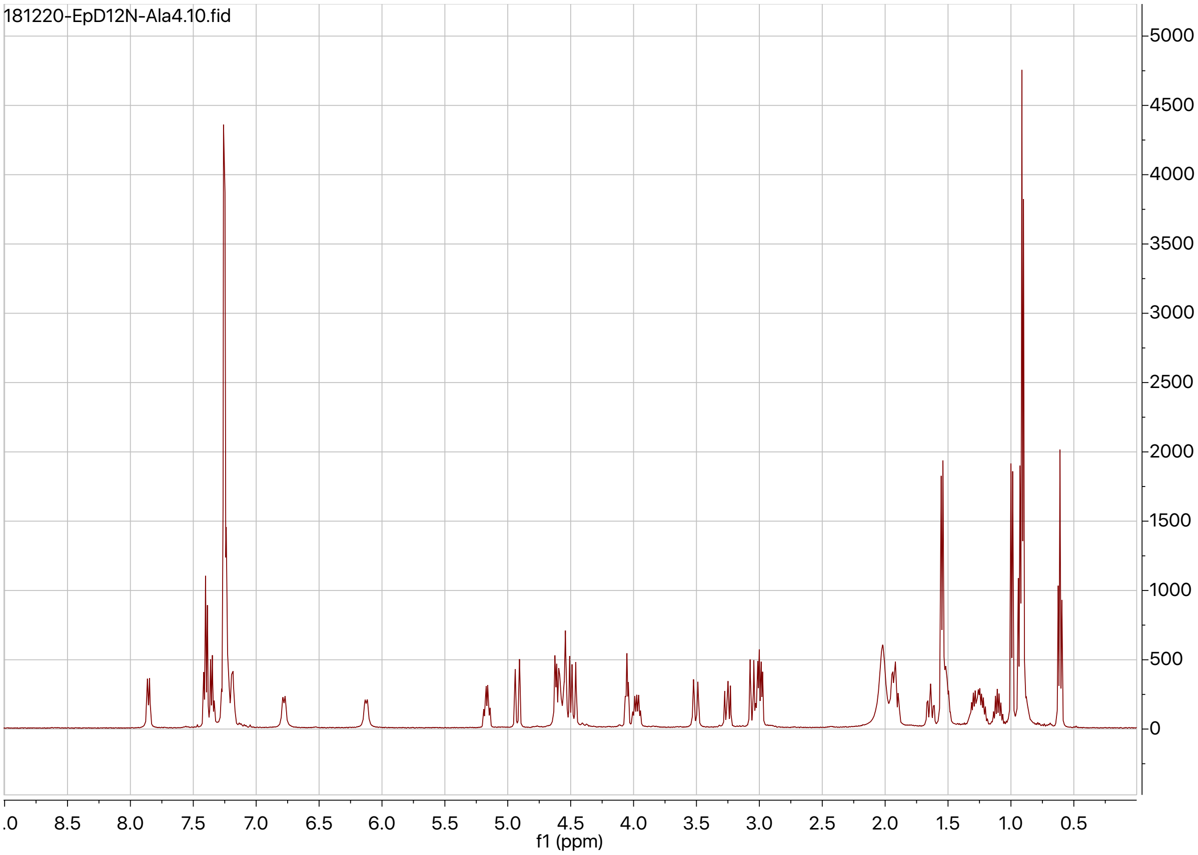

1EpDN 1Sar

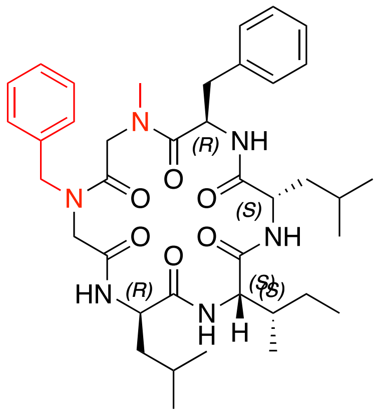

Exact Mass: 704.43
Molecular Weight: 704.91

SMILE Structure:
O=C([C@H](CC(C)C)NC([C@@]([C@@H](C)CC)([H])N1)=O)N[C@@H](C(N(CC(N(CC2=CC=CC=C2)CC(N[C@H](CC(C)C)C1=O)=O)=O)C)=O)CC3=CC=CC=C3

1EpDN 1Sar (^{1}H NMR, 500 MHz, $CDCl_3$)

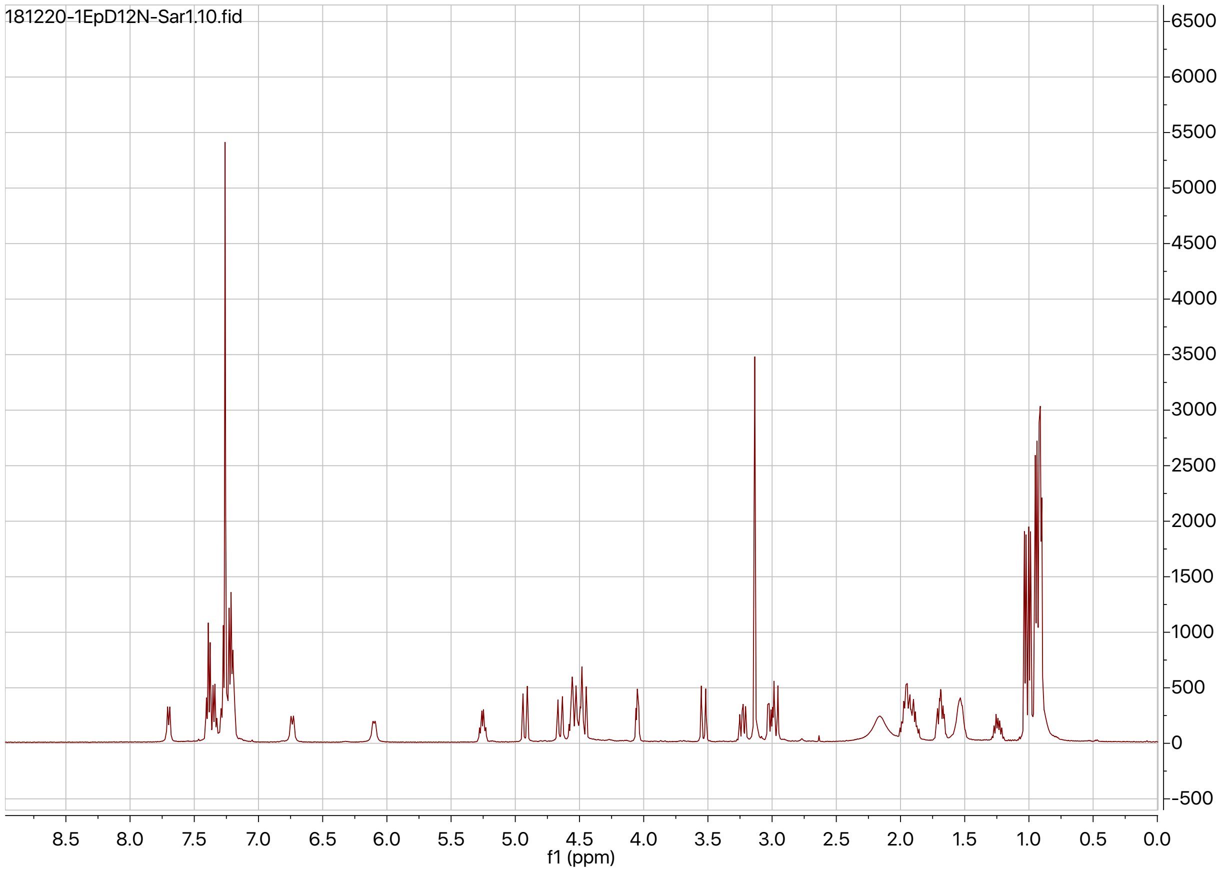

1EpDN 2Sar

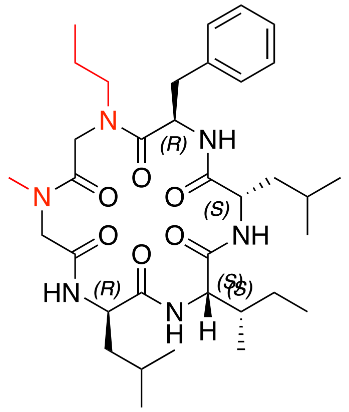

Exact Mass: 656.43
Molecular Weight: 656.87

SMILE structure:
O=C([C@H](CC(C)C)NC([C@@]([C@@H](C)CC)([H])N1)=O)N[C@@H](C(N(CC(N(C)CC(N[C@H](CC(C)C)C1=O)=O)=O)CCC)=O)CC2=CC=CC=C2

1EpDN 2Sar (^{1}H NMR, 500 MHz, $CDCl_3$)

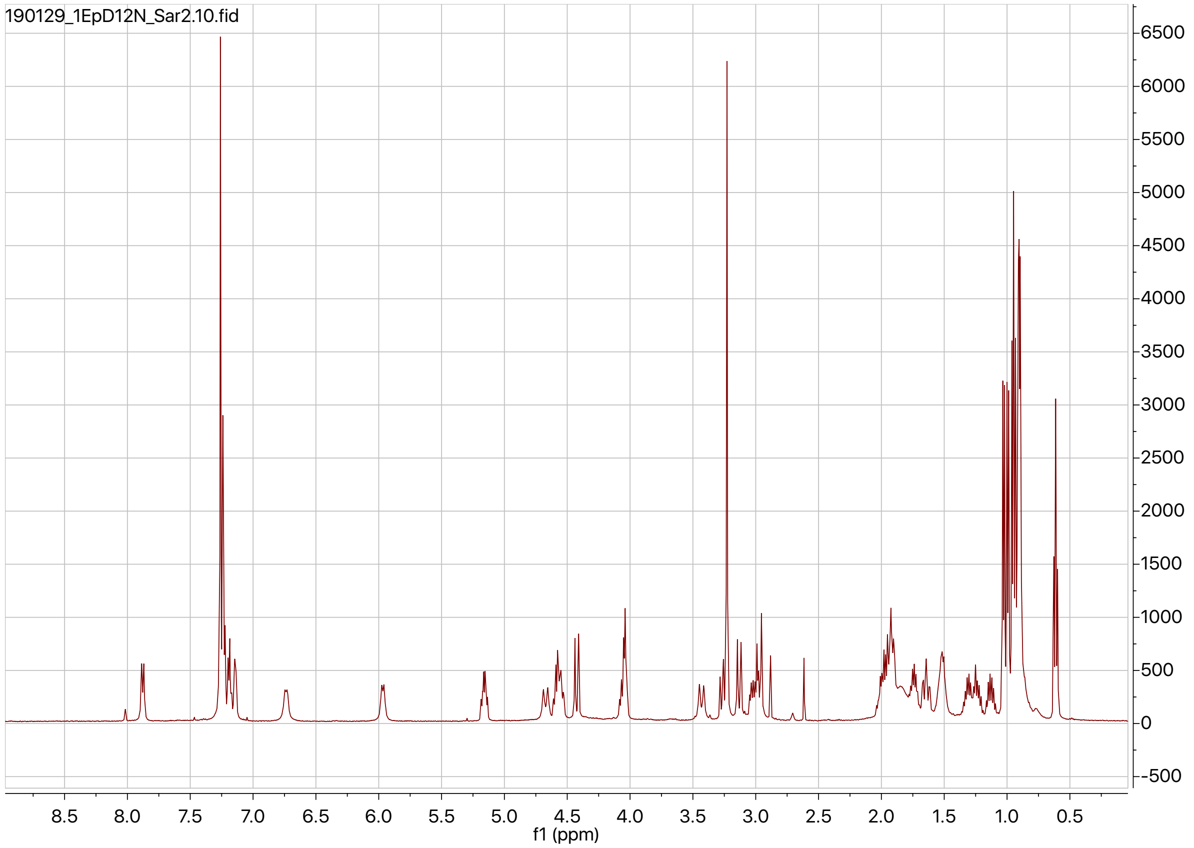

4EpDN 1Sar

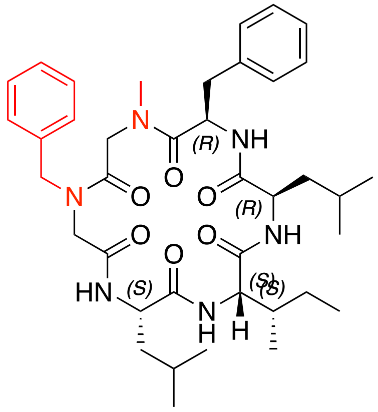

Exact Mass: 704.43
Molecular Weight: 704.91

SMILE structure:
O=C([C@@H](CC(C)C)NC([C@@]([C@@H](C)C
C)([H])N1)=O)N[C@@H](C(N(CC(N(CC2=CC=CC
=C2)CC(N[C@@H](CC(C)C)C1=O)=O)=O)C)=O)C
C3=CC=CC=C3

4EpDN 1Sar (^1H NMR, 500 MHz, $CDCl_3$)

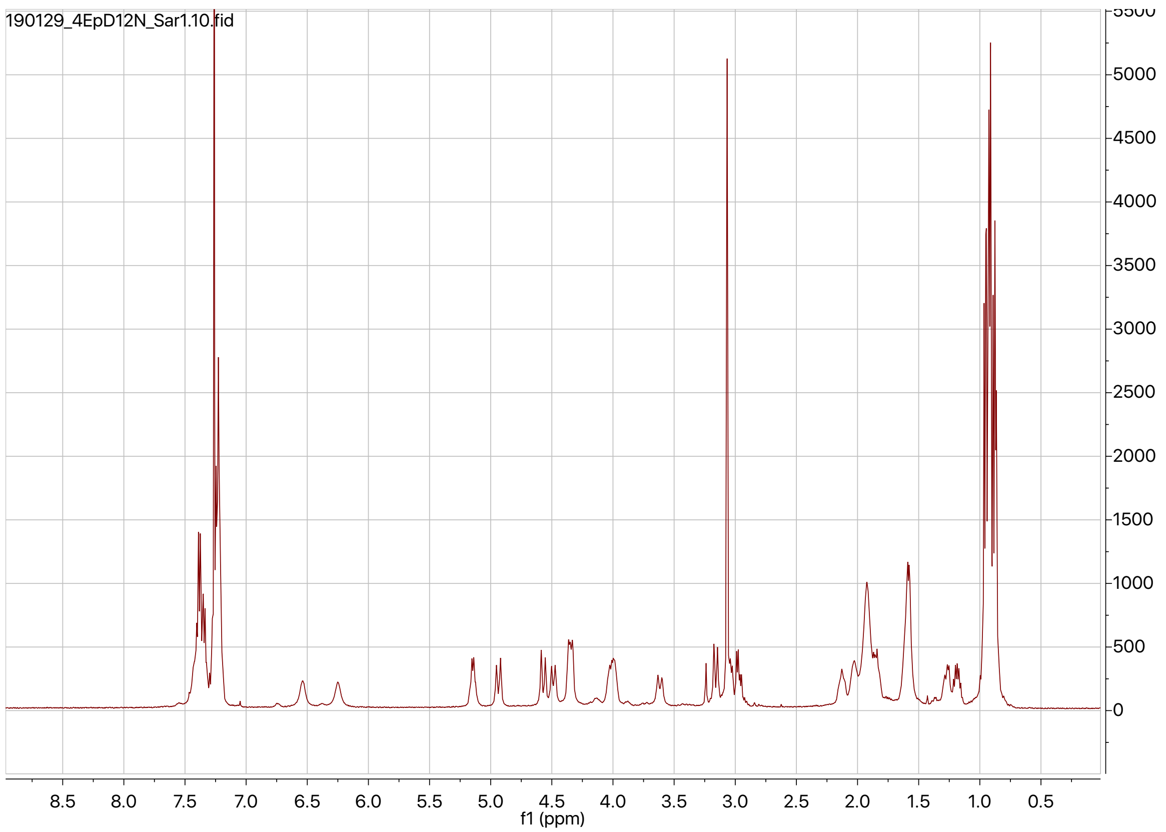

4EpDN 2Sar

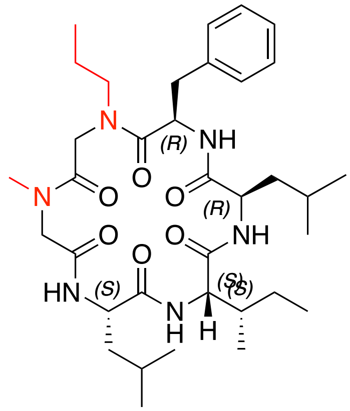

Exact Mass: 656.43
Molecular Weight: 656.87

SMILE structure:
O=C([C@@H](CC(C)C)NC([C@@]([C@@H](C)CC)([H])N1)=O)N[C@@H](C(N(CC(N(C)CC(N[C@@H](CC(C)C)C1=O)=O)=O)CCC)=O)CC2=CC=CC=C2

4EpDN 2Sar (^{1}H NMR, 500 MHz, $CDCl_3$)

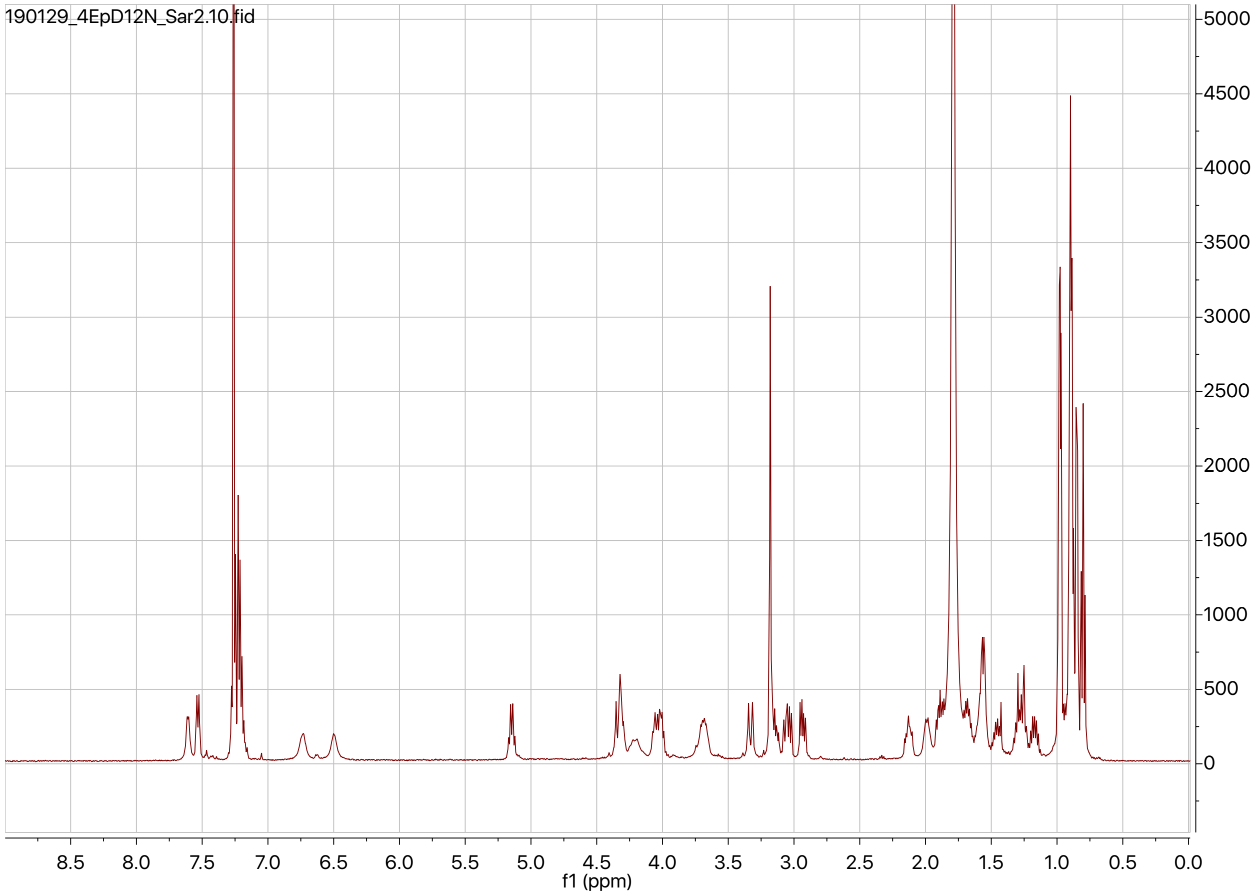

References

1. Matsui H, Eguchi M, Ohsumi K, Nakamura A, Isshiki Y, Sekiya K, Kikuchi Y, Nagamitsu T, Masuma R, Sunazuka T, Omura S. 2005. Azithromycin inhibits the formation of flagellar filaments without suppressing flagellin synthesis in Salmonella enterica serovar typhimurium. Antimicrob Agents Chemother 49:3396-403.
2. Aiello D, Williams JD, Majgier-Baranowska H, Patel I, Peet NP, Huang J, Lory S, Bowlin TL, Moir DT. 2010. Discovery and characterization of inhibitors of Pseudomonas aeruginosa type III secretion. Antimicrob Agents Chemother 54:1988-99.
3. Bowlin NO, Williams JD, Knoten CA, Torhan MC, Tashjian TF, Li B, Aiello D, Mecsas J, Hauser AR, Peet NP, Bowlin TL, Moir DT. 2014. Mutations in the Pseudomonas aeruginosa needle protein gene pscF confer resistance to phenoxyacetamide inhibitors of the type III secretion system. Antimicrob Agents Chemother 58:2211-20.
